# Application of Deuterium in an M_1_ Positive Allosteric Modulator Back-Up Program: The Discovery of VU6045422

**DOI:** 10.1021/acschemneuro.5c00119

**Published:** 2025-03-25

**Authors:** Julie L. Engers, Jinming Li, Changho Han, Madeline F. Long, Alison R. Gregro, Christopher C. Presley, Jonathan W. Dickerson, Weimin Peng, Hyekyung P. Cho, Alice L. Rodriguez, Zixiu Xiang, Olivier Boutaud, Colin O’Carroll, P. Markus Dey, Ethan S. Burstein, Colleen M. Niswender, Jerri M. Rook, P. Jeffrey Conn, Darren W. Engers, Craig W. Lindsley

**Affiliations:** † Warren Center for Neuroscience Drug Discovery, Vanderbilt University, Nashville, Tennessee 37232, United States; ‡ Department of Pharmacology, Vanderbilt University School of Medicine, Nashville, Tennessee 37232, United States; § Department of Chemistry, Vanderbilt University, Nashville, Tennessee 37232, United States; ∥ Vanderbilt Kennedy Center, Vanderbilt University Medical Center, Nashville, Tennessee 37232, United States; ⊥ Vanderbilt Brain Institute, Vanderbilt University, Nashville, Tennessee 37232, United States; # Vanderbilt Institute of Chemical Biology, Vanderbilt University, Nashville, Tennessee 37232, United States; ∇ Acadia Pharmaceuticals Inc., San Diego, California 92130, United States

**Keywords:** muscarinic acetylcholine receptor subtype 1 (M1), positive allosteric modulator (PAM), deuterium, cognition, metabolism, isotope

## Abstract

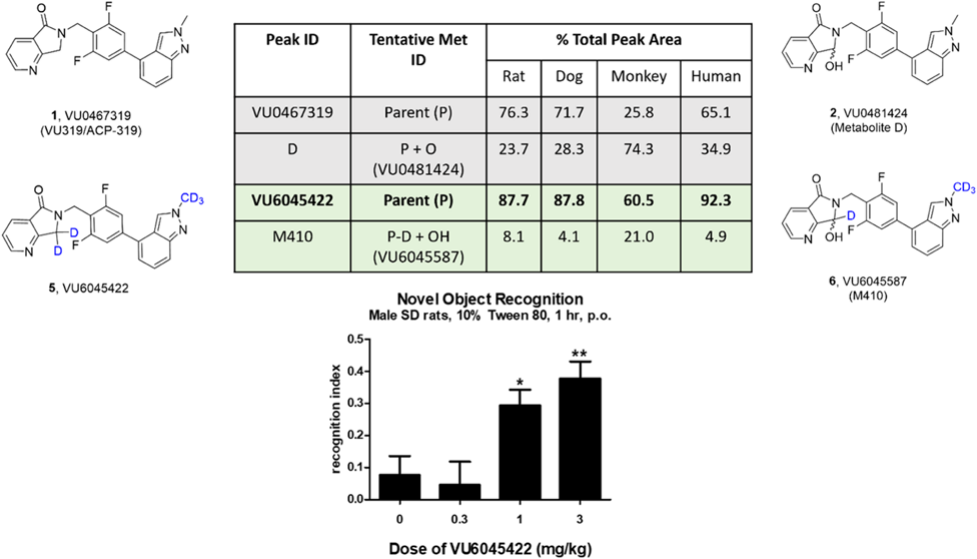

Recently, we disclosed VU0467319, an M_1_ positive allosteric modulator (PAM) clinical candidate that had successfully completed a phase I single ascending dose clinical trial. Pharmacokinetic assessment revealed that, in humans upon increasing dose, a circulating, inactive metabolite constituted a major portion of the total drug-related area under the curve (AUC). One approach the team employed to reduce inactive metabolite formation in the back-up program was the kinetic isotope effect, replacing the metabolically labile C–H bonds with shorter, more stable C–D bonds. The C–D dipole afforded VU6045422, a more potent M_1_ PAM (human EC_50_ = 192 nM, 80% ACh Max) than its proteocongener VU0467319 (human EC_50_ = 492 nM, 71% ACh Max), and retained the desired profile of minimal M_1_ agonism. Overall, the profile of VU6045422 supported advancement, as did greater *in vitro* metabolic stability in both microsomes and hepatocytes than did VU0467319. In both rat and dog *in vivo*, low doses proved to mirror the *in vitro* profile; however, at higher doses in 14-day exploratory toxicology studies, the amount of the same undesired metabolite derived from VU6045422 was equivalent to that produced from VU0467319. This unexpected IVIVC result, coupled with less than dose-proportional increases in exposure and no improvement in solubility, led to discontinuation of VU0467319/VU6045422 development.

## Introduction

**Figure 1:**
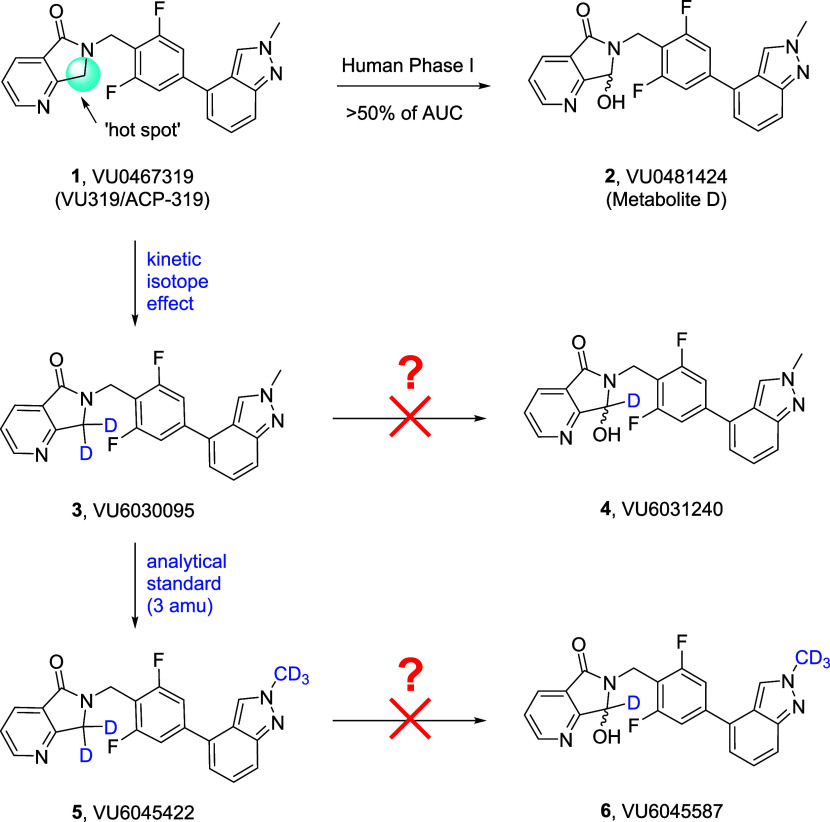
Structures of VU0467319 **1**, and the corresponding oxidative metabolite **2**, as well as the dideutero congener **3** (VU6030095) and the penta-deutero analogue **5** (VU6045422), with the putative oxidative metabolites **4** and **6**, respectively.

In the 1980s, numerous pharmaceutical companies demonstrated that “M_1_ agonists” exhibited robust efficacy in improving cognition in Alzheimer’s patients.^1−6^ However, the lack of muscarinic acetylcholine receptor selectivity among the five subtypes (M_1–5_) led to the concomitant activation of peripheral M_2_ and M_3_ receptors and contributed to significant clinically relevant cholinergic adverse events known by the acronym SLUDGE (salivation, lacrimation, urination, defecation, gastrointestinal distress, and emesis).^1−6^ Thus, developing drugs that selectively activate M_1_ muscarinic acetylcholine receptors has been a goal since that time. The advent of recombinant functional G-protein-coupled receptor (GPCR) assays made high-throughput screening for highly subtype-selective positive allosteric modulators for the M_1_ receptor possible.^7−20^ We disclosed **1**, VU0467319, an M_1_ positive allosteric modulator (PAM) clinical candidate that was chemically optimized from hits derived from an HTS campaign. VU0467319 has a profile of moderate M_1_ PAM potency and minimal M_1_ agonism and successfully completed a phase I SAD clinical trial (Figure 1).^21^ While observed preclinically in both microsome and hepatocyte preparations, as well as in preclinical species *in vivo*, in humans, the circulating, inactive metabolite **2** (VU0481424) constituted a majority (>50%) of the total drug-related area under the curve (AUC) upon increasing dose.^22−24^ As this would not only require the need for regulatory metabolite monitoring but also result in a significant loss of an active, circulating parent, the development of **1** was terminated and effort was made to identify a back-up M_1_ PAM with improved pharmacokinetic (PK) profiles.

Since M_1_ PAM **1** had the desired pharmacological profile, the team attempted numerous strategies to sterically and electronically address the metabolic “hot spot”; however, all attempts eroded M_1_ PAM potency significantly. As a final effort to salvage the chemotype, we elected to install a *gem*-dideutero moiety at the metabolic hot spot and explore if the kinetic isotope effect, with shorter, stronger C–D, would mitigate oxidative metabolism.^25,26^ This exercise led to the development of dideutero **3** (VU6030095), and a key question surrounded whether it would prove to be more stable and generate less of the analogous oxidative metabolite **4** (VU6031240). As progress began, an analytical standard was required that would be 3 atomic mass units (amu) different from **3**, and that request led to the production of the penta-deutero congener **5** (VU6045422) due to ease of incorporation of a 5 amu standard. We then characterized the pharmacological profile and metabolic stability of **5** and its propensity to produce putative oxidative metabolite **6** (VU6045587). Here, we detail the unexpected molecular pharmacology of these deuteron congeners as well as their disparate *in vitro* and *in vivo* DMPK profiles, highlighting that deuterium incorporation is not always a panacea to endow metabolic stability.

## Results and Discussion

### Design

**Scheme 1:**
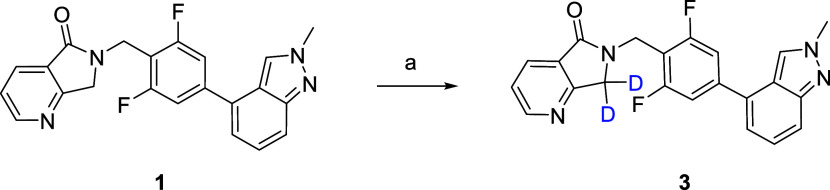
Synthesis of VU6030095 (**3**) Reagents and conditions: (a) NaOD (40 wt % in D_2_O), tetrahydrofuran (THF)/D_2_O, 35 °C, 70%.

**Scheme 2:**
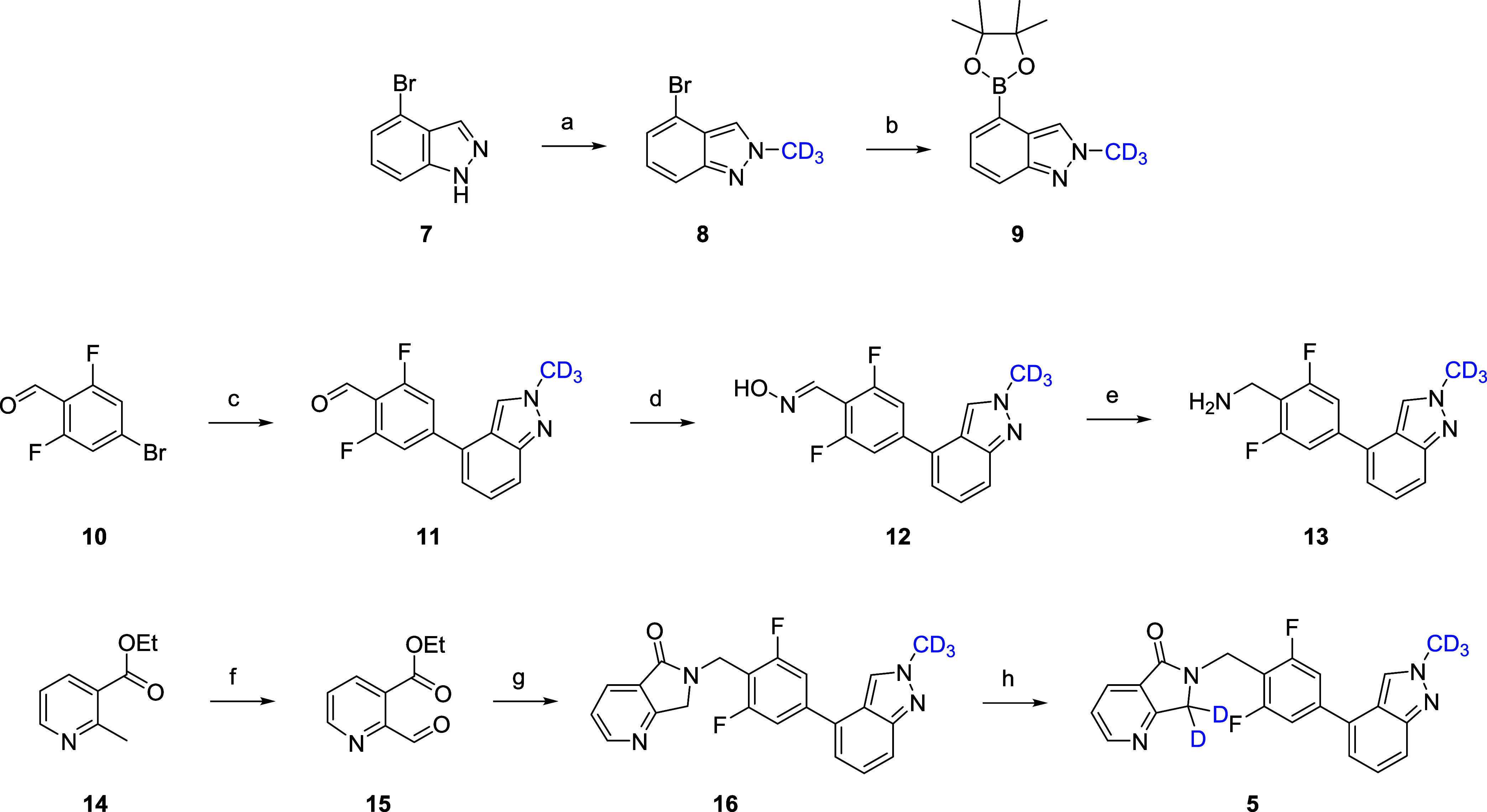
Synthesis of VU6045422 (**5**) Reagents and conditions: (a) ICD_3_, Cs_2_CO_3_, MeCN, room temperature (rt), 33%; (b) bis-pinacolborane, PdCl_2_(dppf)·CH_2_Cl_2_, KOAc, 1,4-dioxane, 100 °C, 72%; (c) **9**, PdCl_2_(dppf)·CH_2_Cl_2_, Cs_2_CO_3_, 1,4-dioxane/H_2_O, 100 °C, 96%; (d) NH_2_OH·HCl, NaOAc, EtOH, 98%; (e) Zn/HOAc, rt, 96%; (f) SeO_2_, 1,4-dioxane, molecular weight (mw), 120 °C, 68%; (g) **13**, sodium triacetoxyborohydride (STAB), dichloroethane (DCE), 89%; (h) NaOD (40 wt % in D_2_O), THF/D_2_O, 45 °C, 73%.

As alluded to previously, attempts to block undesired oxidative metabolism at the benzylic lactam center, employing either steric or electronic strategies, were unsuccessful, resulting in a complete loss of the M_1_ PAM potency. Thus, we evaluated the impact of deuterium incorporation at the metabolic hot spot to assess if the shorter, stronger C–D bond would yield improved stability (i.e., the kinetic isotope effect).^25,26^ To access the desired *gem*-dideutero derivative, a concise strategy was developed (Scheme 1), wherein **1** was treated with NaOD in D_2_O to cleanly afford **3** (VU6030095) in 70% yield with high isotopic purity. Due to the stage of the program, a request was made to produce an analytical standard 3 amu different from **3**, which led to the more complex, convergent synthesis (Scheme 2) of **5** (VU6045422) harboring not only the *gem*-dideutero moiety of **3**, but an additional *N*-CD_3_ on the indazole. Starting from commercial bromo indazole **7**, alkylation with CD_3_I affords the *N*-CD_3_ indazole **8** in 33% yield. Conversion of **8** into pinacol borate under standard conditions provides the Suzuki coupling partner **9** in 72% yield. Commercial aldehyde **10** participates in a Suzuki coupling reaction with **9** to afford biaryl aldehyde **11** in 96% yield. The requisite benzyl amine **13** is accessed in two high yielding streps by the conversion of aldehyde **11** to oxime **12**, followed by Zn-catalyzed reduction. Pyridine **14** undergoes benzylic oxidation with SeO_2_ to deliver aldehyde **14**, which then undergoes a reductive amination/cyclization sequence to give lactam **16** in high yield. As for **3**, exposure of **16** to NaOD in D_2_O cleanly afforded **5** (VU6045422) in 73% yield and high isotopic purity. Thus, the team had the necessary new ligands **3** and **5** to assess metabolic stability as well as M_1_ PAM activity; however, we also needed to synthesize known metabolite **2**, as well as the putative metabolites **4** and **6** to provide analytical standards for quantification *in vitro* and *in vivo*.

**Scheme 3:**
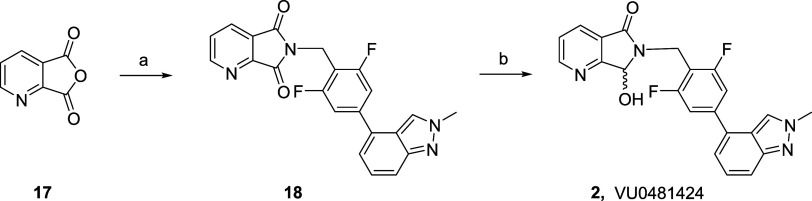
Synthesis of VU0481424 (**2**) Reagents and conditions: (a) (2,6-difluoro-4-(2-methyl-2*H*-indazol-4-yl)phenyl)amine, Et_3_N, dimethylformamide (DMF)/MeCN, rt, 1 h, then 1-[bis(dimethylamino)methylene]-1*H*-1,2,3-triazolo[4,5-*b*]pyridinium 3-oxide hexafluorophosphate (HATU), 90%; (b) NaBH_4_, DCM/MeOH, 24%.

**Scheme 4:**
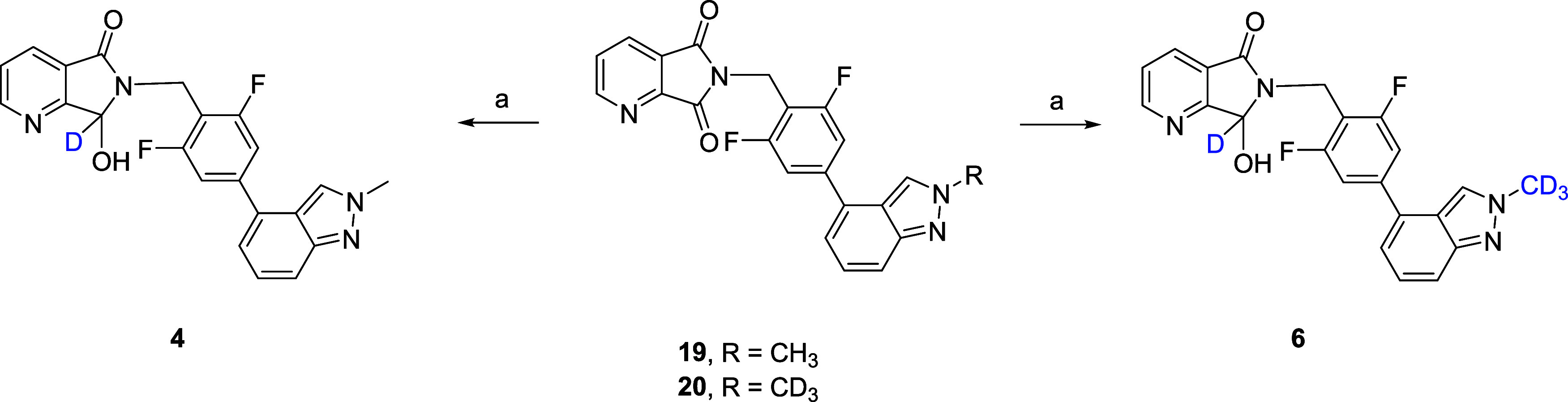
Synthesis of VU6031240 (**4**) and VU6045587 (**6**) Reagents and conditions: (a) NaBD_4_, DCM/MeOD, 30–35%.

The major metabolite **2** of **1** was made into a two-step sequence employing the key benzyl amine used to construct **1** (Scheme 3). Starting from commercial anhydride **17**, treatment with functionalized benzyl amine provides phthalimide **18** in 90% yield. Chemoselective reduction with NaBH_4_ provides racemic metabolite **2** in a 24% isolated yield. In a similar fashion (Scheme 4), chemoselective reduction of **19** and **20** with NaBD_4_ in dichloromethane (DCM)/MeOD provides **4** and **6**, respectively, in 30–35% isolated yields. Like **2**, putative metabolites **4** and **6** were inactive as M_1_ PAMs.

### Hepatocyte Stability (*In Vitro*)

**Figure 2:**
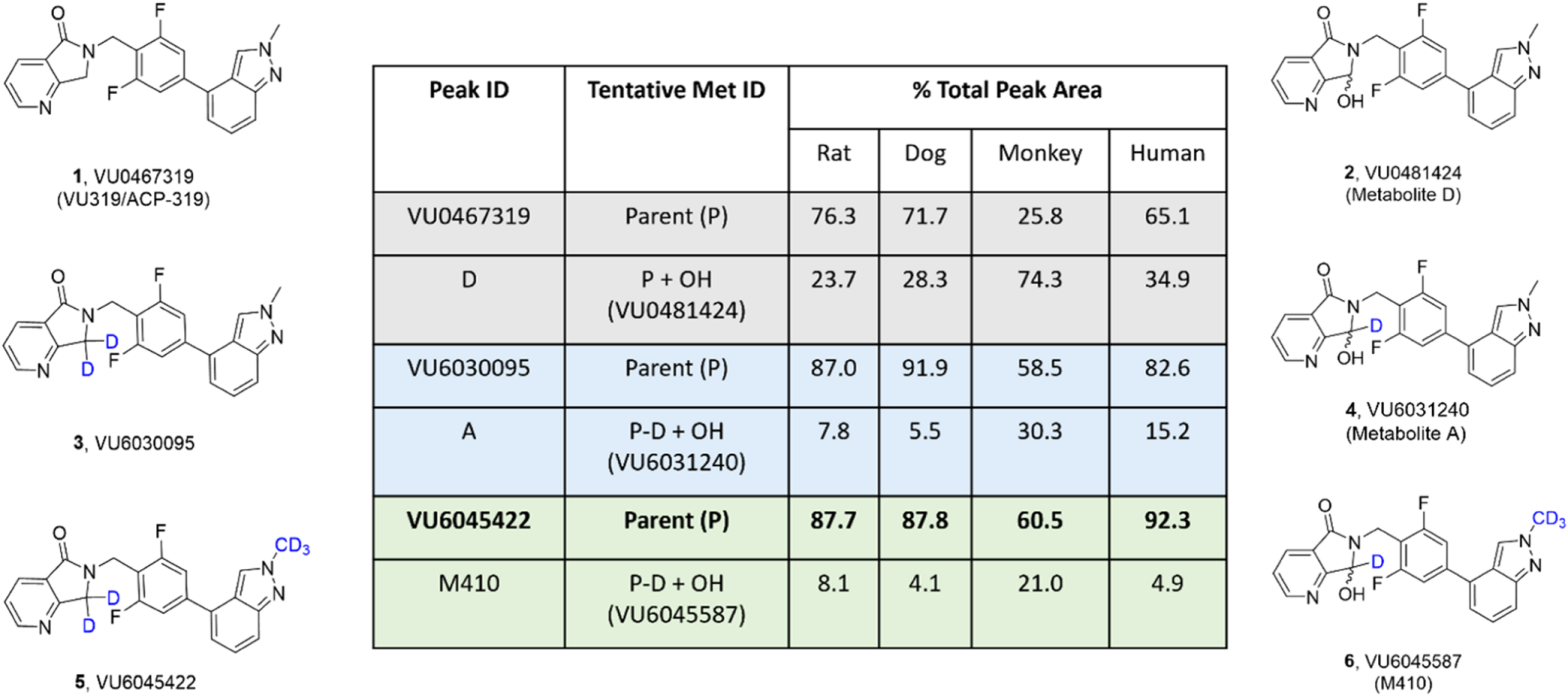
Multispecies hepatocyte metabolite identification of M_1_ PAMs **1**, **3**, and **5** and quantification of oxidative metabolites **2**, **4**, and **6**, respectively. *In vitro* hepatocyte assays clearly indicated greater stability toward oxidative metabolism for the deuterated congeners.

Prior to full characterization of deuterated analogues **3** and **5**, they were incubated in rat, dog, monkey, and human hepatocytes, along with **1**, to determine the amount of parent remaining, as well as the degree of undesired oxidative metabolites **2**, **4**, and **6** (Figure 2). For **1**, metabolite **2** was generated between 23.7 and 34.9% across rat, dog, and humans and was the major species in monkey (74.3%). For the *gem*-dideutero congener **3**, metabolic stability was improved *in vitro*. In humans, production of the corresponding oxidative metabolite **4** was reduced 50% (34.9–15.2%) and both rat (7.8%) and dog (5.5%) were low, while in monkey there was a similar reduction >50% (74.3–30.3%). Surprisingly, the penta-deutero derivative **5** demonstrated exceptional stability across species, affording low levels of the analogous metabolite **6** in rat (8.1%), dog (4.1%), monkey (21%) and humans (4.9%). Specifically, whereas 34.9% of **1** was metabolized to **2** in human hepatocytes, **6** displayed a >80% reduction in metabolite production (4.9%). Thus, in hepatocytes *in vitro*, the kinetic isotope effect engendered greater metabolic stability of the labile hot spot on the lactam. It remained to be seen if this effect would translate *in vivo*, but we first had to assess the impact of the deuterated analogues on molecular pharmacology, *in vitro* DMPK, standard low-dose *in vivo* DMPK and, if warranted, *in vivo* efficacy in a rat novel object recognition (NOR) behavioral assay.

### Molecular Pharmacology and *In Vitro* DMPK

As we only introduced deuterium into the VU319/ACP-319 (**1**)^21^ core with **3** and **5**, we anticipated comparable M_1_ PAM pharmacology (Table 1). Interestingly, the *gem*-dideutero **3** was less potent (human EC_50_ = 624 nM, 81%; rat EC_50_ = 361 nM, 85%) than **1** (human EC_50_ = 492 nM, 80%; rat EC_50_ = 213 nM, 79%), yet maintained the desired profile of minimal M_1_ agonism and selectivity versus M_2–5_ (>30 μM).^21^ The loss of M_1_ PAM potency could be the result of a C–D dipole that diminishes the receptor affinity. The addition of the *N*-CD_3_ moiety in **5** resulted in a more potent human M_1_ PAM (EC_50_ = 192 nM, 80%) and equipotent rat M_1_ PAM (EC_50_ = 249 nM, 82%), again with minimal M_1_ agonism and selectivity versus M_2–5_ (>30 μM). Perhaps in this case, the C–D dipole of the CD_3_ moiety engages the receptor more effectively. Interestingly, in hepatic microsomes, predicted hepatic clearance was significantly lower for the parent **1** across species than either deutero derivative **3** or **5**, while the fraction unbound in plasma and brain was equivalent across the three M_1_ PAMs. CYP_450_ profiles were comparable for the three compounds, as were MDCK-MDR1 (P-gp) ER (1.3–1.8) with high *P*_app_ ((31–41) × 10^–6^ cm/s).

**Table 1:**
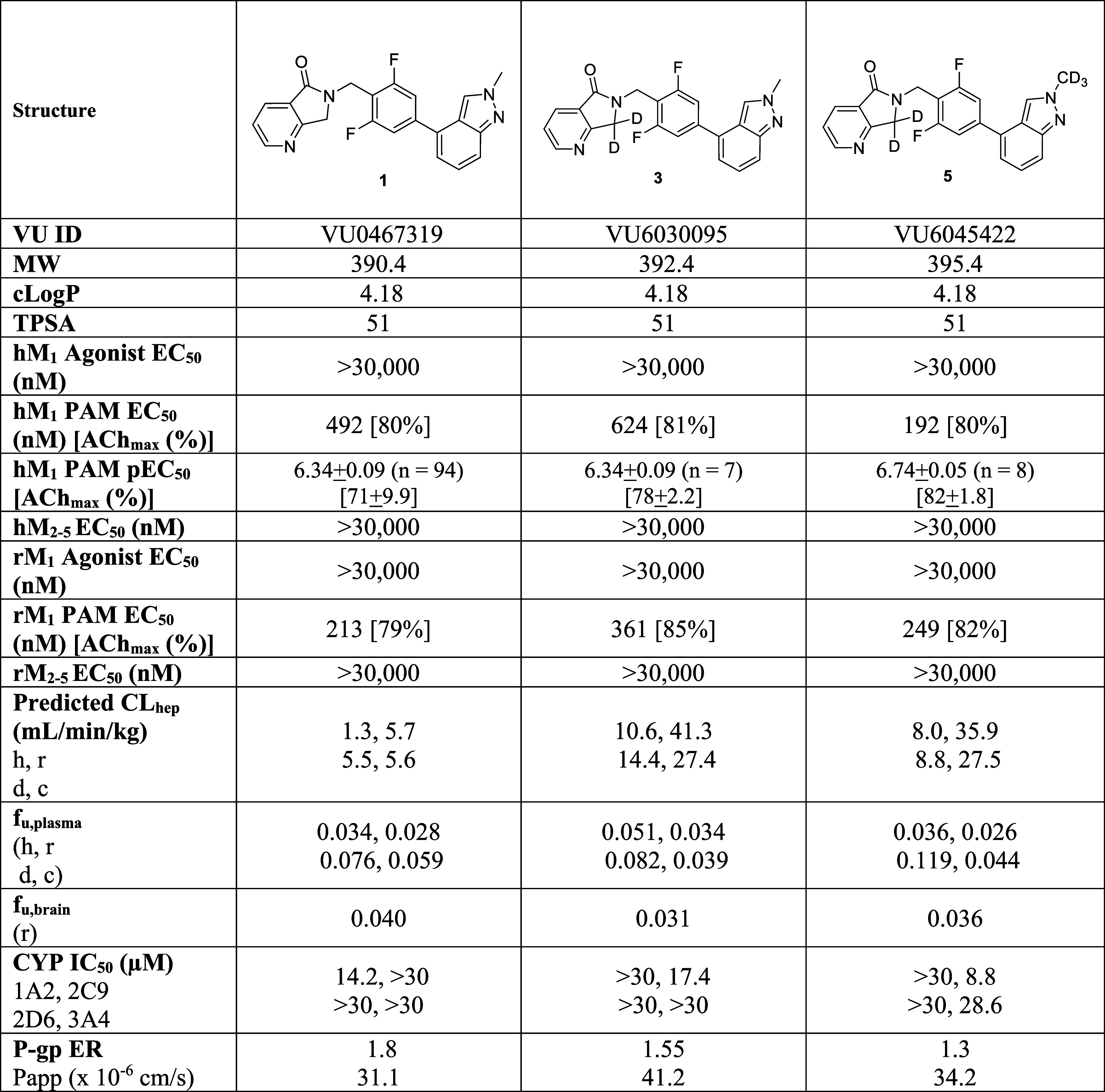
Pharmacology and *In Vitro* DMPK Profiles of M_1_ PAMs **1**, **3**, and **5**

### *In Vitro* Electrophysiology

**Figure 3:**
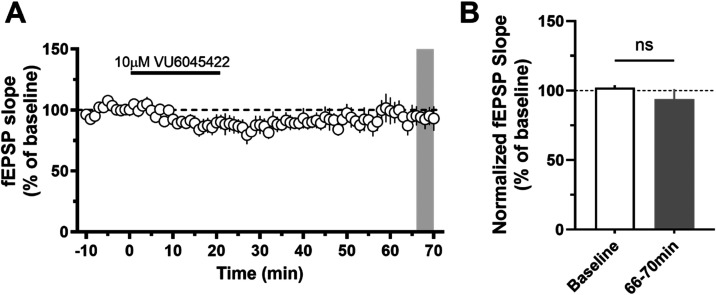
M_1_ PAM **5** (VU6045422) did not induce a significant long-term depression of fEPSPs in the mouse layer 5 prefrontal cortex. (A) Normalized time course of the fEPSP slope during baseline, application of 10 μM VU6045422 and washout. (B) Bar graph summarizing the averaged fEPSP slope during the last 5 min of washout (gray bar in panel (A)) compared to the baseline period (ns, *p* > 0.05, two-tailed paired *t* test, *n* = 9).

We carried out electrophysiology studies in mouse native tissue layer V medial prefrontal cortex (mPFC) to ensure that **5** did not induce long-term depression (LTD). While ago-PAMs induce substantial long-term depression that correlates with a lack of robust procognitive efficacy,^14,16−21^ PAM **5** demonstrated no significant changes in field excitatory post synaptic potentials (fEPSPs) recorded from layer V and evoked by electrical stimulation in layer II/III at 10 μM concentration (∼40× above the functional mouse EC_50_). Thus, PAM **5** maintains activity dependence of PFC function (Figure 3), and it is expected to display robust precognitive efficacy.

### *In Vivo* DMPK and Behavior

**Figure 4:**
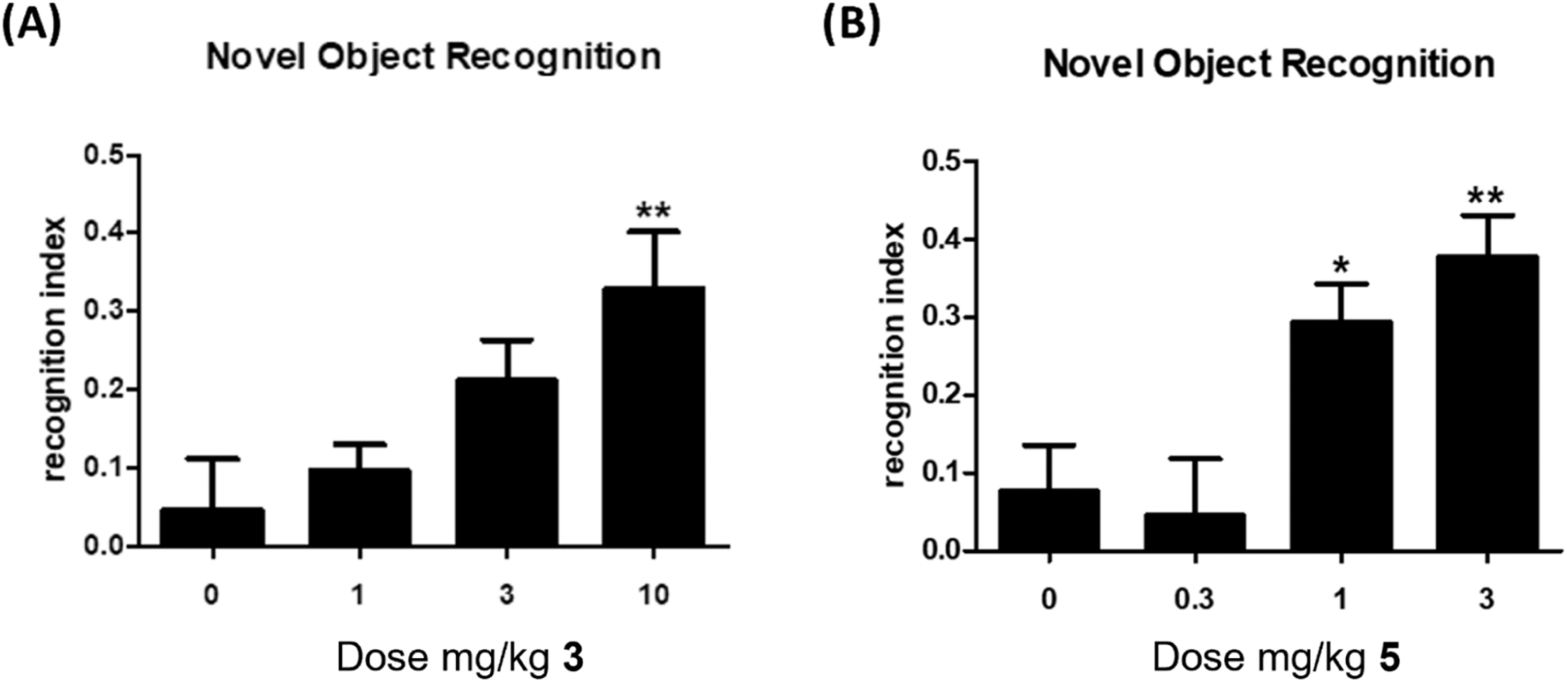
Novel object recognition (NOR) test in rats with M_1_ PAMs **3** and **5**. (A) PAM **3** dose-dependently enhanced the recognition memory in rats. Pretreatment with 1, 3, and 10 mg/kg of **3** (PO 0.5% Natrasol/0.015% Tween 80 in water) 2 h prior to exposure to identical objects significantly enhanced recognition memory assessed 24 h later. Minimum effective dose (MED) is 10 mg/kg. (B) PAM **5** dose-dependently enhanced recognition memory in rats. Pretreatment with 0.3, 1, and 3 mg/kg of **5** (PO 10% Tween 80 in water) 1 h prior to exposure to identical objects significantly enhanced recognition memory assessed 24 h later. Minimum effective dose (MED) is 1 mg/kg. *N* = 13–18/group of male Sprague-Dawley rats. Analysis of variance (ANOVA) *p < 0.05; **p < 0.01 Dunnett posthoc test.

As a reference in rat, **1** displayed an attractive profile (CL_p_ = 3 mL/min/kg, *t*_1/2_ = 3 h, *V*_ss_ = 0.67 L/kg and with 80% F) and a robust *in vitro*/*in vivo* correlation (IVIVC, e.g., rat CL_hep_ = 5.7 mL/min/kg).^21^ Moreover, **1** showed good central nervous system (CNS) distribution in rat (*K*_p_ = 0.64; *K*_p,uu_ = 0.91).^21^ The *gem*-dideutero congener **3** (Table 2) showed a very similar *in vivo* profile to **1** in rat (CL_p_ = 5.5 mL/min/kg, *t*_1/2_ = 3 h, *V*_ss_ = 1.4 L/kg and with 100% F), with a poor IVIVC (CL_p_ = 5.5 mL/min/kg vs rat CL_hep_ = 41.3 mL/min/kg), but good CNS exposure (*K*_p_ = 0.63; *K*_p,uu_ = 0.57). Once again, a poor IVIVC was noted for penta-deutero analogue **5**, but the *in vivo* DMPK profile was excellent (CL_p_ = 4.8 mL/min/kg, *t*_1/2_ = 3.6 h, *V*_ss_ = 1.1 L/kg, 100% F, *K*_p_ = 1.1; *K*_p,uu_ = 1.5). Interestingly, there were significant disconnects between the stability noted for **3** and **5** in hepatic microsomes, hepatocytes, and *in vivo*. As **3** and **5** were comparable in disposition, but **3** was a weaker PAM, the team decided to compare *in vivo* efficacy of these two PAMs in a rat novel object recognition (NOR) assay (Figure 4) to validate the differential M_1_ PAM potency. While both M_1_ PAMs afforded a dose-dependent increase in NOR, **5** was more efficacious (minimum effective dose (MED) = 1 mg/kg) compared to **3** (MED = 10 mg/kg). The *in vitro* and *in vivo* potency of **5** elevated it as a potential candidate, and deeper profiling was initiated.

**Table 2:** *In Vivo* Pharmacokinetic Profiles of M_1_ PAMs **3** and **5**

compound	**1**	**1**	**1**	**3**	**3**	**3**	**5**	**5**	**5**
parameter	rat (SD)	dog (beagle or mongrel)	NHP (cyno)	rat (SD)	dog (beagle or mongrel)	NHP (cyno)	rat (SD)	dog (beagle or mongrel)	NHP (cyno)
dose (mg/kg) iv/po	1/3	1/3	1/3	1/10			1/10	1/5	1/5
CL_p_ (mL/min/kg)	3.0	4.0	3.3	5.5			4.8	2.6	4.0
*V*_ss_ (L/kg)	0.67	2.1	0.9	1.4			1.1	2.0	1.1
elimination *t*_1/2_ (h)	3.0	7.5	4.3	3.0			3.6	10.0	4.6
*F* (%) po	93	100	59	100			100	85	64
*K*_p_	0.77			0.63			1.1		
*K*_p,uu_	1.3			0.57			1.5		

**Figure 5:**
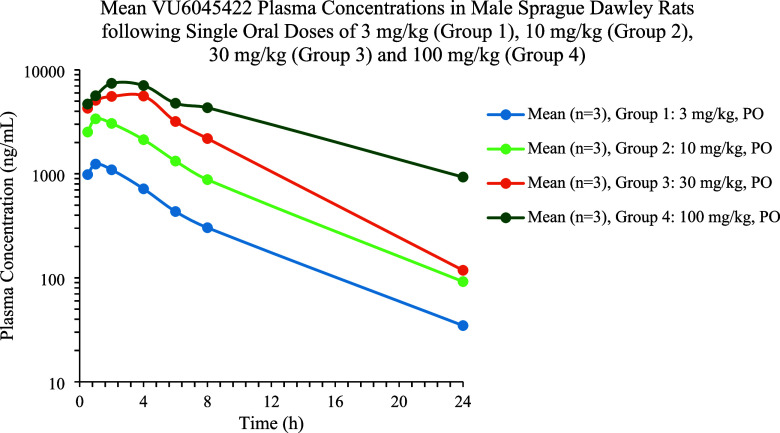
Rat oral dose escalation PK study at 3 (AUC last 8390 h·ng/mL; *C*_max_ = 1250 ng/mL), 10 (AUC last 23,900 h·ng/mL; *C*_max_ = 3380 ng/mL), 30 (AUC last 55,600 h·ng/mL; *C*_max_ = 6020 ng/mL), and 100 mg/kg (AUC last 87,800 h·ng/mL; *C*_max_ = 7850 ng/mL).

As shown in Table 2, M_1_ PAM **5** possessed attractive pharmacokinetic properties in dog (CL_p_ = 2.6 mL/min/kg, *t*_1/2_ = 10 h, V_ss_ = 2.0 L/kg, 85% F) and Cynomolgus monkey (CL_p_ = 4.0 mL/min/kg, *t*_1/2_ = 4.6 h, *V*_ss_ = 1.1 L/kg, 64% F). Whereas **1** had a hERG manual patch clamp IC_50_ of 12 μM,^21^ PAM **5** had an improved hERG manual patch clamp potency (IC_50_) of 21 μM. In addition, **5** was negative in the bacterial mutagenicity mini-AMES assay (4-strain with and without S9), had no significant activity in a Eurofins lead profiling screen (evaluating potential for “off-target” binding) of 68 GPCRs, ion channels, and transporters (no activity >50%@10 μM except: 58%@10 μM at α_2A_ and 59%@10 μM at rat imidazoline), and evidence only a trace (<0.1%) of conjugation in GSH trapping studies (rat, dog, cyno, and human microsomes). PAM **1** was solely metabolized by CYP 3A4;^21^ in contrast, other CYP isoforms contributed to metabolizing PAM **5** (3A4 (92.6%), 2D6 (1.6%), and 3A5 (5.8%)). Moreover, there were no time-dependent CYP inhibition or CYP induction liabilities identified; **5** had minimal bile salt export pump (BSEP) inhibition (IC_50_ = 42.3 μM). Finally, at a 100 mg/kg IP dose in mice, a minimal Racine score of 1 was noted for M_1_ PAM **5**. In a rat oral dose escalation PK study, sublinear dose escalation using suspension-based formulations in vehicle was observed (3–30 mg/kg); while dose-related increases in exposure were seen, the 100 mg/kg cohort was not linear (Figure 5). Despite high exposures obtained in this study, no cholinergic signs or adverse events were noted.

As disconnects were observed with metabolic stability *in vitro* (in microsomes and hepatocytes) and *in vivo*, we had to ensure that **5** differentiated from **1** and would not produce high levels of metabolite **6***in vivo*. To this end, we prepared a 200 g lot of PAM **5** and performed, in parallel, a dog maximum tolerated dose (MTD) with a pharmacokinetics study and a 14-day rat exploratory toxicology and toxicokinetic study, quantifying plasma *C*_max_ and AUC for both parent **5** and metabolite **6**. In the dog MTD (dosed at 30, 100, and 1000 mg/kg of PO), PAM **5** was well-tolerated, but at all three dose levels, significant levels of **6** were detected (17–23% of *C*_max_ concentration and 18–21% of the AUC), far above what hepatocyte incubations predicted (Table 3). Moreover, while exposure increased with dose, it was nonlinear. Unfortunately, in the 14-day rat toxicology and toxicokinetic study (dosed at 300, 750, and 1500 mg/kg of PO using suspension-based formulations), even higher levels of **6** (Table 4) were detected at day 14 in terms of both *C*_max_ (23–32%) and AUC (23–28%). At lower doses, exposure was nonlinear and exposure of **5** decreased, while the exposure of **6** increased (both *C*_max_ and AUC) at the 1500 mg/kg dose. Based on these *in vivo* data sets, it was clear that **5** did not differentiate from **1** in terms of the amount of circulating oxidative metabolite **6**, and the levels were likely to be higher in humans, requiring monitoring. The series was terminated based on (1) poor and less than dose-proportional increases in systemic exposure, (2) lack of a kinetic isotope effect to diminish production of **6** in dogs and rats, and (3) strong likelihood of even higher concentrations of **6** to be present in man.

**Table 3:** Dog MTD Study Concentrations of **5** and **6**

	**5**		**6**		ratio	
dose, (mg/kg PO)	*C*_max_	AUC	*C*_max_	AUC	*C*_max_ (%)	AUC (%)
30	2400	34,858	455	6242	19	18
300	3620	67,985	818	14,120	23	21
1000	5560	103,306	972	19,158	17	19

**Table 4:** Rat 14-Day Toxicology Study Concentrations of **5** and **6** on Day 14

	**5**		**6**		ratio	
dose, (mg/kg PO)	*C*_max_	AUC	*C*_max_	AUC	*C*_max_ (%)	AUC (%)
300	22,200	321,000	6740	89,500	30	28
750	31,300	536,000	7340	125,000	23	23
1500	30,700	518,000	9890	143,000	32	28

## Conclusions

A chemical optimization back-up program for VU0467319, an M_1_ PAM clinical candidate that successfully completed a phase I SAD clinical trial, is reported. In an attempt to diminish a circulating, inactive metabolite that constituted a significant portion of the AUC upon increasing dose in humans, we focused on C–D bioisosteres and application of the kinetic isotope effect. From this effort, a penta-deutero congener, VU6045422, was developed and evaluated. The C–D dipole afforded a more potent M_1_ PAM (human EC_50_ = 192 nM, 80% ACh Max) than its proteocongener VU0467319 (human EC_50_ = 492 nM, 71% ACh Max) and retained the desired profile of minimal M_1_ agonism. In 4 h multispecies hepatocyte incubations, VU6045422 was significantly more stable than VU0467319, but was found less stable in liver microsomes. Low dose *in vivo* PK mirrored the enhanced hepatocyte stability. In rat NOR, VU6045422 displayed an MED of 1 mg/kg PO and excellent multispecies IV/PO PK and high CNS penetration. However, in a dog MTD and pharmacokinetic study, as well as in a rat 14-day toxicology and toxicokinetics study at high doses, the amount of VU6045422 oxidative metabolism did not differentiate significantly from that of VU0467319, and higher levels were projected in humans. This unexpected IVIVC result, coupled with less than dose-proportional increases in exposure and no improvement in solubility, led to the cessation of VU6045422 development. This work highlights the challenges in C–D bioisosterism and identifies that the application of this strategy is not always a panacea. However, VU6045422 represents another exceptional M_1_ PAM tool compound, with minimal agonism, to study selective M_1_ activation in rodent models.

## Supporting Information Available

Additional experimental details, methods for the synthesis and characterization of all compounds (^1^H NMR, ^13^C NMR, HRMS, LCMS), *in vitro* and *in vivo* DMPK protocols, and supporting figures (PDF)

## Abbreviations

PAM: positive allosteric modulator
PBL: plasma/brain level
DMPK: drug metabolism and pharmacokinetics
AE: adverse event
M_1_: muscarinic acetylcholine receptor subtype 1
MED: minimum effective dose
NOR: novel object recognition
IND: investigational new drug
FDA: federal drug administration
