# Supplementary material for: Application of Deuterium in an M1 Positive Allosteric Modulator Back-Up Program: The Discovery of VU6045422
Source: ACS Chem Neurosci. 2025 Mar 25;16(8):1582–91. doi: 10.1021/acschemneuro.5c00119 (PMC12006963; doi:10.1021/acschemneuro.5c00119)
Supplement: Supplementary file 1 — cn5c00119_si_001.pdf [file cn5c00119_si_001.pdf]

Application of deuterium in an M_1 Positive Allosteric Modulator back-up program: the discovery of VU6045422

Julie L. Engers,[1,2] Jinming Li,[1,2] Changho Han,[1,2] Madeline F. Long,[1,2] Alison R. Gregro,[1,2] Christopher C. Presley,[1,2] Jonathan W. Dickerson,[1,2] Weimin Peng,[1,2] Hyekyung P. Cho,[1,2] Alice L. Rodriguez,[1,2] Zixiu Xiang,[1,2] Olivier Boutaud,[1,2] Colin O'Carroll[7], P. Markus Dey,[7] Ethan S. Burstein,[7] Colleen M. Niswender,[1,2,4,5,6] Jerri M. Rook,[1,2] P. Jeffrey Conn,[1,2,4] Darren W. Engers,[1,2] and Craig W. Lindsley*[1,2,3,4]

Affiliation:
[1]Warren Center for Neuroscience Drug Discovery, Vanderbilt University, Nashville, TN 37232, USA
[2]Department of Pharmacology, Vanderbilt University School of Medicine, Nashville, TN 37232, USA
[3]Department of Chemistry, Vanderbilt University, Nashville TN 37232, USA
[4]Vanderbilt Kennedy Center, Vanderbilt University Medical Center, Nashville, TN 37232, USA
[5]Vanderbilt Brain Institute, Vanderbilt University, Nashville, TN 37232, USA
[6]Vanderbilt Institute of Chemical Biology, Vanderbilt University, Nashville, TN 37232, USA
[7]Acadia Pharmaceuticals Inc., San Diego, CA 92130, USA

*To whom correspondence should be addressed at craig.lindsley@vanderbilt.edu

TABLE OF CONTENTS

Eurofins Lead Profiling Screen....S2
Procedures for Biological Experiments....S4
General Methods....S10
Synthetic Procedures and Spectra....S12
Supplemental Figures....S46

Table S1. Eurofins Lead Profiling Screen Data - Study TW04-008508

This is a radioligand binding panel of 67 targets including GPCRs, ion channels, transporters and nuclear hormones. Biochemical assay results are presented as the percent inhibition of specific binding at a 10 µM concentration of **VU6045422** (**5**).

**Target/Protein**	**Species**	**% Inhibition at 10 µM**
Adenosine A_1	Human	30
Adenosine A_{2A}	Human	7
Adenosine A_3	Human	12
Adrenergic α_{1A}	Human	8
Adrenergic α_{1B}	Human	10
Adrenergic α_{1D}	Human	-15
Adrenergic α_{2A}	Human	58
Adrenergic β_1	Human	2
Adrenergic β_2	Human	-1
Androgen (Testosterone)	Human	0
Bradykinin B_1	Human	13
Bradykinin B_2	Human	2
Calcium Channel L-Type, Benzothiazepine	Rat	15
Calcium Channel L-Type, Dihydropyridine	Rat	6
Calcium Channel N-Type	Rat	-2
Cannabinoid CB_1	Human	-3
Dopamine D_1	Human	-3
Dopamine D_{2S}	Human	2
Dopamine D_3	Human	-1
Dopamine $D_{4.2}$	Human	13
Endothelin ET_A	Human	-4
Endothelin ET_B	Human	1
Epidermal Growth Factor (EGF)	Human	3
Estrogen ERα	Human	10
$GABA_A$, Flunitrazepam, Central	Rat	0
$GABA_A$, Muscimol, Central	Rat	0
$GABA_{B1A}$	Human	-5
Glucocorticoid	Human	13
Glutamate, Kainate	Rat	13
Glutamate, NMDA, Agonism	Rat	3
Glutamate, NMDA, Glycine	Rat	-8
Glutamate, NMDA, Phencyclidine	Rat	-1
Histamine H_1	Human	2
Histamine H_2	Human	2
Histamine H_3	Human	5
Imidazoline I_2, Central	Rat	59

Interleukin IL-1 R1	Human	8
Leukotriene, Cysteinyl $CysLT_1$	Human	-9
Melatonin MT_1	Human	5
Muscarinic M_1	Human	0
Muscarinic M_2	Human	5
Muscarinic M_3	Human	-10
Neuropeptide Y Y_1	Human	-13
Neuropeptide Y Y_2	Human	-3
Nicotinic Acetylcholine α3β4	Human	-7
Nicotinic Acetylcholine α1, Bungarotoxin	Human	8
Opiate δ_1 (OP1, DOP)	Human	1
Opiate κ (OP2, KOP)	Human	9
Opiate μ (OP3, MOP)	Human	5
Phorbol Ester	Mouse	5
Platelet Activating Factor (PAF)	Human	22
Potassium Channel [K_{ATP}]	Hamster	24
Potassium Channel hERG	Human	9
Prostanoid EP_4	Human	-3
Purinergic P2X	Rat	4
Purinergic P2Y, Non-Selective	Rat	9
Rolipram	Rat	10
Serotonin (5-HT_{1A})	Human	-5
Serotonin (5-HT_{2B})	Human	10
Serotonin (5-HT_3)	Human	-9
Sigma σ_1	Human	11
Sodium Channel, Site 2	Rat	14
Tachykinin NK_1	Human	4
Thyroid Hormone	Rat	0
Transporter, Dopamine (DAT)	Human	32
Transporter, GABA	Rat	31
Transporter, Norepinephrine (NET)	Human	12
Transporter, Serotonin (SERT)	Human	-4

Procedures for Biological Experiments

Calcium mobilization assays:

All functional cell-based assays were performed in stable Chinese Hamster Ovary (CHO) cell lines constitutively expressing human M_5 or human M_1 receptors. For full muscarinic selectivity, CHO cells expressing human M_3, human M_2 plus G_{qi5} or human M_4 plus G_{qi5} were used. Cells were plated at 15,000 cells per 20 µL per well in black 384-well, TC-treated, clear-bottomed plates (Greiner) in Ham's F12 medium supplemented with 10% FBS and 20 mM HEPES. Cells were incubated overnight at 37 °C under 5% CO_2. The following day, the medium was removed and replaced with 1.2 µM Fluo-4 AM (Invitrogen) in assay buffer (Hank's Balanced Salt Solution supplemented with 20 mM HEPES and 2.5 mM Probenecid, pH 7.4) and the cells were incubated for 50 minutes at 37 °C under 5% CO_2. Dye was then removed and replaced with 20 µL of fresh assay buffer. Test compounds at a 10 mM concentration in DMSO were serially diluted in DMSO (either 1:3 or 1:5 dilution) to create a 10-point concentration series. The DMSO solutions were then diluted in assay buffer resulting in compound solutions at 2-times the final assay concentration with the highest assay concentration of 30 µM. The compound plate, cell plate, and plates containing EC_{20} and EC_{80} acetylcholine concentrations were placed in a Hamamatsu FDSS 6000 or 7000 kinetic imaging plate reader equipped to measure Ex_{480}/Em_{540} fluorescence. Data were collected at 1 frame per second. After 2 seconds of collecting baseline fluorescence, 20 µL of the compound solutions were added to the cell plate. This was followed by the addition of an EC_{20} concentration of acetylcholine at 142 seconds. At 267 seconds, an EC_{80} concentration of acetylcholine was added along with a maximally effective acetylcholine concentration in wells not containing a compound to allow data normalization. The fluorescence signal was collected for a total of 300 seconds. Compound concentration response curves (CRCs) were collected in triplicate across three separate plates. Data were imported and analyzed in Dotmatics Informatics software. by normalizing all data in the individual kinetic traces to the initial fluorescence read. The magnitude of each agonist addition was then determined and normalized to the average maximum response. This percent maximum response was plotted against log[compound] and fit to a four parameter logistical equation to determine log(IC_{50}). The IC_{50} determined using the EC_{80} of acetylcholine is the value reported. Compound CRC curves that did not plateau below 10% ACh_{max} were assigned a low potency value of >10 µM.

Drug Metabolism Methods:

In vitro

Plasma protein binding and Brain homogenate binding: Determination of fraction unbound (f_u) in plasma was conducted in vitro via equilibrium dialysis using HTDialysis (HTD) membrane plates. The top half of the plate was filled with 100 uL of Dubelco's Phosphate Buffered Saline, pH 7.4 (DPBS). Compounds were diluted into plasma from each species (5 µM final concentration), which was aliquoted in triplicate to the 'bottom half' of the prepared HTD plate wells. The HTD plate was sealed and incubated for 6 hours at 37 ˚C. Following incubation, each well (both top and bottom halves) were transferred (20 µL) to the corresponding wells of a 96-shallow-well (V-bottom) plate. The daughter plates were then matrix-matched (DPBS side wells received equal volume of plasma, and plasma side wells received equal volume of DPBS), and extraction solution (120 µL; acetonitrile containing 50 nM carbamazepine as IS) was added to all wells of both daughter plates to precipitate protein and extract test article. The plates were then sealed and centrifuged (3500 rcf) for 10 minutes at ambient temperature. Supernatant (60 µL) from each well of the daughter plates was then transferred to the corresponding wells of new daughter plates (96-shallow-well, V bottom) containing water (Milli-Q, 60 µL/well), and the plates were sealed in preparation for LC-MS/MS analysis (see LC-MS/MS analysis method below).

The unbound fraction (f_u) was calculated following the equation below, and mean values for each species were calculated from 3 replicates.

A similar approach was used to determine the degree of brain homogenate binding, which employed the same methodology and procedure with the following modifications: 1) a final compound concentration of 1 µM was used, 2) naïve rat brains were homogenized in DPBS (1:3 composition of brain: DPBS, w/w) using a Mini-Bead Beater™ machine in order to obtain brain homogenate, which was then treated in the same manner as the plasma samples in the previously described plasma protein binding assay. Fraction unbound for both plasma and brain samples was determined using Equation 4.

$$f_u = \frac{Conc_{buffer}}{Conc_{plasma}}$$

Equation 4 Determination of fraction unbound in plasma.

The diluted fraction unbound (f_u2) in brain was calculated in the same manner by using brain homogenate rather than plasma. Undiluted fraction unbound for the brain was calculated using Equation 5

$$f_u = \frac{1/4}{\left\{\left(\frac{1}{fu2}\right) - 1\right\} + 1/4}$$

Equation 5 Determination of fraction unbound in brain. F_u2 represents the diluted fraction unbound.

Intrinsic clearance: Human or rat hepatic microsomes (0.5 mg/mL) and 1 µM test compound were incubated in 100 mM potassium phosphate pH 7.4 buffer with 3 mM $MgCl_2$ at 37 °C with constant shaking. After a 5 min preincubation, the reaction was initiated by the addition of NADPH (1 mM). At selected time intervals (0, 3, 7, 15, 25, and 45 min), aliquots were taken and subsequently placed into a 96-well plate containing cold acetonitrile with internal standard (50 ng/mL carbamazepine). Plates were then centrifuged at 3000 rcf (4 °C) for 10 min, and the supernatant was transferred to a separate 96-well plate and diluted 1:1 with water for LC/MS/MS analysis. The *in vitro* half-life ($_{1/2}$, min, Eq. 1), intrinsic clearance (CL_{int}, mL/min/kg, Eq. 2), and subsequent predicted hepatic clearance (CL_{hep}, mL/min/kg, Eq. 3) was determined employing the following equations:

(1) $T_{1/2} = \frac{Ln(2)}{K}$

where k represents the slope from linear regression analysis of the natural log percent remaining of a test compound as a function of incubation time

(2) $CL_{int} = \frac{0.693}{in\ vitro T_{1/2}}\ x\ \frac{mL\ incubation}{mg\ microsomes}\ x\ \frac{45\ mg\ microsomes}{gram\ liver}\ x\ \frac{20^a\ gram\ liver}{kg\ body\ wt}$

[a]scale-up factors: of 20 (human) or 45 (rat)

(3) $CL_{hep} = \frac{Q_h \cdot CL_{int}}{Q_h + CL_{int}}$

where Q_h (hepatic blood flow, mL/min/kg) is 21 (human) or 70 (rat).

LC/MS/MS Bioanalysis of Samples from Plasma Protein Binding and Intrinsic Clearance Assays:

Samples were analyzed on a Thermo Electron TSQ Quantum Ultra triple quad mass spectrometer (San Jose, CA) via electrospray ionization (ESI) with two Themo Electron Accella pumps (San Jose, CA), and a Leap Technologies CTC PAL autosampler (Carrboro, NC). Analytes were separated by gradient elution on a dual column system with two Thermo Hypersil Gold (2.1 x 30 mm, 1.9 µm) columns (San Jose, CA) thermostated at 40 °C. HPLC mobile phase A was 0.1% formic acid in water and mobile phase B was 0.1% formic acid in acetonitrile. The gradient started at 10% B after a 0.2 min hold and was linearly increased to 95% B over 0.8 min; hold at 95% B for 0.2 min; returned to 10% B in 0.1 min. The total run time was 1.3 min and the HPLC flow rate was 0.8 mL/min. While pump 1 ran the gradient method, pump 2 equilibrated the alternate column isocratically at 10% B. Compound optimization, data collection, and processing was performed using Thermo Electron's QuickQuan software (v2.3) and Xcalibur (v2.0.7 SP1).

***In vivo* DMPK experimental:**

Determination of brain to plasma ratio:

Animal care and use

All animal study procedures were approved by the Institutional Animal Care and Use Committee and were conducted in accordance with the National Institutes of Health regulations of animal care covered in Principles of Laboratory Animal Care (National Institutes of Health). All rats were fasted overnight prior to testing.

In-life phase

For determination of the brain over plasma ratio (K_p), compounds were formulated in 8% ethanol, 32% PEG400 and 60% DMSO (*v/v/v*) and administered as a single 0.2 mg/kg IV dose (1 mL/kg) to male, Sprague Dawley rats (n = 1) via injection into a surgically-implanted jugular vein catheter. At 15 min post dosing, blood sample was collected into chilled, K_2EDTA anticoagulant-fortified tube and immediately placed on wet ice. The blood sample was then centrifuged (1700 rcf, 5 minutes, 4 ˚C) to obtain plasma sample. At the same post-administration time point, whole

brain sample was obtained by rapid dissection, rinsed with PBS, and immediately frozen in individual tissue collection box (dry ice). All brain and plasma samples were stored at -80 ˚C until analysis by LC-MS/MS.

Sample Analysis: Concentrations in plasma and brain homogenates were quantified by liquid chromatography tandem mass spectrometry (LC-MS/MS). Whole brains were homogenized in 3 mL of 70:30 IPA:water in a mini bead beater for 3 min, and centrifuged at 3,500 g for 5 min. 5 uL of the supernatant was diluted in 15 uL of blank plasma for quantification of the analytes. Plasma samples were centrifuged at 3,500 g for 5 min. A standard curve was generated by diluting the analytes DMSO stocks with blank plasma to obtain a final concentration of 10,000 ng/ml followed by a serial dilution down to 0.5 ng/ml. Quality controls were generated by a serial dilution of the 5,000 ng/ml standard curve solution in blank plasma to obtain 3 concentrations of 500, 50, and 5 ng/ml. 20 uL of brain diluted in plasma, plasma, blank plasma, standard curve and QC samples were loaded in a V-bottom 96-well plate. 120 uL of acetonitrile containing 0.05 uM carbamazepine (internal standard) was added to each well and the plate was centrifuged at 3,500 g for 5 min. 60 uL of the supernatant of each well (protein free) was transferred to a new 96-well plate containing 60 uL of water. The plates were sealed for analysis by LC-MS/MS.

Plasma and brain tissue samples originating from *in vivo* studies were analyzed by electrospray ionization using an AB Sciex Q-TRAP 5500 (Foster City, CA) that was coupled to a Shimadzu LC-20AD pump (Columbia, MD) and a Leap Technologies CTC PAL auto-sampler (Carrboro, NC). Analytes were separated by gradient elution using a C18 column (3 x 50 mm, 3 mm; Fortis Technologies Ltd, Cheshire, UK) that was thermostated at 40 °C. HPLC mobile phase A was 0.1% formic acid in water (pH unadjusted); mobile phase B was 0.1% formic acid in acetonitrile (pH unadjusted). A 10% B gradient was held for 0.2 min and was linearly increased to 90% B over 0.8 min, with an isocratic hold for 0.5 min, before transitioning to 10% B over 0.05 min. The column was re-equilibrated (1 min) before the next sample injection. The total run time was 2.55 min, and the HPLC flow rate was 0.5 ml/min. The source temperature was set at 500 °C, and mass spectral analyses were performed using a Turbo-Ion spray source in positive ionization mode (5.0-kV spray voltage) and using multiple-reaction monitoring of transitions specific for the analytes. All data were analyzed using AB Sciex Analyst 1.5.1 software.

Brain plasma concentration ratio (K_p) was calculated by dividing brain concentration by plasma concentration for each animal. Unbound brain to unbound plasma concentration ratio ($K_{p,uu}$) is calculated using the following formula: $K_{p,uu}$ = (Brain ng/g x brain fu)/(plasma ng/ml x plasma fu).

Pharmacokinetic profiles in rats following oral single escalating doses

Single escalating oral dosing in Sprague-Dawley rats was performed at Frontage Laboratories according to their non-GLP Standard Operating Procedure and IACUC protocols. In short, compounds were formulated in 10% Tween 80 in water and dosed at 10 mg/kg. At different times, arterial blood was collected from a femoral artery catheter, and compound concentration was determined in plasma by LC-MS/MS following their non-GLP protocol. PK parameters were determined using Phoenix WinNonlin software (version 6.3).

In-vitro determination of blood-brain barrier penetration potential

Blood-brain barrier penetration was determined using MDR1-MDCK cell monolayers by Absorption Systems, following their protocol. In short, compounds were incubated at 5 mM final concentration on one side of the cell monolayer for 2 hours. Compounds concentration on either side of the monolayer was determined by LC-MS/MS and apparent permeability and efflux ratio were determined as described in Wang, Q. et al.[1]

Behavioral Manifestations of Seizure Activity

To evaluate induction of behavioral manifestation of seizure activity, C57Bl/6 mice received administration of vehicle or 100 mg/kg M_1 PAM **5**. Compound was formulated in 30% Captisol (pH 7.0) at a concentration of 10 mg/mL and injected i.p. at 10 ml/kg ($n = 4$). Animals were monitored continuously and scored for behavioral manifestations of seizure activity at 5, 15, 30 min, and 1 h. Behavioral manifestations of seizures were scored using a modified Racine scoring system. Briefly, a score of 0 represents no behavior alterations; score 1, immobility, mouth and facial movements, or facial clonus; score 2, head nodding, tail extension; score 3, forelimb clonus, repetitive movements; score 4, rearing and tonic clonic seizure; and score 5, continuous rearing and falling, severe generalized tonic clonic seizure.

Novel Object Recognition Task

Rats were habituated for 10 min for 2 consecutive days in an empty novel object recognition (NOR) arena consisting of dark-colored plexiglass box (40 × 64 × 33 cm^3). On day 3 rats were administered vehicle, M_1 PAM **3** (1-10 mg/kg, p.o.., 10 mL/kg, n = 13-18), or M_1 PAM **5** (0.3-3 mg/kg, p.o.., 10 mL/kg, n = 16-18) and returned to their home cage for 60 min (M_1 PAM **5**) or 2 hr (M_1 PAM **3**. Rats were then placed in the NOR arena containing two identical objects for 10 min. Following the exposure period, rats were placed back into their home cages for 24 h. The rats were then returned to the arena in which one of the previously exposed (familiar) objects was replaced by a novel object and were video recorded for 5 min while they explored the two objects. Time spent exploring each object was scored by an observer blinded to the experimental conditions and the recognition index was calculated as [(time spent exploring novel object) – (time spent exploring familiar object)]/total time exploring objects.

General Methods

All NMR spectra were recorded on a 400 MHz AMX Bruker NMR spectrometer. ^{1}H and ^{13}C chemical shifts are reported in δ values in ppm downfield with the deuterated solvent as the internal standard. Data are reported as follows: chemical shift, multiplicity (s = singlet, d = doublet, t = triplet, q = quartet, b = broad, m = multiplet), integration, coupling constant (Hz). Low resolution mass spectra were obtained on an Agilent 6120/6150 or Waters QDa (Performance) SQ MS with ESI source. *Method A (Agilent 6120/6150):* MS parameters were as follows: fragmentor: 70, capillary voltage: 3000 V, nebulizer pressure: 30 psig, drying gas flow: 13 L/min, drying gas temperature: 350 ºC. Samples were introduced via an Agilent 1290 UHPLC comprised of a G4220A binary pump, G4226A ALS, G1316C TCC, and G4212A DAD with ULD flow cell. UV absorption was generally observed at 215 nm and 254 nm with a 4 nm bandwidth. Column: Waters Acquity BEH C18, 1.0 x 50 mm, 1.7 um. Gradient conditions: 5% to 95% CH_3CN in H_2O (0.1% TFA) over 1.4 min, hold at 95% CH_3CN for 0.1 min, 0.5 mL/min, 55 ºC. *Method B (Agilent 6120/6150):* MS parameters were as follows: fragmentor: 100, capillary voltage: 3000 V, nebulizer pressure: 40 psig, drying gas flow: 11 L/min, drying gas temperature: 350 ºC. Samples were introduced via an Agilent 1200 HPLC comprised of a degasser, G1312A binary pump, G1367B HP-ALS, G1316A TCC, G1315D DAD, and a Varian 380 ELSD (if applicable). UV absorption was generally observed at 215 nm and 254 nm with a 4 nm bandwidth. Column: Thermo Accucore C18, 2.1 x 30 mm, 2.6 um. Gradient conditions: 7% to 95% CH_3CN in H_2O (0.1% TFA) over 1.6

min, hold at 95% CH_3CN for 0.35 min, 1.5 mL/min, 45 ºC. *Method C (Waters QDa (Performance) SQ MS)*: MS parameters were as follows: cone voltage: 15 V, capillary voltage: 0.8 kV, probe temperature: 600° C. Samples were introduced via an Acquity I-Class PLUS UPLC comprised of a BSM, FL-SM, CH-A, and PDA. UV absorption was generally observed at 215 nm and 254 nm; 4 nm bandwidth. Column: Phenomenex EVO C18, 1.0 x 50 mm, 1.7 um. Column temperature: 55° C. Flow rate: 0.4 mL/min. Default gradient: 5% to 95% CH_3CN (0.05% TFA) in H_2O (0.05% TFA) over 1.4 min (curve 6), hold at 95% CH_3CN for 0.1 min. "Polar" (2% to 70% CH_3CN (0.05% TFA) in H_2O (0.05% TFA) over 0.8 min (curve 6), transition to 95% CH_3CN over 0.1 min (curve 6), hold at 95% CH_3CN for 0.6 min.) and "Non-Polar" (40% to 95% CH_3CN (0.05% TFA) in H_2O (0.05% TFA) over 1.4 min (curve 6), hold at 95% CH_3CN for 0.1 min.) gradients were also available. *Method D (Waters QDa (Performance) SQ MS):* MS parameters were as follows: cone voltage: 15 V, capillary voltage: 0.8 kV, probe temperature: 600° C. Samples were introduced via an Acquity I-Class PLUS UPLC comprised of a BSM, FL-SM, CH-A, and PDA. UV absorption was generally observed at 215 nm and 254 nm with a 4 nm bandwidth. Column: Phenomenex EVO C18, 1.0 x 50 mm, 1.7 um. Column temperature: 55° C. Flow rate: 0.4 mL/min. Default gradient: 5% to 95% CH_3CN in H_2O (5 mM NH_4HCO_3) over 1.4 min (curve 6), hold at 95% CH_3CN for 0.1 min. "Polar" (2% to 70% CH_3CN in H_2O (5 mM NH_4HCO_3) over 0.8 min (curve 6), transition to 95% CH_3CN over 0.1 min (curve 6), hold at 95% CH_3CN for 0.6 min.) and "Non-Polar" (40% to 95% CH_3CN in H_2O (5 mM NH_4HCO_3) over 1.4 min (curve 6), hold at 95% CH_3CN for 0.1 min.) gradients were also available. High resolution mass spectra were obtained on an Agilent 6540 UHD Q-TOF with ESI source. MS parameters were as follows: fragmentor: 150, capillary voltage: 3500 V, nebulizer pressure: 60 psig, drying gas flow: 13 L/min, drying gas temperature: 275 ºC. Samples were introduced via an Agilent 1200 UHPLC comprised of a G4220A binary pump, G4226A 3 ALS, G1316C TCC, and G4212A DAD with ULD flow cell. UV absorption was observed at 215 nm and 254 nm with a 4 nm bandwidth. Column: Agilent Zorbax Extend C18, 1.8 μm, 2.1 x 50 mm. Gradient conditions: 5% to 95% CH_3CN in H_2O (0.1% formic acid) over 1 min, hold at 95% CH_3CN for 0.1 min, 0.5 mL/min, 40 ºC. For compounds that were purified on a Gilson preparative reversed-phase HPLC, the system comprised of a 333 aqueous pump with solvent selection valve, 334 organic pump, GX 271 or GX-281 liquid hander, two column switching valves, and a 155 UV detector. UV wavelength for fraction collection was user-defined, with absorbance at 254 nm always monitored. Method 1: Phenomenex Axia-packed

Luna C18, 30 x 50 mm, 5 µm column. Mobile phase: CH_3CN in H_2O (0.1% TFA). Gradient conditions: 0.75 min equilibration, followed by user defined gradient (starting organic percentage, ending organic percentage, duration), hold at 95% CH_3CN in H_2O (0.1% TFA) for 1 min, 50 mL/min, 23 ºC. Method 2: Phenomenex Axia packed Gemini C18, 50 x 250 mm, 10 um column. Mobile phase: CH_3CN in H_2O (0.1% TFA). Gradient conditions: 7 min equilibration, followed by user defined gradient (starting organic percentage, ending organic percentage, duration), hold at 95% CH_3CN in H_2O (0.1% TFA) for 7 min, 120 mL/min, 23 ºC. Solvents for extraction, washing and chromatography were HPLC grade. All compounds are >95% purity by HPLC.

Synthetic Procedures and Spectra

Scheme 1. Synthesis of VU6030095 (**3**).[a]

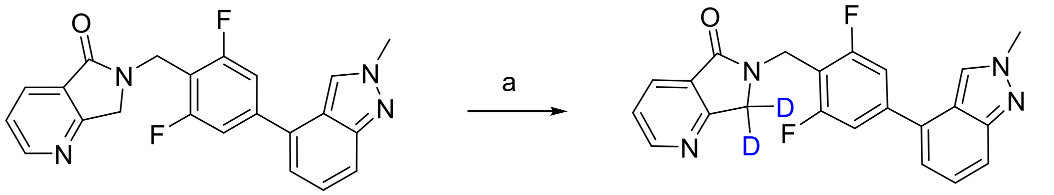

1 **3**

[a] Reagents and conditions: (a) NaOD (40 wt% in D_2O), THF/D_2O, 35 °C, 91%.

Preparation of 6-(2,6-difluoro-4-(2-methyl-2*H*-indazol-4-yl)benzyl)-6,7-dihydro-5*H*-pyrrolo[3,4-*b*]pyridin-5-one-7,7-*d*$_2$ (VU6030095 or **3**)

To a solution of 6-[[2,6-difluoro-4-(2-methylindazol-4-yl)phenyl]methyl]-7*H*-pyrrolo[3,4-*b*]pyridin-5-one (390.39 mg, 1.0 mmol, 1 eq.) in THF (11.42 mL, 0.09 M) and deuterium oxide (2.44 mL, 150.0 mmol, 150 eq.) was added a solution of sodium deuteroxide (40% in D_2O, 17 µL, 0.25 mmol, 0.25 eq.). The resulting mixture was stirred at 35 °C for 16 h. After cooling to rt, the reaction mixture was diluted with EtOAc. Layers were separated. Aqueous layer was extracted with EtOAc (3x). Combined extracts were washed with brine, dried ($MgSO_4$) filtered and concentrated. The crude material was purified by reverse-phase chromatography to afford the title compound (356 mg, 91% yield). ^{1}H NMR (400 MHz, DMSO-d_6) δ 8.75 (dd, J = 5.0, 1.6 Hz, 1H), 8.62 (s, 1H), 8.12 (dd, J = 7.7, 1.6 Hz, 1H), 7.66 (d, J = 8.5 Hz, 1H), 7.57 – 7.44 (m, 3H), 7.33 (dd, J = 8.5, 7.0 Hz, 1H), 7.26 (d, J = 6.9 Hz, 1H), 4.91 (s, 2H), 4.18 (s, 3H); ^{13}C NMR (101 MHz,

DMSO-d_6) δ 165.92, 162.95 (d, $J = 9.3$ Hz), 162.57, 160.49 (d, $J = 9.2$ Hz), 153.09, 149.03, 142.70 (t, $J = 10.1$ Hz), 131.93, 131.30 (t, $J = 2.4$ Hz), 125.95, 125.84, 124.89, 123.97, 121.03, 120.09, 117.93, 111.92 – 111.00 (m, 3C), 51.41 – 50.05 (m, CD_2), 40.56, 33.85 (t, $J = 3.4$ Hz); HRMS (ESI/Q-TOF) m/z: $[M+H]^+$ calc'd for $C_{22}H_{14}D_2F_2N_4O$ 393.190, found = 393.1490.

Scheme 2. Synthesis of VU6045422 (**5**).[a]

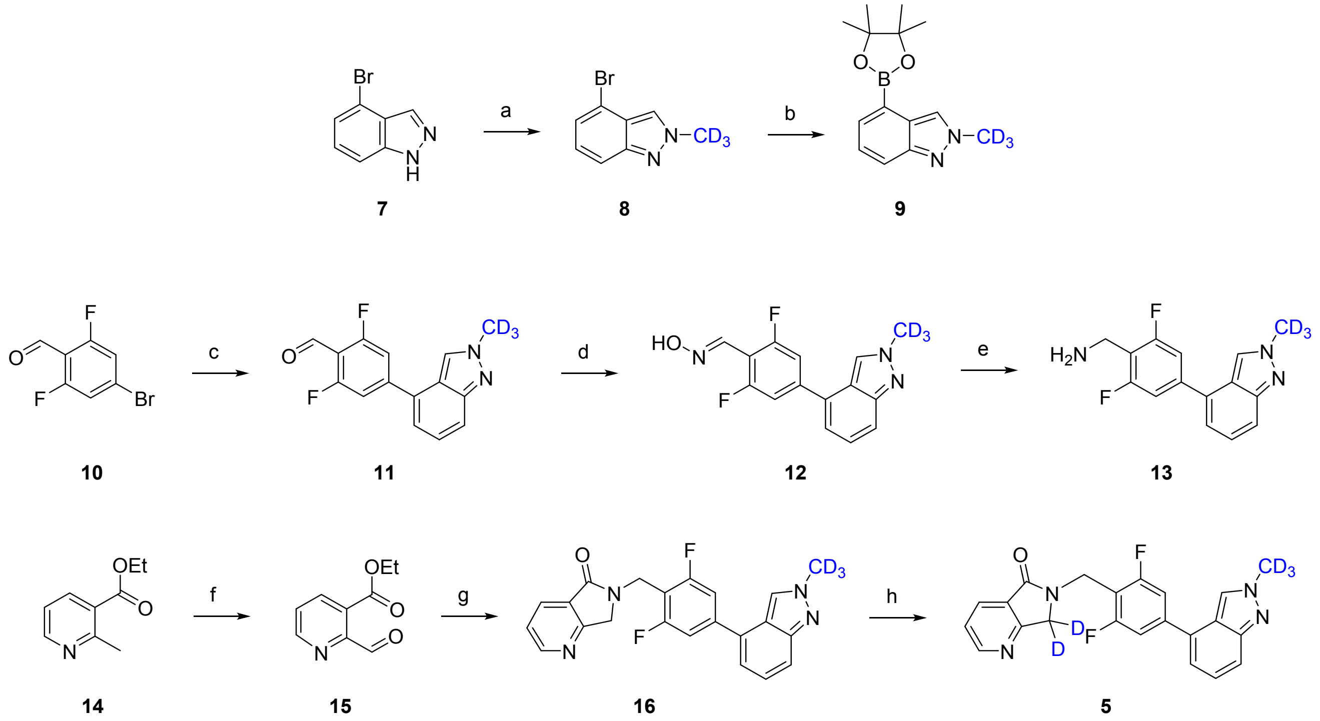

[a] Reagents and conditions: (a) ICD_3, Cs_2CO_3, MeCN, rt, 33%; (b) *bis*-pinacoborane, $PdCl_2(dppf){\cdot}CH_2Cl_2$, KOAc, 1,4-dioxane, 100 °C, 72%; (c) **9**, $PdCl_2(dppf){\cdot}CH_2Cl_2$, Cs_2CO_3, 1,4-dioxane/H_2O, 100 °C, 96%; (d) NH_2OH-HCl, NaOAc, EtOH, 98%; (e) Zn/HOAc, rt, 96%; (f) SeO_2, 1,4-dioxane, 85 °C, 45%; (g) **13**, STAB, DCE, 57%; (h) NaOD (40 wt % in D_2O, THF/D_2O), 45 °C, 83%.

Step a: Preparation of 4-Bromo-2-(methyl-*d*$_3$)-2*H*-indazole (8)

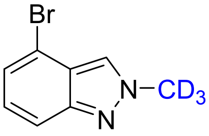

8

In a 2L RBF, 4-bromo-*1H*-indazole (59.11 g, 300 mmol, 1.0 eq.), cesium carbonate (196.7 g, 600 mmol, 2.0 eq.) and anhydrous MeCN (1500 mL, 0.2 M) was combined. Iodomethane-d_3 (37.35 mL, 600 mmol, 2.0 eq.) was added. After 16 h at rt with vigorous stirring, the reaction mixture

was filtered to remove insoluble salts. The filtrate was concentrated under reduced pressure. The resulting oil was partitioned between deionized H_2O (400 mL) and EtOAc (400 mL). The layers were separated. The aqueous layer was extracted with EtOAc (3 x 400 mL). The combined organic extracts were washed with brine, dried over Na_2SO_4, filtered and concentrated. The crude material was purified using normal phase chromatography on silica gel (0-30% EtOAc/hexanes) to provide two separated isomers. 4-Bromo-2-(methyl-d_3)-2*H*-indazole (8 - desired isomer 21.34 g, 33% yield). ^{1}H NMR (400 MHz, DMSO-d_6) δ 8.39 (d, J = 0.9 Hz, 1H), 7.62 (dd, J = 8.5, 1.1 Hz, 1H), 7.26 (d, J = 7.1 Hz, 1H), 7.16 (dd, J = 8.6, 7.2 Hz, 1H); ^{13}C NMR (101 MHz, $CDCl_3$) δ 148.94, 126.75, 124.76, 124.32, 124.12, 116.52, 112.80, 39.88 (dt, J = 42.9, 21.5 Hz, CD_3); HRMS (ESI/Q-TOF) m/z: $[M+H]^+$ calc'd for $C_8H_4D_3BrN_2$ 214.0054, found = 214.0055. 4-Bromo-1-(methyl-d_3)-1*H*-indazole (undesired isomer, 35.35g, 55% yield): ^{1}H NMR (400 MHz, DMSO-d_6) δ 8.01 (d, J = 1.0 Hz, 1H), 7.70 (dt, J = 8.0, 1.1 Hz, 1H), 7.37 (dd, J = 7.4, 1.1 Hz, 1H), 7.34 (d, J = 7.8 Hz, 1H).

Step b: Preparation of 2-(methyl-*d₃*)-4-(4,4,5,5-tetramethyl-1,3,2-dioxaborolan-2-yl)-2*H*-indazole (9)

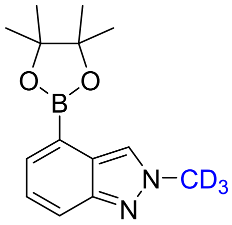

9

In an oven-dried 1L RBF equipped with a Findenser, 4-bromo-2-(methyl-d$_3$)-2*H*-indazole (20.0 g, 93.42 mmol, 1.0 eq.), potassium acetate (27.51 g, 280.27 mmol, 3.0 eq.), [1,1'-bis(diphenylphosphino)ferrocene]dichloropalladium(II) complex with dichloromethane (5.74 g, 7.01 mmol, 0.075 eq.) and *bis*-pinacolborane (35.59 g, 140.13 mmol, 1.5 eq.) were combined. Anhydrous 1,4-dioxane (467 mL, 0.2 M) was added. The reaction mixture was evacuated and purged with nitrogen (3x) and allowed to stir at 90 °C. After 16 h, the reaction mixture was filtered through a pad of Celite® which was rinsed thoroughly with EtOAc. Filtrate was concentrated under reduced pressure. The crude material was purified using normal phase chromatography on silica gel (0-100% EtOAc/hexanes) provided the title compound as an off-white powder (17.61 g, 72% yield). ^{1}H NMR (400 MHz, $CDCl_3$) δ 8.25 (d, J = 1.0 Hz, 1H s, 1H), 7.81 (dt, J = 8.7, 1.0 Hz,

1H), 7.63 (dd, J = 6.6, 0.9 Hz, 1H), 7.30 (dd, J = 8.7, 6.6 Hz, 1H), 1.38 (s, 12H); ^{13}C NMR (101 MHz, $CDCl_3$) δ 147.81, 130.94, 126.30, 126.05, 125.39, 120.25, 83.96, 83.21 (2C), 39.58 (dt, J = 42.9, 21.3 Hz, CD_3), 25.13 (4C); Note: peaks at δ 83.21 and 24.73 represent unreacted bis(pincolato)diboron; HRMS (ESI/Q-TOF) m/z: $[M+H]^+$ calc'd for $C_{14}H_{16}D_3BN_2O_2$ 262.1803, found = 262.1804.

Step c: Preparation of 2,6-difluoro-4-(2-(methyl-*d₃*)-2*H*-indazol-4-yl)benzaldehyde (11)

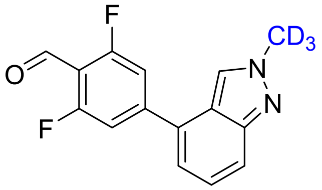

11

In an oven-dried 1L RBF equipped with a Findenser, 4-bromo-2,6-difluorobenzaldehyde (15.47 g, 70 mmol, 1.0 eq.), 2-(methyl-d_3)-4-(4,4,5,5-tetramethyl-1,3,2-dioxaborolan-2-yl)-2*H*-indazole (21.94 g, 84 mmol, 1.2 eq.), [1,1'-bis(diphenylphosphino)ferrocene]dichloropalladium(II), complex with dichloromethane (4.30 g, 5.25 mmol, 0.075 eq.), and cesium carbonate (45.62 g, 140 mmol, 2.0 eq.) were combined. Anhydrous 1,4-dioxane (300 mL) and H_2O (50 mL) were added. The reaction mixture was evacuated and purged with N_2 (3x) and allowed to stir at 100 °C. After 1 h, the reaction mixture was diluted with 10% MeOH/DCM solution and water. The layers were separated, and the aqueous layer was extracted with 10% MeOH/DCM (3x). The combined extracts were dried over $MgSO_4$, filtered and concentrated. The crude material was suspended in EtOAc. The precipitate was collected using vacuum filtration, washed with EtOAc and dried under vacuum to provide the tittle compound, which was used in the subsequence step without further purification (14.1 g). ^{1}H NMR (400 MHz, $CDCl_3$) δ 10.38 (s, 1H), 8.07 (s, 1H), 7.80 (d, J = 8.7 Hz, 1H), 7.40 (dd, J = 8.7, 7.0 Hz, 1H), 7.38 – 7.26 (m, 2H), 7.23 (d, J = 7.0 Hz, 1H); ^{13}C NMR (101 MHz, $CDCl_3$) δ 184.29 (t, J = 4.3 Hz), 164.84 (d, J = 6.7 Hz), 162.22 (d, J = 6.8 Hz), 149.50, 149.34 (t, J = 11.2 Hz), 130.65 (t, J = 2.3 Hz), 126.09, 123.11, 122.14, 120.35, 119.22, 112.93 (t, J = 11.1 Hz), 112.15 – 111.45 (m, 2C), 40.13 (dt, J = 43.1, 21.5 Hz, CD_3); HRMS (ESI/Q-TOF) m/z: $[M+H]^+$ calc'd for $C_{15}H_7D_3F_2N_2O$ 276.1022, found = 276.1023.

Step d: Preparation of (*E/Z*)-2,6-Difluoro-4-(2-(methyl-*d₃*)-2*H*-indazol-4-yl)benzaldehyde oxime (12)

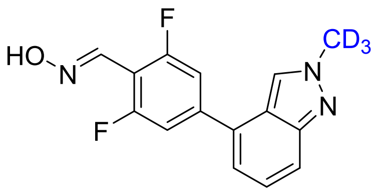

12

In a 1L RBF, to a suspension of 2,6-difluoro-4-(2-(methyl-d$_3$)-2*H*-indazol-4-yl)benzaldehyde (21.8 g, 79.2 mmol, 1.0 eq.,) in ethanol (400 mL, 0.2 M) was added sodium acetate (12.99 g, 158.4 mmol, 2.0 eq.) and hydroxylamine hydrochloride (11.01 g, 158.4 mmol, 2.0 eq.). After 16 h at rt, the reaction mixture was diluted with EtOAc (~1.7 L), washed with H_2O and brine. The organic layer was dried over $MgSO_4$, filtered and concentrated to provide the title compound, which was carried forward without further purification (31.95 g). Note: The material is insoluble in DCM. ^{1}H NMR (400 MHz, DMSO-*d*$_6$) δ 8.66 (d, *J* = 1.0 Hz, 1H), 8.21 (s, 1H), 7.68 (dt, *J* = 7.9, 1.3 Hz, 1H), 7.59 – 7.46 (m, 2H), 7.41 – 7.26 (m, 2H), OH proton is not observable.

Step e: Preparation of (2,6-Difluoro-4-(2-(methyl-*d*$_3$)-2*H*-indazol-4-yl)phenyl)methanamine (13)

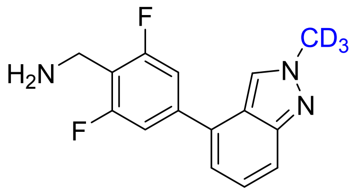

13

In a 1L RBF, (E/Z)-2,6-difluoro-4-(2-(methyl-d$_3$)-2*H*-indazol-4-yl)benzaldehyde oxime (31.95 g, 79.19 mmol, 1.0), acetic acid (264 mL, 0.3 M) and zinc powder (25.89 g, 395.96 mmol, 5.0 eq.) were combined. The mixture was sonicated at rt for 2 h. Additional zinc powder (3.8 g, 58.12 mmol) was added twice within 16 h. Upon completion by LCMS, the mixture was filtered through a pad of Celite®. The filtrate was concentrated under reduced pressure to provide the title compound as a viscous oil which was carried forward without further purification (43.6 g). ^{1}H NMR (400 MHz, DMSO-*d*$_6$) δ 8.60 (d, *J* = 1.0 Hz, 1H), 7.66 (dt, *J* = 8.5, 1.1 Hz, 1H), 7 52 – 7.39 (m, 2H), 7.33 (dd, *J* = 8.3, 7.0 Hz, 1H), 7.26 (dd, *J* = 6.9, 0.9 Hz, 1H), 3.94 (s, 2H), NH proton is not observable, 1.85 (leftover acetic acid); ^{13}C NMR (101 MHz, DMSO-*d*$_6$) δ 162.46 (d, *J* = 8.7 Hz), 159.99 (d, *J* = 8.6 Hz), 148.56, 143.53 (t, *J* = 10.3 Hz), 130.44 (d, *J* = 2.7 Hz), 125.51, 124.27, 120.91, 119.42, 117.83, 111.25 – 110.30 (m, 2C), 108.78 (t, *J* = 19.9 Hz), 66.36, 30.19, CD_3 is

hidden under DMSO-d_6; HRMS (ESI/Q-TOF) m/z: $[M+H]^+$ calc'd for $C_{15}H_{10}D_3F_2N_3$ 277.1339, found = 277.1341.

Step f: Preparation of ethyl 2-formylnicotinate (15)

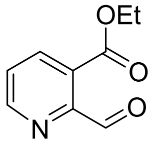

15

Reference: PCT Int. Appl., 2002012442, 14 Feb 2002, example 21f. In an oven-dried 1L RBF equipped with a Findenser, to a solution of ethyl 2-methylnicotinate (23.11 mL, 150 mmol, 1.0 eq.) in 1,4-dioxane (375 mL, 0.4M) at 85 °C was added activated selenium dioxide (24.97 g, 225 mmol, 1.5 eq.). The reaction mixture was refluxed under nitrogen. Additional selenium dioxide (24.97 g, 225 mmol, 1.5 eq.) was added after 4 h and 8 h until completion by LCMS. After cooling to rt, the reaction mixture was filtered. The filtrate was concentrated under reduced pressured. Purification using normal phase chromatography on silica gel (0-100% EtOAc/hexanes) to provide the title compound as an orange oil (12.2 g, 45% yield). ^1H NMR (400 MHz, DMSO-d_6) δ 10.16 (s, 1H), 8.92 (dd, J = 4.8, 1.0 Hz, 1H), 8.20 (ddd, J = 7.9, 1.6, 0.5 Hz, 1H), 7.78 (dd, J = 7.8, 4.7 Hz, 1H), 4.35 (q, J = 7.1 Hz, 2H), 1.31 (t, J = 7.1 Hz, 3H); ES-MS $[M+H]^+$ = 180.2; ^{13}C NMR (101 MHz, DMSO-d_6) δ 192.36, 166.05, 151.76, 150.78, 137.03, 128.40, 127.24, 61.85, 13.78; HRMS (ESI/Q-TOF) m/z: $[M+H]^+$ calc'd for $C_9H_9NO_3$ 180.0655, found = 180.0654.

Step g: Preparation of 6-(2,6-difluoro-4-(2-(methyl-*d*$_3$)-2*H*-indazol-4-yl)benzyl)-6,7-dihydro-5*H*-pyrrolo[3,4-*b*]pyridin-5-one (16)

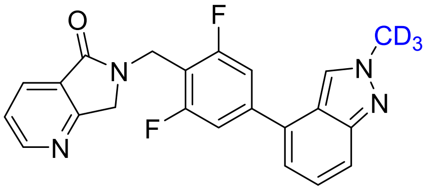

16

In an oven-dried 1L RBF, to a solution of (2,6-difluoro-4-(2-(methyl-d_3)-2*H*-indazol-4-yl)phenyl)methanamine (43.95 g, 50% wt, 79.8 mmol, 1.3 eq.) in DCE (270.5 mL, 0.2 M) was added a solution of methyl 2-formylnicotinate (11.0 g, 61.4 mmol, 1.0 eq.) in DCE (36.5 mL). The

resulting mixture was stirred at rt. After 30 min, sodium triacetoxyborohydride (19.52 g, 92.1 mmol, 1.5 eq) was added in portions. After 16 h at rt, the reaction mixture was diluted with DCM (~ 1.7 L) and washed with a sat. soln. of $NaHCO_3$ and brine. The organic layer was dried over Na_2SO_4, filtered and concentrated. The crude material was suspended in MeCN (~150 mL) and filtered using vacuum filtration. The resulting precipitate was further purified using normal phase chromatography on silica gel (0 – 10% MeOH/DCM) to provide the title compound as an off-white powder (13.78 g, 57% yield). ^{1}H NMR (400 MHz, DMSO-d_6) δ 8.75 (dd, J = 4.9, 1.6 Hz, 1H), 8.62 (d, J = 1.0 Hz, 1H), 8.12 (dd, J = 7.7, 1.6 Hz, 1H), 7.66 (dt, J = 8.5, 1.0 Hz, 1H), 7.57 – 7.43 (m, 3H), 7.33 (dd, J = 8.5, 7.0 Hz, 1H), 7.26 (dd, J = 7.0, 0.9 Hz, 1H), 4.92 (s, 2H), 4.50 (s, 2H); ^{13}C NMR (101 MHz, $CDCl_3$) δ 166.33, 163.19 (d, J = 8.8 Hz), 162.10, 160.71 (d, J = 8.8 Hz), 152.58, 149.53, 143.46 (t, J = 10.0 Hz), 132.25, 131.49 (t, J = 2.4 Hz), 126.35, 126.16, 123.41, 123.30, 121.36, 120.74, 118.01, 111.47 – 110.46 (m, 3C), 51.24, 40.01 (dt, J = 42.1, 21.6 Hz, CD_3), 33.82 (t, J = 3.5 Hz); HRMS (ESI/Q-TOF) m/z: $[M+H]^+$ calc'd for $C_{22}H_{13}D_3F_2N_4O$ 394.1553, found = 394.1552.

Step h: Preparation of 6-(2,6-difluoro-4-(2-(methyl-*d*$_3$)-2*H*-indazol-4-yl)benzyl)-6,7-dihydro-5*H*-pyrrolo[3,4-*b*]pyridin-5-one-7,7-*d*$_2$ (5, VU6045422)

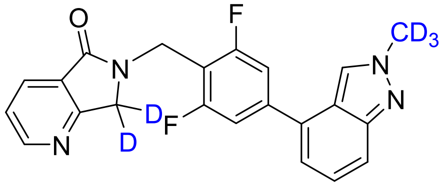

5 (VU6045422)

In an oven-dried 1L RBL equipped with a Findenser, 6-(2,6-difluoro-4-(2-(methyl-*d*$_3$)-2*H*-indazol-4-yl)benzyl)-6,7-dihydro-5*H*-pyrrolo[3,4-*b*]pyridin-5-one (13.78 g, 35.03 mmol, 1.0 eq.) was dissolved in THF (400 mL) and D_2O (86 mL). A solution of sodium deuteroxide (40 wt % in D_2O, 0.59 mL, 8.76 mmol, 0.25 eq.) was added. The reaction mixture was stirred at 35 °C. After 16 h, the reaction mixture was diluted with EtOAc (~1.2 L). Layers were separated. The aqueous layer was extracted with EtOAc (3x). Combined organic layers were washed with brine, dried over Na_2SO_4, filtered and concentrated. The crude material was suspended in MeCN (~150 mL). The precipitate was collected using vacuum filtration and subjected to a 2nd deuteration process using the condition above with 0.1 eq. of NaOD solution (0.24 mL). After work-up, the crude material

was purified using normal phase chromatography on silica gel (0-10% MeOH/DCM) to provide the title compound as a white powder (11.45 g, 83% yield). ^1H NMR (400 MHz, DMSO-d_6) δ 8.75 (dd, J = 4.9, 1.6 Hz, 1H), 8.62 (d, J = 1.0 Hz, 1H), 8.12 (dd, J = 7.7, 1.6 Hz, 1H), 7.66 (d, J = 8.5 Hz, 1H), 7.57 – 7.44 (m, 3H), 7.33 (dd, J = 8.5, 7.0 Hz, 1H), 7.27 (dd, J = 7.0, 0.9 Hz, 1H), 4.91 (s, 2H); ^{13}C NMR (101 MHz, $CDCl_3$) δ 166.40, 163.20 (d, J = 8.8 Hz), 162.07, 160.71 (d, J = 8.8 Hz), 152.64, 149.56, 143.47 (t, J = 10.1 Hz), 132.19, 131.49 (t, J = 2.4 Hz), 126.40, 126.14, 123.42, 123.28, 121.36, 120.75, 118.03, 111.49 – 110.57 (m, 3C), 51.67 – 49.52 (m, CD_2), 40.88 – 38.79 (m, CD_3), 33.81 (t, J = 3.5 Hz); HRMS (ESI/Q-TOF) m/z: $[M+H]^+$ calc'd for $C_{22}H_{11}D_5F_2N_4O$ 396.1679, found = 396.1686.

Scheme 3. Synthesis of VU0481424 (**2**).[a]

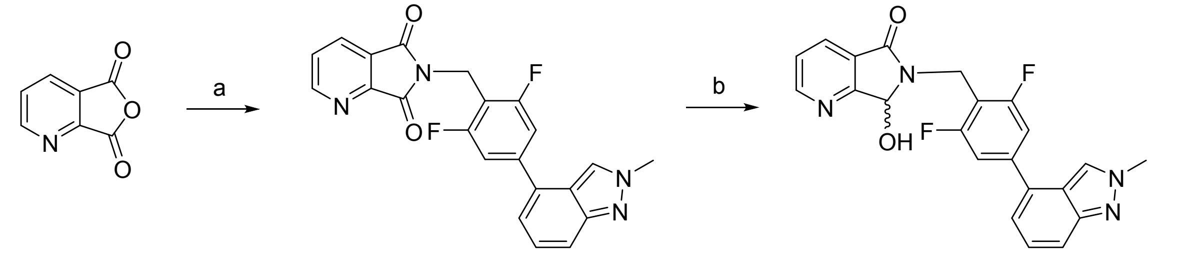

17 **18** **2,** VU0481424

[a] Reagents and conditions: (a) (2,6-difluoro-4-(2-mehtyl-2H-indazol-4-yl)phenyl)amine, Et_3N, DMF/MeCN, rt, 1 h, then HATU, 90%; (b) $NaBH_4$, DCM/MeOD, 24%

Step a. Synthesis of 6-(2,6-difluoro-4-(2-methyl-2*H*-indazol-4-yl)benzyl)-5*H*-pyrrolo[3,4-*b*]pyridine-5,7(6*H*)-dione (18)

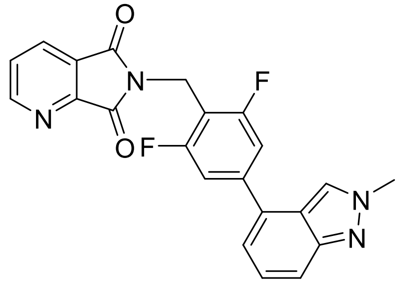

18

Quinolinic anhydride (300 mg, 2.01 mmol) and (2,6-difluoro-4-(2-methyl-2*H*-indazol-4-yl)phenyl)methanamine (550 mg, 2.01 mmol) were dissolved in MeCN (4 mL) and DMF (3 mL). The reaction mixture was then stirred at room temperature for 30 min. To this reaction mixture, Et_3N (1.12 mL, 8.05 mmol) was added and stirred overnight at room temperature. HATU (1530

mg, 4.02 mmol) was then added and stirred at room temperature for 1 h. Upon completion, the reaction mixture was diluted with EtOAc (50 mL), washed with H_2O (10 mL x 3), and dried over Na_2SO_4. The combined organics were concentrated under reduced pressure. The crude was then purified by flash column chromatography eluting 0-100% EtOAc in hexanes to give the title product (738.5 mg, 90%). LCMS ES-MS $[M+H]^+ = 405$.

Step b. Synthesis of 6-(2,6-difluoro-4-(2-methyl-2*H*-indazol-4-yl)benzyl)-7-hydroxy-6,7-dihydro-5*H*-pyrrolo[3,4-*b*]pyridin-5-one (**2,** VU0481424)

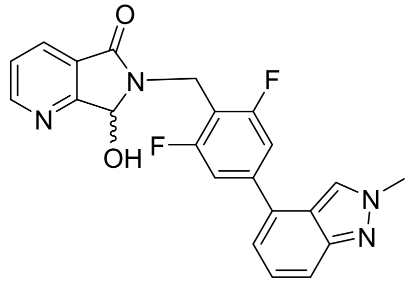

2 (VU0481424)

To a solution of 6-(2,6-difluoro-4-(2-methyl-2*H*-indazol-4-yl)benzyl)-5*H*-pyrrolo[3,4-*b*]pyridine-5,7(6*H*)-dione (700 mg, 1.73 mmol) in DCM (12 mL), $NaBH_4$ (328 mg, 8.66 mmol) was added. After 3 min at rt, MeOH (12 mL) was added dropwise. The reaction mixture was stirred at room temperature for 2 h. Upon completion, acetone (5 mL) was added and stirred for 10 min. The reaction mixture was diluted with H_2O (~20 mL) and extracted with CH_2Cl_2 (3x). The combined extracts were dried over Na_2SO_4, filtered, and concentrated to dryness. The crude was then purified by flash column chromatography eluting 0-20% MeOH in DCM to give the title product (172.3 mg, 24%). ^{1}H NMR (400 MHz, $CDCl_3$) δ 8.76 (dd, J = 5.1, 1.6 Hz, 1H), 8.18 (dd, J = 7.7, 1.5 Hz, 1H), 7.99 (s, 1H), 7.70 (d, J = 8.7 Hz, 1H), 7.51 (dd, J = 7.7, 5.0 Hz, 1H), 7.32 (dd, J = 8.7, 6.9 Hz, 1H), 7.23 – 7.16 (m, 2H), 7.09 (d, J = 6.9 Hz, 1H), 6.02 (s, 1H), 5.33 (d, J = 14.7 Hz, 1H), 4.77 (d, J = 14.6 Hz, 1H), 4.22 (s, 3H), OH is not shown; ^{13}C NMR (101 MHz, $CDCl_3$) δ 164.6, 164.0, 163.3 (d, J = 8.8 Hz), 160.8 (d, J = 9.2 Hz), 152.1, 149.5, 143.2 (t, J = 10.1 Hz), 133.0, 131.5 (t, J = 2.3 Hz), 126.3, 126.1, 125.0, 123.3, 121.3, 120.7, 117.9, 111.6 – 110.7 (m, 3C), 80.1, 40.6, 30.5 (t, J = 3.7 Hz); HRMS (ESI/Q-TOF) m/z: $[M + H]^+$ calcd for $C_{22}H_{16}F_2N_4O_2$, 407.1314; found = 407.1317. 6-(2,6-difluoro-4-(2-methyl-2*H*-indazol-4-yl)benzyl)-5-hydroxy-5,6-dihydro-7*H*-pyrrolo[3,4-*b*]pyridin-7-one was also isolated as a byproduct (51.5 mg). LCMS ES-MS $[M+H]^+ = 407$.

Scheme 4. Synthesis of VU6031240 (**4**) and VU6045587 (**6**).[a]

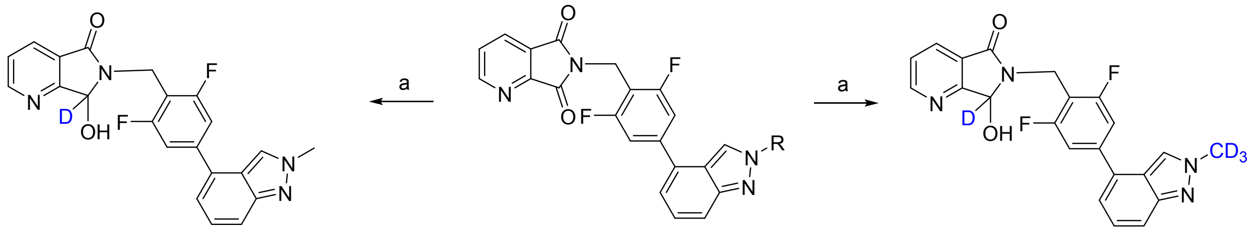

4

18, R = CH_3
19, R = CD_3

6

[a] Reagents and conditions: (a) $NaBD_4$, DCM/MeOD, 30-35%.

Synthesis of 6-(2,6-difluoro-4-(2-methyl-2*H*-indazol-4-yl)benzyl)-7-hydroxy-6,7-dihydro-5*H*-pyrrolo[3,4-*b*]pyridin-5-one-7-*d* (**4**, VU6031240)

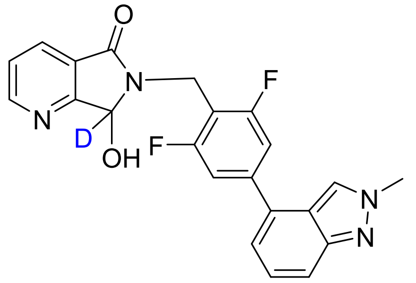

4 (VU6031240)

To a solution of 6-(2,6-difluoro-4-(2-methyl-2*H*-indazol-4-yl)benzyl)-5*H*-pyrrolo[3,4-*b*]pyridine-5,7(6*H*)-dione (450 mg, 1.11 mmol, 1.0 eq.) in DCM (7.5 mL), $NaBD_4$ (126 mg, 3.33 mmol, 3.0 eq.) was added and stirred at room temperature for 3 min. To this reaction mixture, CH_3OD (0.75 mL) was added dropwise. The reaction mixture was then stirred at room temperature for 30 min. Upon completion, the reaction mixture was quenched with acetone (2.5 mL) and stirred for 10 min. The reaction mixture was then diluted with H_2O (20 mL) and extracted with CH_2Cl_2 (3x). The combined extracts were dried over Na_2SO_4, filtered, and concentrated to dryness. The crude was then purified by flash column chromatography eluting 0-20% MeOH in DCM to give the title product (138 mg, 30%); ^1H NMR (400 MHz, $CDCl_3$) δ 8.77 (dd, J = 5.0, 1.6 Hz, 1H), 8.17 (dd, J = 7.7, 1.5 Hz, 1H), 8.00 (s, 1H), 7.71 (dt, J = 8.7, 0.9 Hz, 1H), 7.50 (dd, J = 7.7, 5.1 Hz, 1H), 7.33 (dd, J = 8.7, 6.9 Hz, 1H), 7.25 – 7.18 (m, 2H), 7.11 (dd, J = 6.9, 0.8 Hz, 1H), 6.46 (s, 1H), 5.33 (d,

J = 14.5 Hz, 1H), 4.76 (d, J = 14.7 Hz, 1H), 4.23 (s, 3H); ^{13}C NMR (101 MHz, $CDCl_3$) δ 164.61, 163.83, 163.34 (d, J = 8.9 Hz), 160.85 (d, J = 9.1 Hz), 152.18, 149.51, 143.27 (t, J = 10.0 Hz), 132.93, 131.49 (t, J = 2.5 Hz), 126.33, 126.15, 125.06, 123.33, 121.34, 120.73, 117.96, 111.55 – 110.77 (m, 3C), 77.36 (bs, CDOH), 40.67, 30.54 (t, J = 3.6 Hz); HRMS (ESI/Q-TOF) m/z: $[M + H]^+$ calcd for $C_{22}H_{15}DF_2N_4O_2$, 408.1377, found = 408.1377.

Synthesis of 6-(2,6-difluoro-4-(2-(methyl-*d*$_3$)-2*H*-indazol-4-yl)benzyl)-5*H*-pyrrolo[3,4-*b*]pyridine-5,7(6*H*)-dione (19)

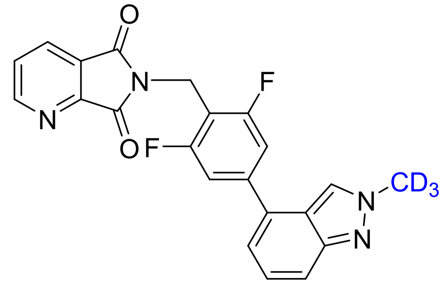

19

Quinolinic anhydride (582.2 mg, 3.91 mmol, 1.0 eq.) and (2,6-difluoro-4-(2-(methyl-*d*$_3$)-2*H*-indazol-4-yl)phenyl)methanamine (1080 mg, 3.91 mmol, 1.0 eq.) were dissolved in MeCN (15 mL). After 30 min at rt, Et_3N (1.10 mL, 7.82 mmol, 2.0 eq.) was added. The resulting mixture was allowed to stir at room temperature overnight. HATU (2972 mg, 7.82 mmol, 2.0 eq.) was then added and stirred at room temperature for 1 h. Upon completion, the reaction mixture was diluted with EtOAc (50 mL), washed with H_2O (3x), dried over Na_2SO_4, filtered and concentrated under reduced pressure. The crude material was purified by flash column chromatography eluting 0-100% EtOAc in hexanes to give the title product (595 mg, 37% yield). HRMS (ESI/Q-TOF) m/z: $[M + H]^+$ calcd for $C_{22}H_{11}D_3F_2N_4O_2$, 408.1346, found = 408.1347.

Synthesis of 6-(2,6-difluoro-4-(2-(methyl-*d*$_3$)-2*H*-indazol-4-yl)benzyl)-7-hydroxy-6,7-dihydro-5*H*-pyrrolo[3,4-*b*]pyridin-5-one-7-*d* (6, VU6045587)

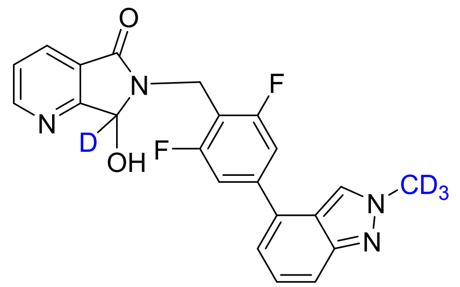

6 (VU6045587)

To a solution of 6-(2,6-difluoro-4-(2-(methyl-d_3)-2*H*-indazol-4-yl)benzyl)-5*H*-pyrrolo[3,4-*b*]pyridine-5,7(6*H*)-dione (595 mg, 1.46 mmol, 1.0 eq.) in DCM (13.3 mL), $NaBD_4$ (83 mg, 2.19 mmol, 1.5 eq.) was added. After 3 min at rt, CH_3OD (0.75 mL) was added dropwise. The reaction mixture was allowed to stir at room temperature for 30 min. Upon completion, acetone (2.5 mL) was added and stirred for 10 min. The reaction mixture was diluted with H_2O (~20 mL) and extracted with CH_2Cl_2 (3x). The combined extracts were dried over Na_2SO_4, filtered, and concentrated to dryness. The crude was then purified by flash column chromatography eluting 0-20% MeOH in DCM to give the title product (209 mg, 35%). ^{1}H NMR (400 MHz, DMSO-d_6) δ 8.78 (dd, *J* = 5.0, 1.6 Hz, 1H), 8.61 (d, *J* = 1.0 Hz, 1H), 8.10 (dd, *J* = 7.7, 1.6 Hz, 1H), 7.65 (dt, *J* = 8.6, 0.9 Hz, 1H), 7.57 (dd, *J* = 7.7, 5.0 Hz, 1H), 7.50 – 7.40 (m, 2H), 7.33 (dd, *J* = 8.5, 7.0 Hz, 1H), 7.26 (dd, *J* = 7.0, 0.9 Hz, 1H), 6.80 (s, 1H), 5.03 (d, *J* = 14.8 Hz, 1H), 4.61 (d, *J* = 14.9 Hz, 1H); ^{13}C NMR (101 MHz, $CDCl_3$) δ 164.52, 163.69, 163.34 (d, *J* = 8.9 Hz), 160.86 (d, *J* = 9.1 Hz), 151.97, 149.35, 143.23 (t, *J* = 10.1 Hz), 133.10, 131.52 (t, *J* = 2.4 Hz), 126.43, 126.28, 125.13, 123.41, 121.40, 117.89, 111.63 – 110.71 (m, 3C), 80.52 – 79.15 (m, CD_2), 40.84 – 39.17 (m, CD_3), 30.58 (t, *J* = 3.5 Hz); HRMS (ESI/Q-TOF) m/z: $[M + H]^+$ calcd for $C_{22}H_{12}D_4F_2N_4O_2$, 411.1565, found = 411.1567.

NMR Spectra

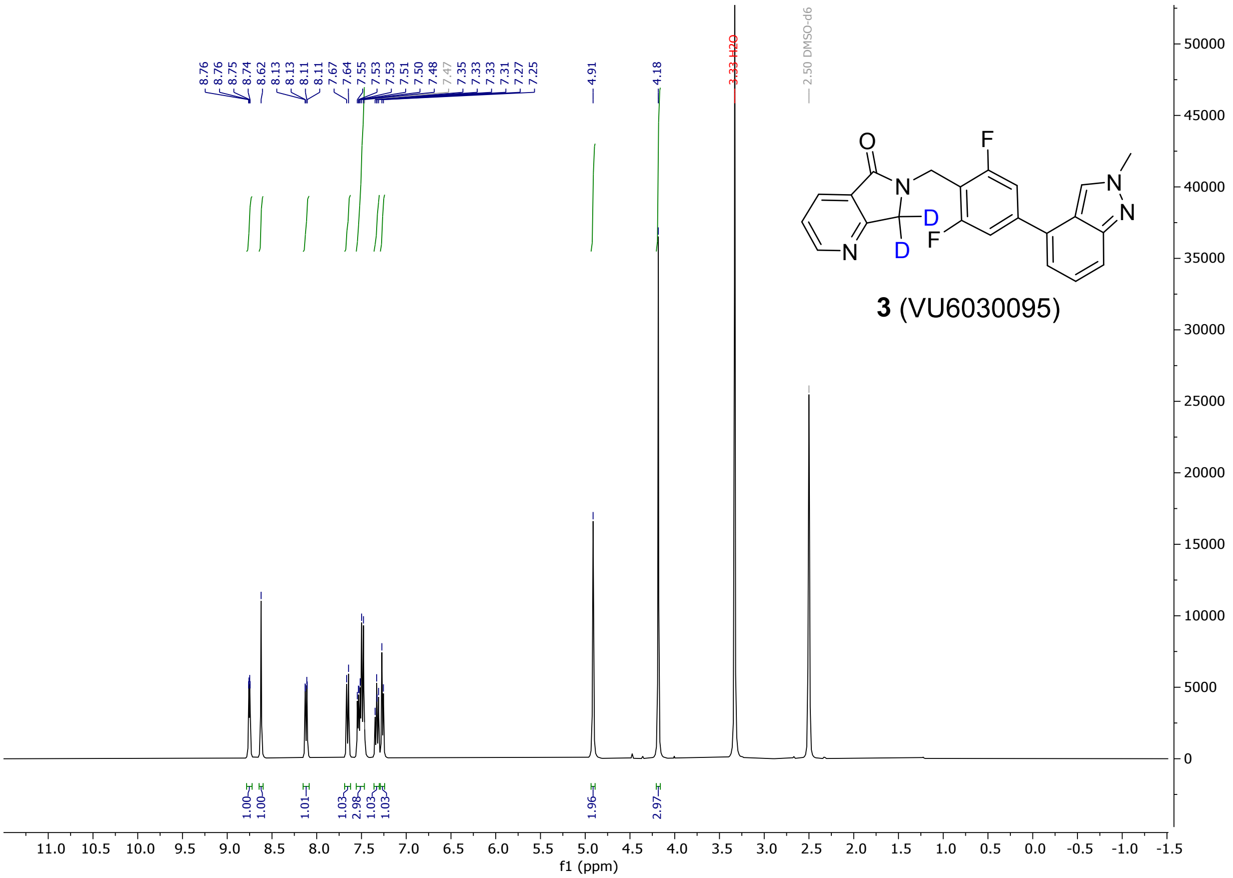

^{1}H NMR (400 MHz, DMSO-d_6) δ 8.75 (dd, J = 5.0, 1.6 Hz, 1H), 8.62 (s, 1H), 8.12 (dd, J = 7.7, 1.6 Hz, 1H), 7.66 (d, J = 8.5 Hz, 1H), 7.57 – 7.44 (m, 3H), 7.33 (dd, J = 8.5, 7.0 Hz, 1H), 7.26 (d, J = 6.9 Hz, 1H), 4.91 (s, 2H), 4.18 (s, 3H).

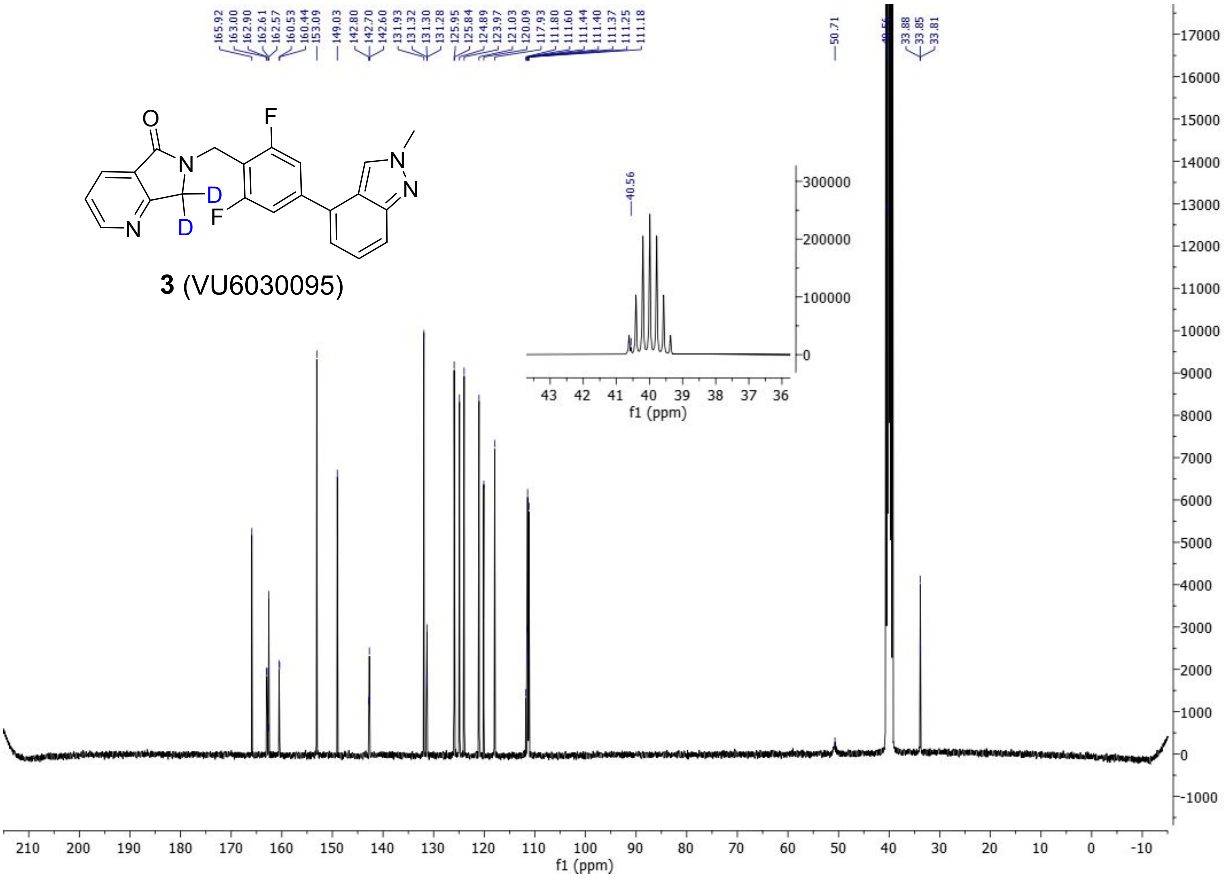

^{13}C NMR (101 MHz, DMSO-d_6) δ 165.92, 162.95 (d, J = 9.3 Hz), 162.57, 160.49 (d, J = 9.2 Hz), 153.09, 149.03, 142.70 (t, J = 10.1 Hz), 131.93, 131.30 (t, J = 2.4 Hz), 125.95, 125.84, 124.89, 123.97, 121.03, 120.09, 117.93, 111.92 – 111.00 (m, 3C), 51.41 – 50.05 (m, CD_2), 40.56, 33.85 (t, J = 3.4 Hz).

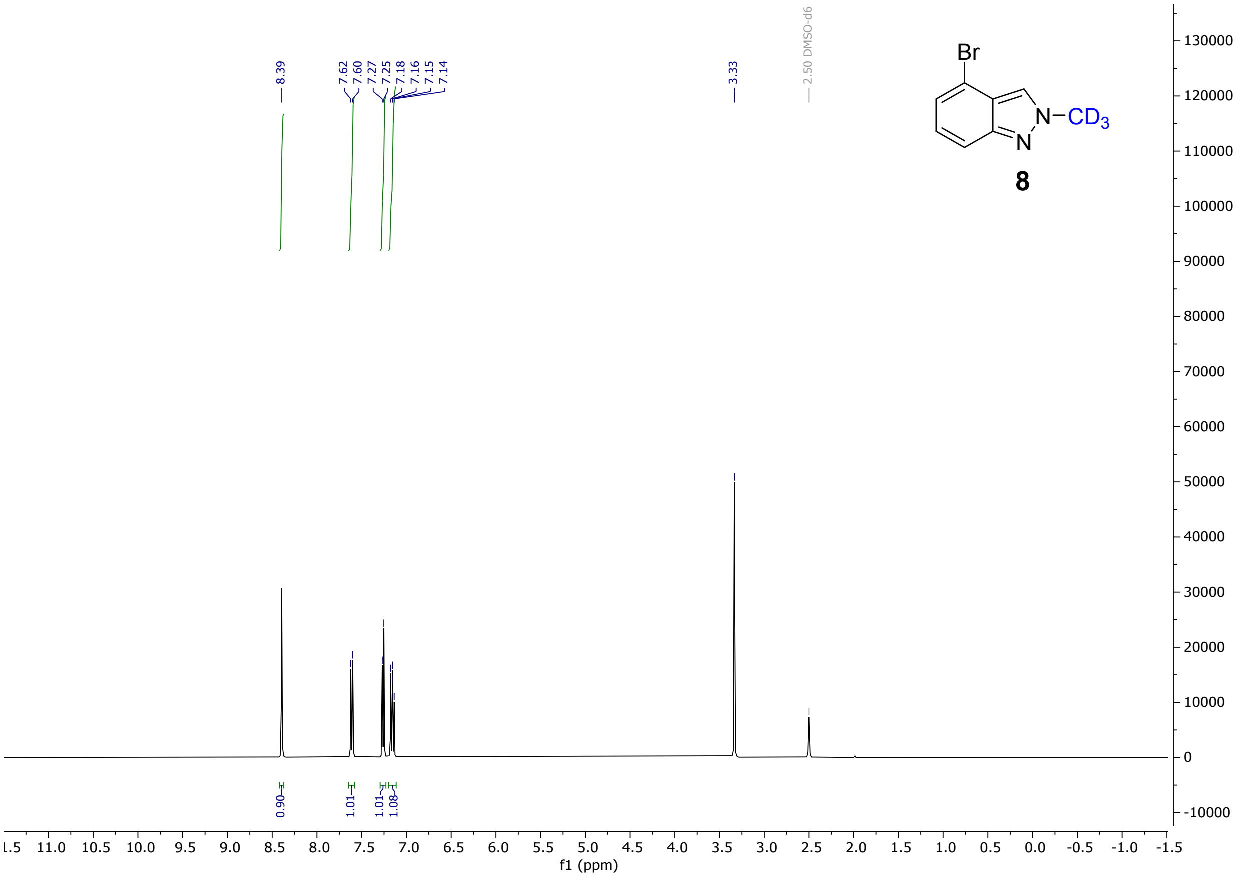

^{1}H NMR (400 MHz, DMSO-d_6) δ 8.39 (d, J = 0.9 Hz, 1H), 7.62 (dd, J = 8.5, 1.1 Hz, 1H), 7.26 (d, J = 7.1 Hz, 1H), 7.16 (dd, J = 8.6, 7.2 Hz, 1H).

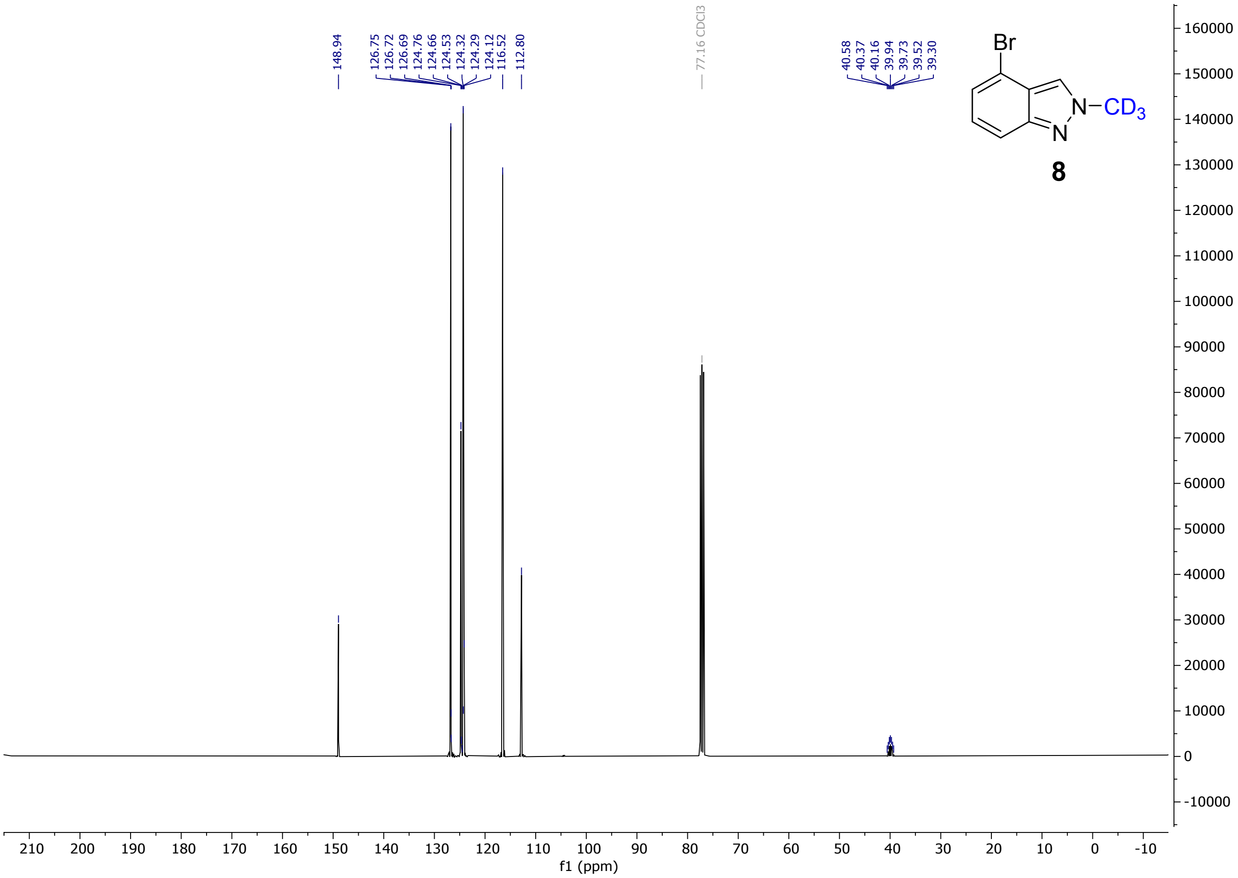

^{13}C NMR (101 MHz, $CDCl_3$) δ 148.94, 126.75, 124.76, 124.32, 124.12, 116.52, 112.80, 39.88 (dt, J = 42.9, 21.5 Hz, CD_3).

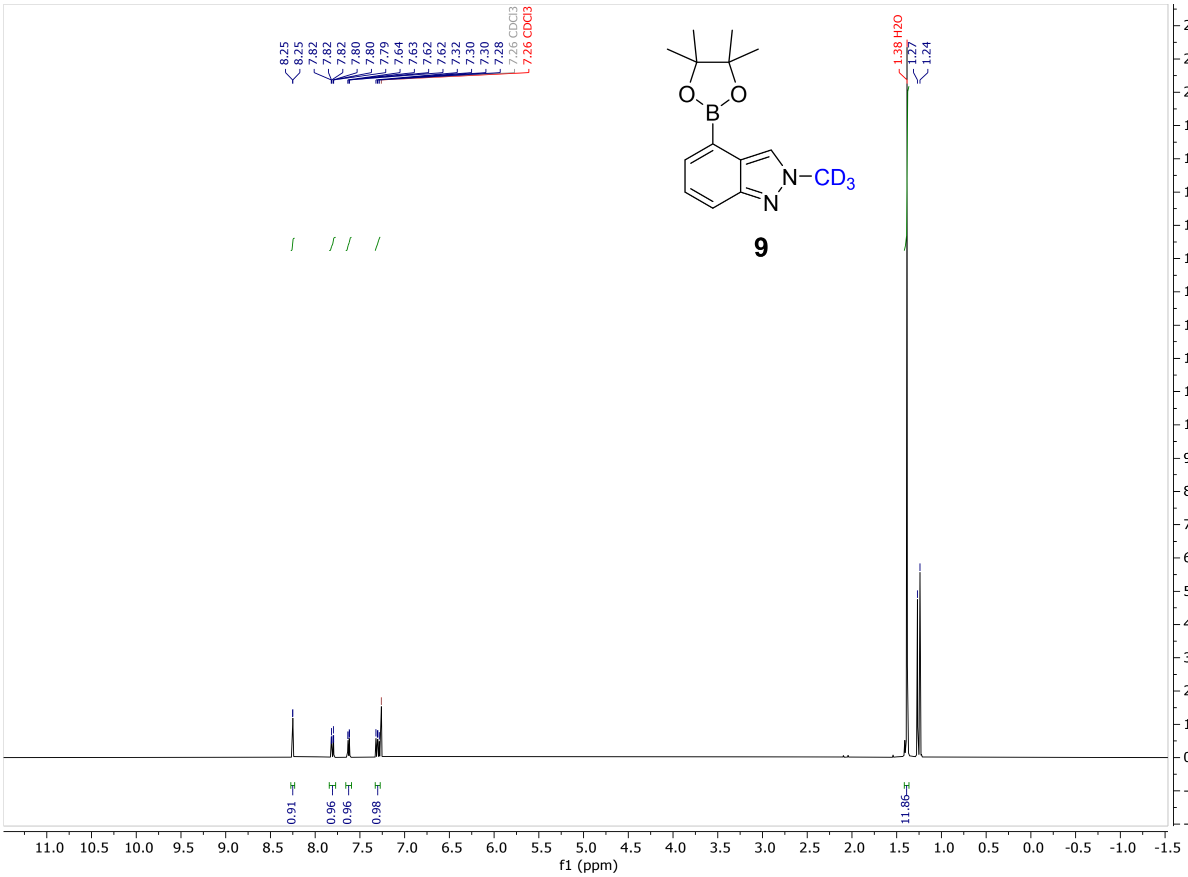

^{1}H NMR (400 MHz, $CDCl_3$) δ 8.25 (d, J = 1.0 Hz, 1H s, 1H), 7.81 (dt, J = 8.7, 1.0 Hz, 1H), 7.63 (dd, J = 6.6, 0.9 Hz, 1H), 7.30 (dd, J = 8.7, 6.6 Hz, 1H), 1.38 (s, 12H). Note: peaks at δ 83.21 and 24.73 represent unreacted bis(pincolato)diboron.

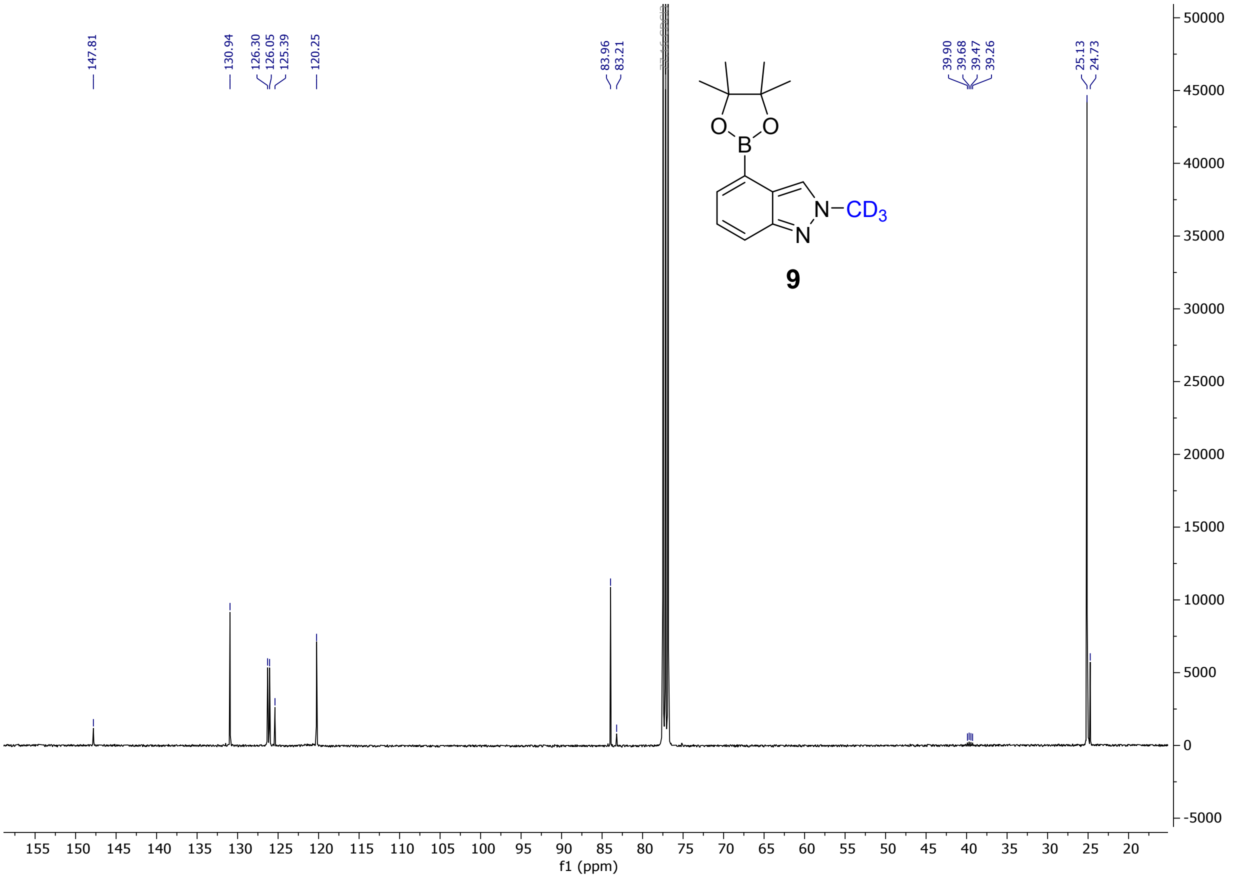

^{13}C NMR (101 MHz, $CDCl_3$) δ 147.81, 130.94, 126.30, 126.05, 125.39, 120.25, 83.96, 83.21 (2C), 39.58 (dt, J = 42.9, 21.3 Hz, CD_3), 25.13 (4C). Note: peaks at δ 83.21 and 24.73 represent unreacted bis(pincolato)diboron.

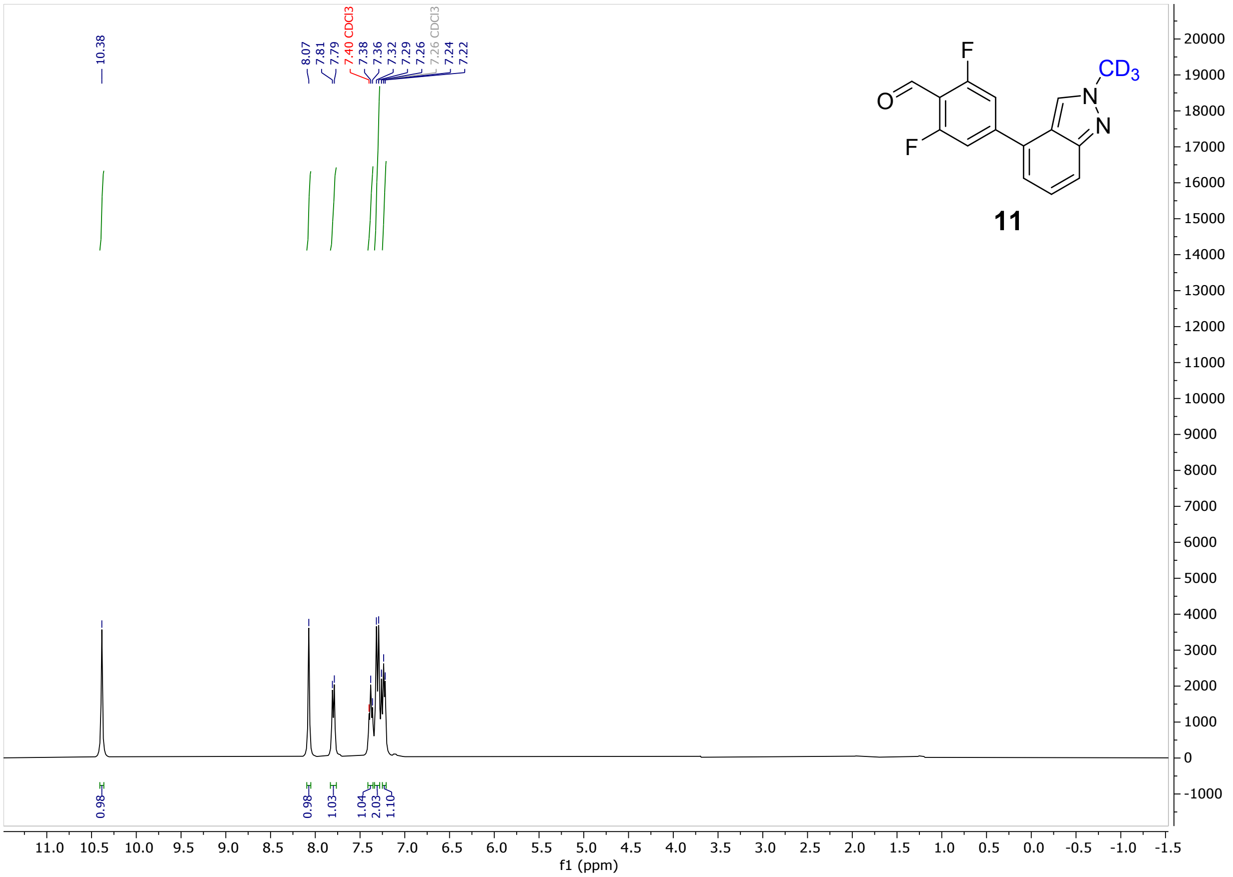

^{1}H NMR (400 MHz, $CDCl_3$) δ 10.38 (s, 1H), 8.07 (s, 1H), 7.80 (d, J = 8.7 Hz, 1H), 7.40 (dd, J = 8.7, 7.0 Hz, 1H), 7.38 – 7.26 (m, 2H), 7.23 (d, J = 7.0 Hz, 1H).

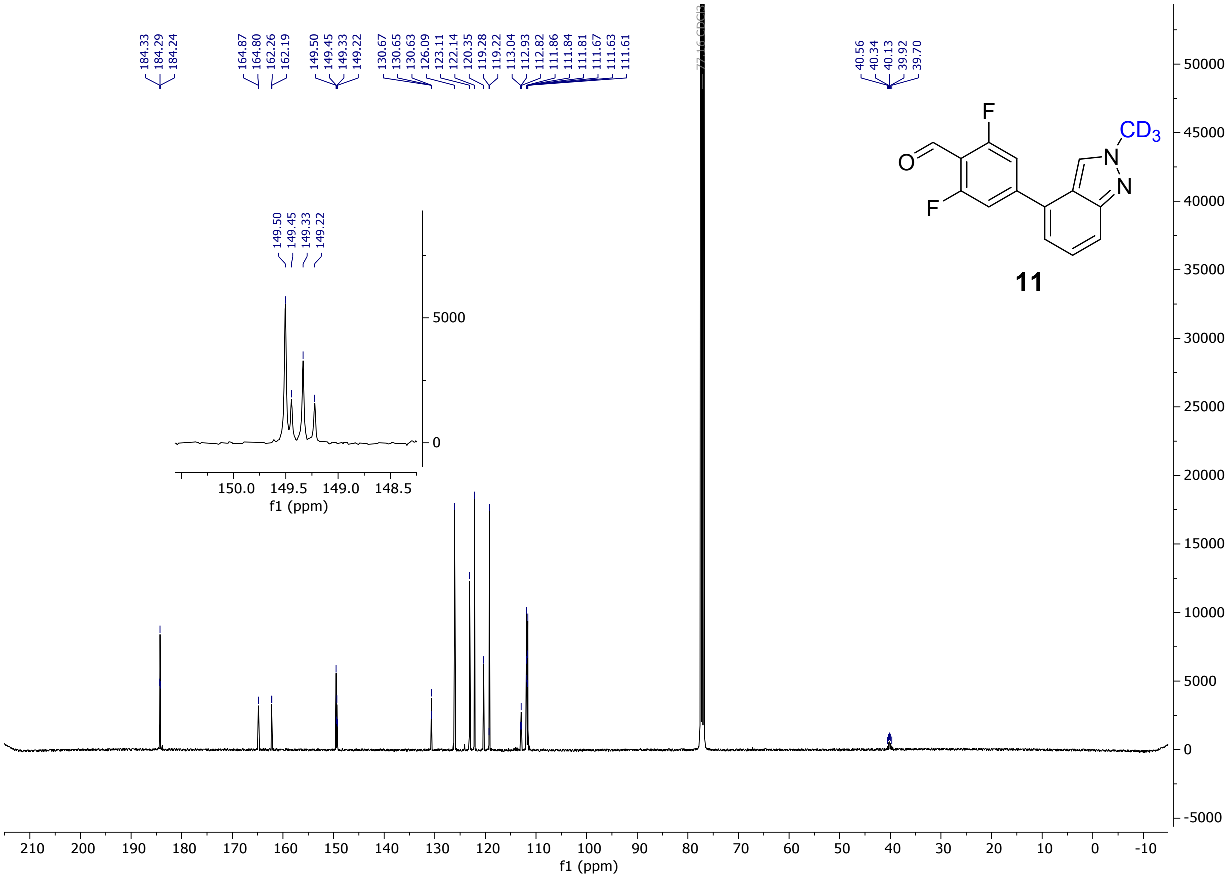

11

^{13}C NMR (101 MHz, $CDCl_3$) δ 184.29 (t, J = 4.3 Hz), 164.84 (d, J = 6.7 Hz), 162.22 (d, J = 6.8 Hz), 149.50, 149.34 (t, J = 11.2 Hz), 130.65 (t, J = 2.3 Hz), 126.09, 123.11, 122.14, 120.35, 119.22, 112.93 (t, J = 11.1 Hz), 112.15 – 111.45 (m, 2C), 40.13 (dt, J = 43.1, 21.5 Hz, CD_3).

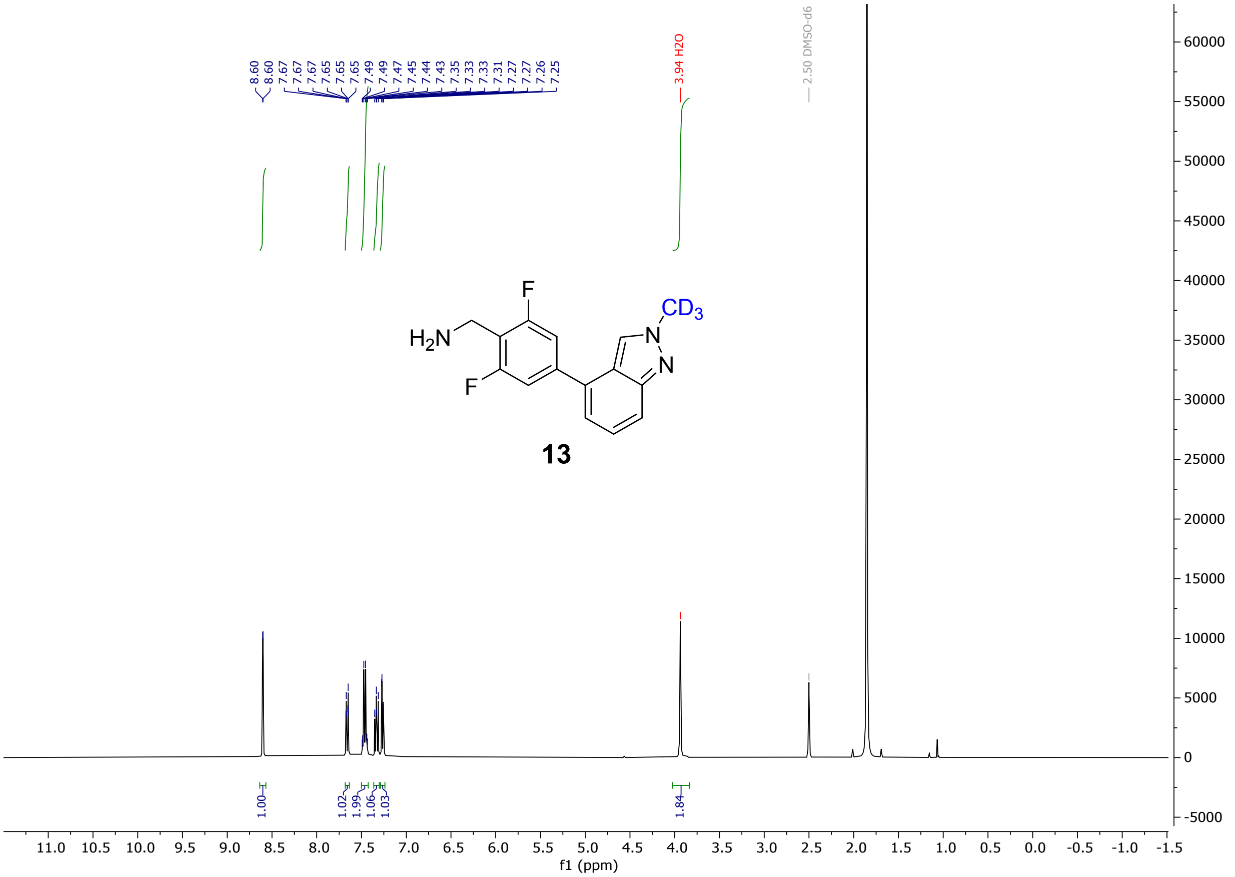

^{1}H NMR (400 MHz, DMSO) δ 8.60 (d, *J* = 1.0 Hz, 1H), 7.66 (dt, *J* = 8.6, 1.0 Hz, 1H), 7.52 – 7.41 (m, 2H), 7.33 (dd, *J* = 8.5, 7.0 Hz, 1H), 7.26 (dd, *J* = 6.9, 0.9 Hz, 1H), 3.94 (s, 2H). Note: peak at δ 1.85 represents excess acetic acid.

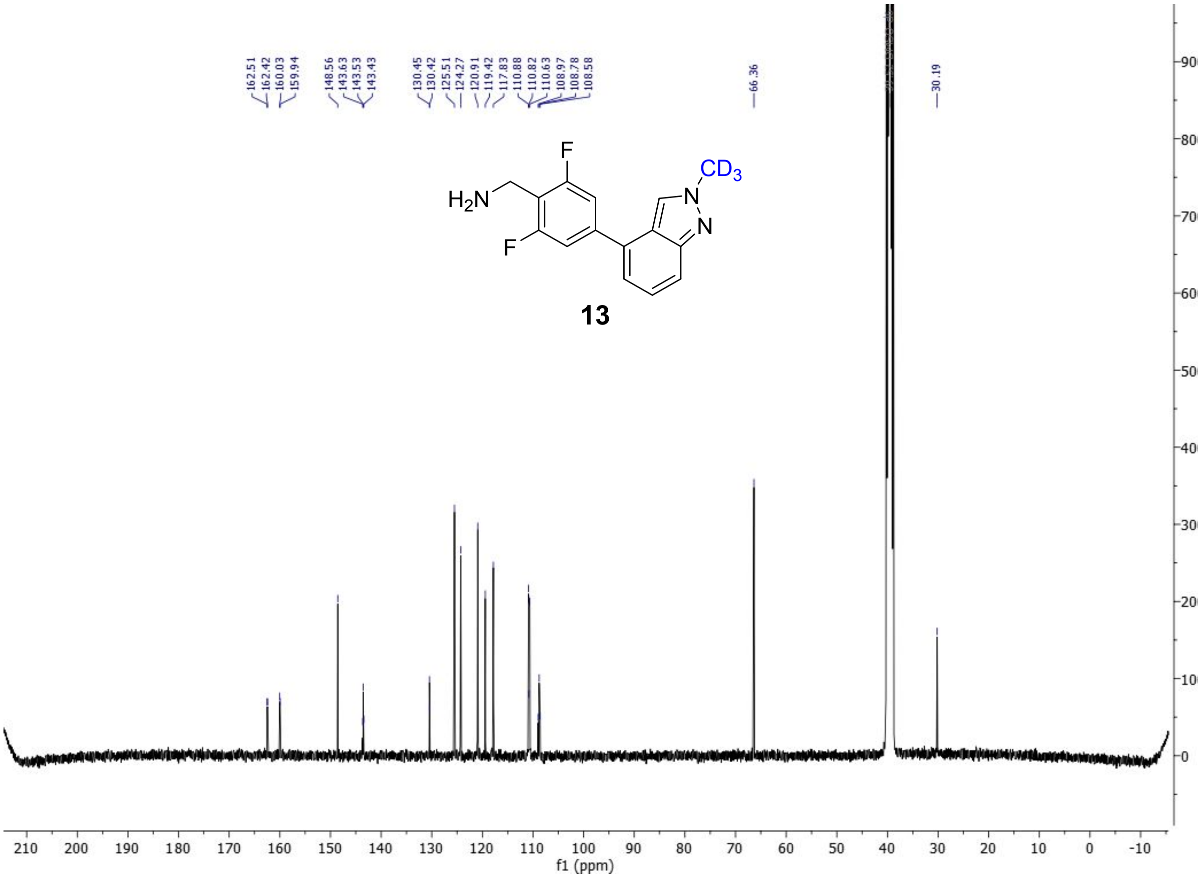

^{13}C NMR (101 MHz, DMSO-d_6δ 162.46 (d, J = 8.7 Hz), 159.99 (d, J = 8.6 Hz), 148.56, 143.53 (t, J = 10.3 Hz), 130.44 (d, J = 2.7 Hz), 125.51, 124.27, 120.91, 119.42, 117.83, 111.25 – 110.30 (m, 2C), 108.78 (t, J = 19.9 Hz), 66.36, 30.19, CD_3 is hidden under DMSO-d_6.

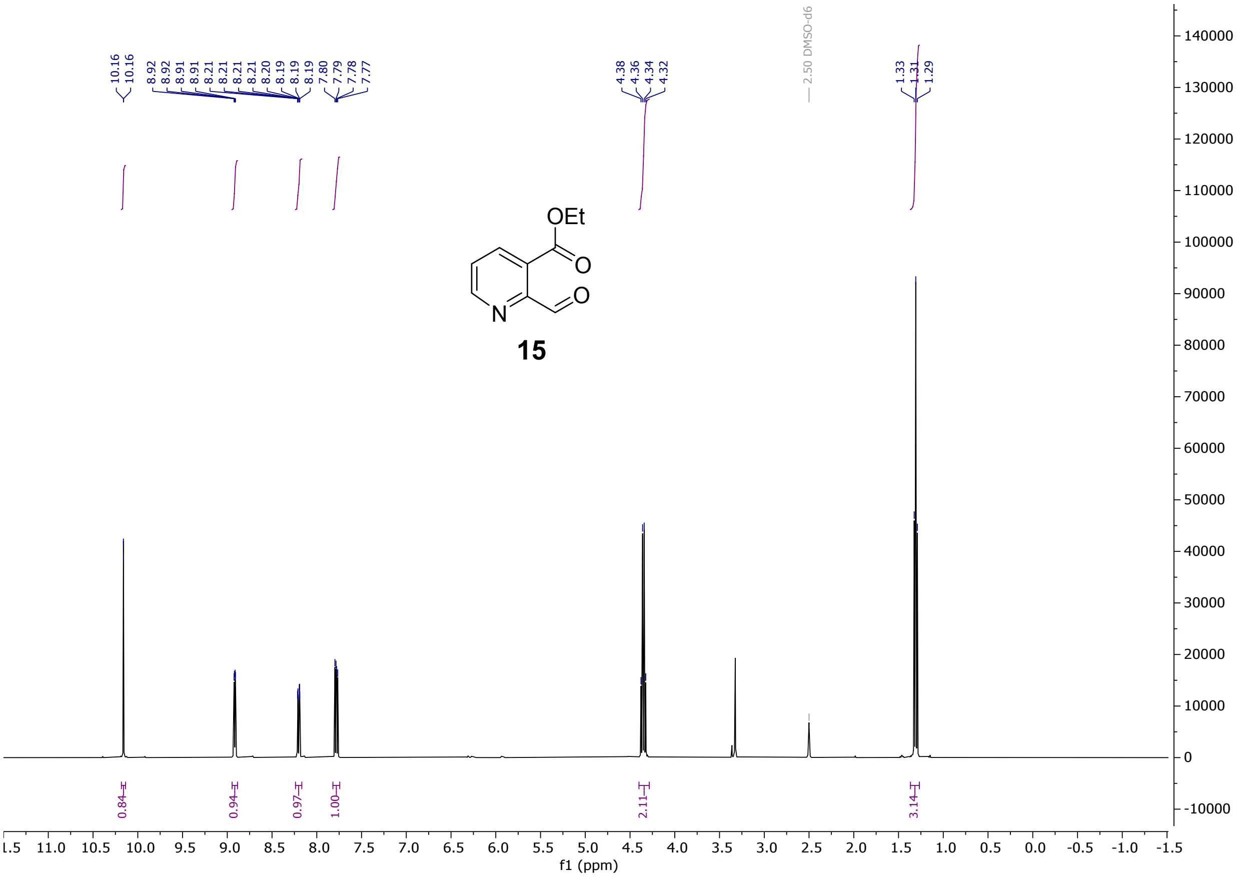

^{1}H NMR (400 MHz, DMSO-d_6) δ 10.16 (s, 1H), 8.92 (dd, J = 4.8, 1.0 Hz, 1H), 8.20 (ddd, J = 7.9, 1.6, 0.5 Hz, 1H), 7.78 (dd, J = 7.8, 4.7 Hz, 1H), 4.35 (q, J = 7.1 Hz, 2H), 1.31 (t, J = 7.1 Hz, 3H).

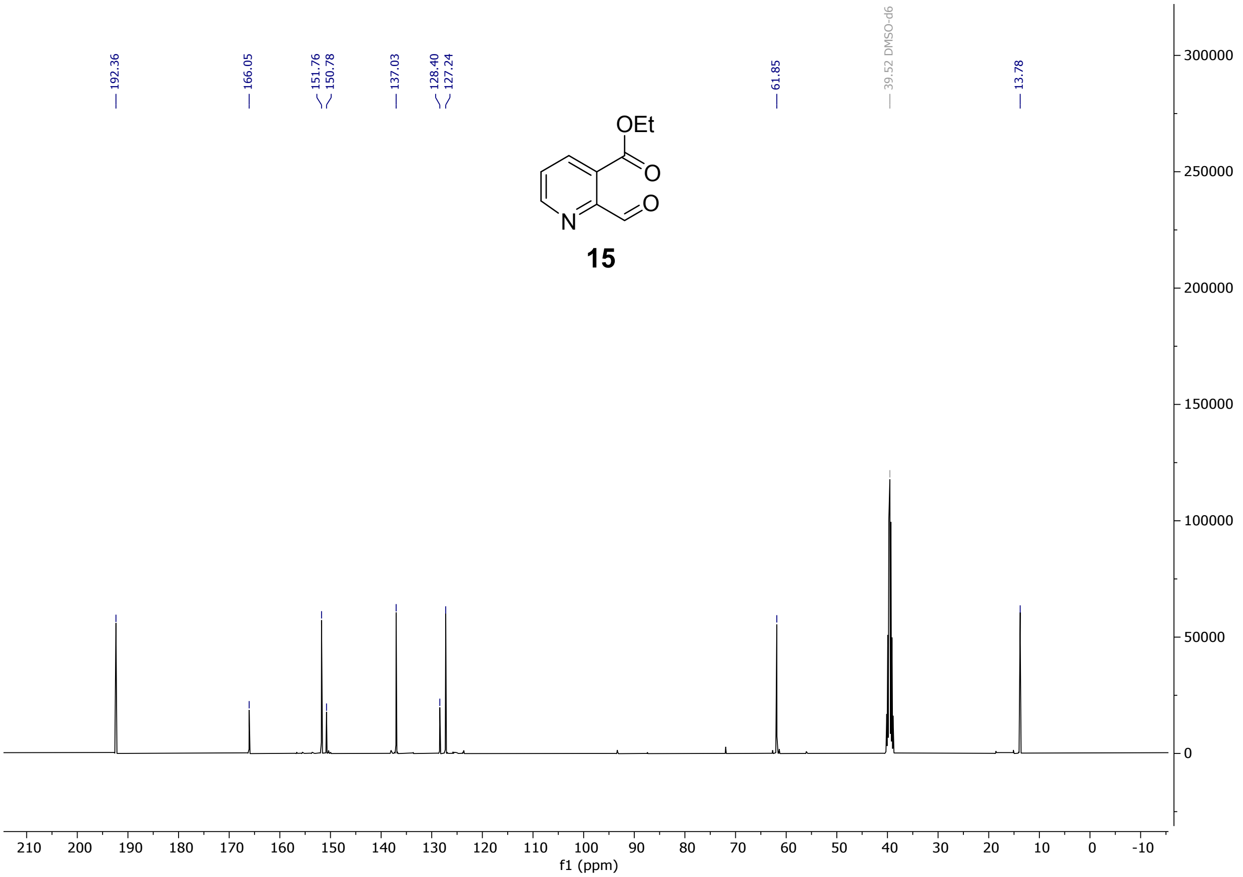

^{13}C NMR (101 MHz, DMSO-d_6) δ 192.36, 166.05, 151.76, 150.78, 137.03, 128.40, 127.24, 61.85, 13.78.

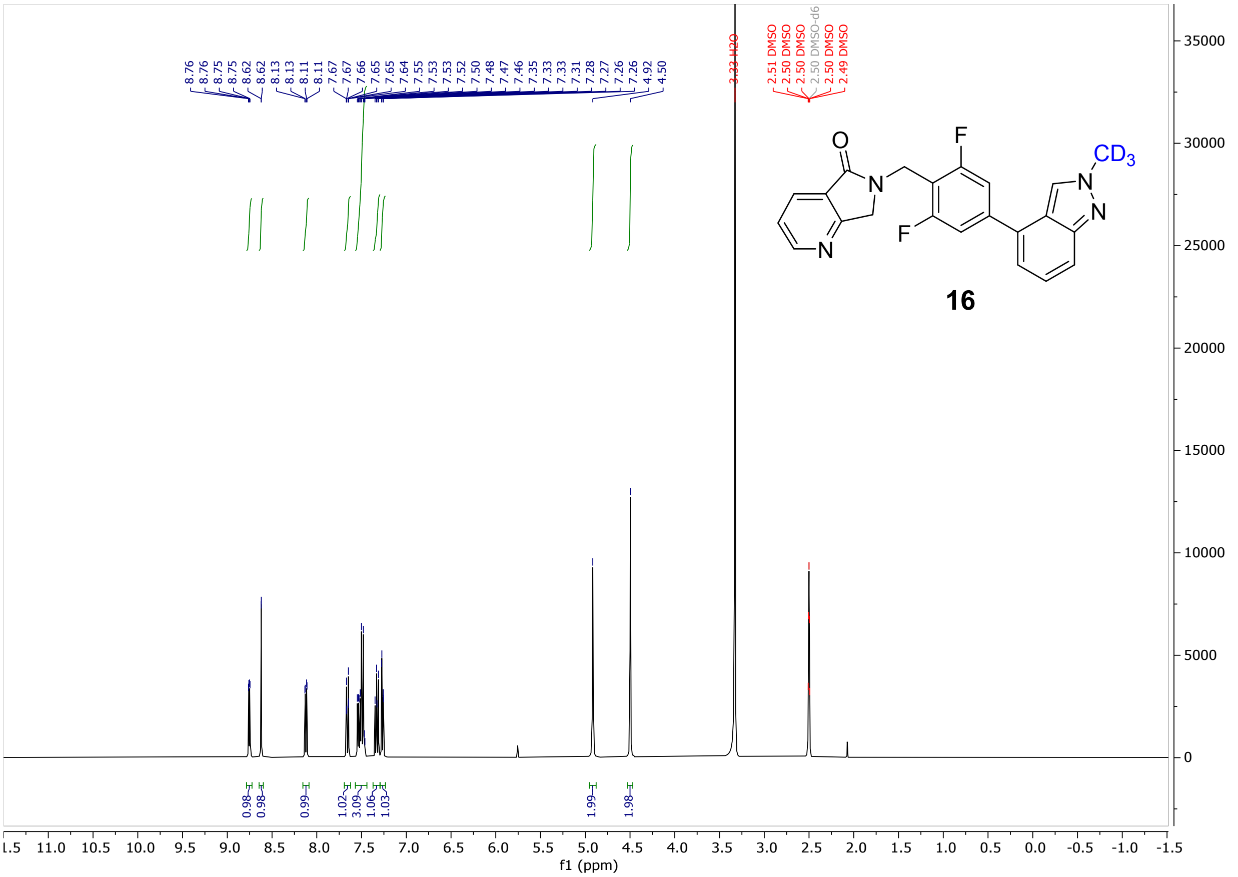

^{1}H NMR (400 MHz, DMSO-d_6) δ 8.75 (dd, J = 4.9, 1.6 Hz, 1H), 8.62 (d, J = 1.0 Hz, 1H), 8.12 (dd, J = 7.7, 1.6 Hz, 1H), 7.66 (dt, J = 8.5, 1.0 Hz, 1H), 7.57 – 7.43 (m, 3H), 7.33 (dd, J = 8.5, 7.0 Hz, 1H), 7.26 (dd, J = 7.0, 0.9 Hz, 1H), 4.92 (s, 2H), 4.50 (s, 2H).

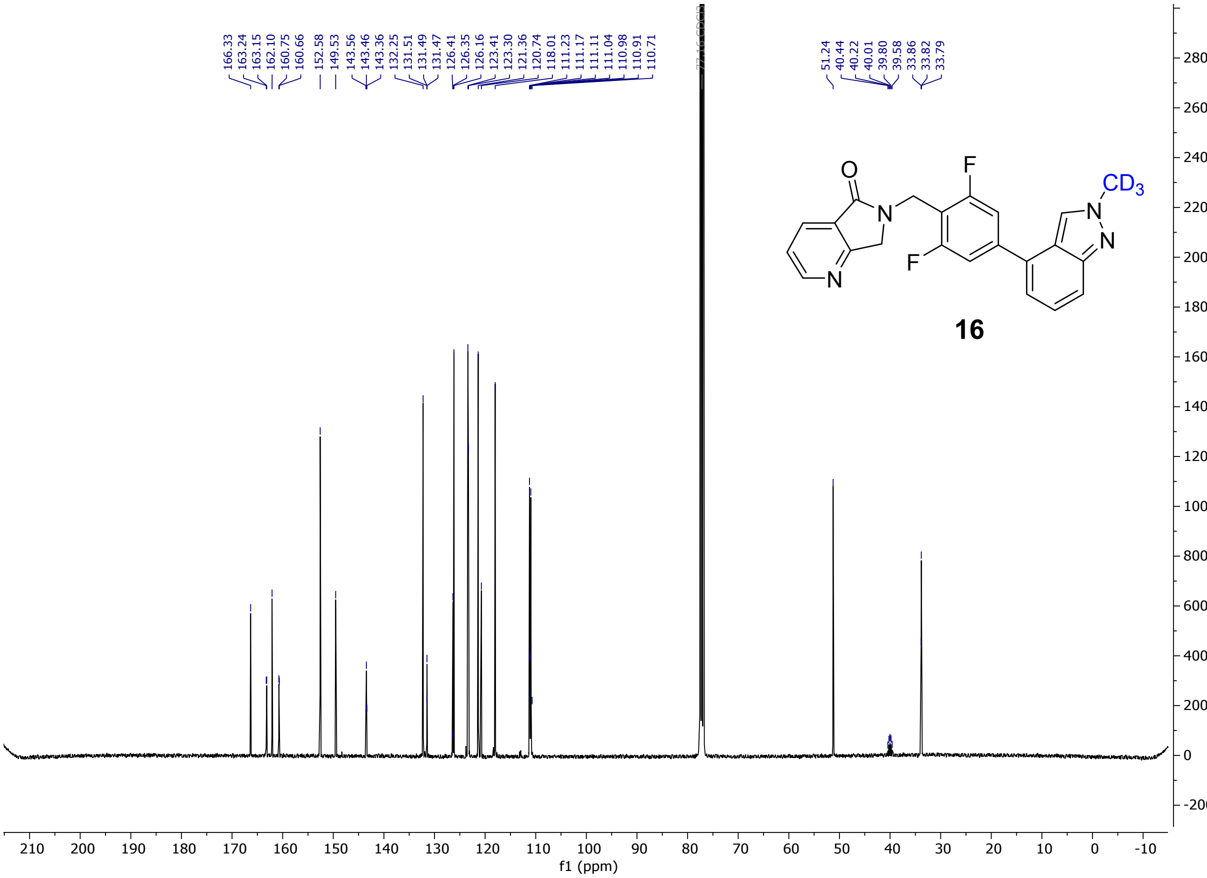

^{13}C NMR (101 MHz, $CDCl_3$) δ 166.33, 163.19 (d, J = 8.8 Hz), 162.10, 160.71 (d, J = 8.8 Hz), 152.58, 149.53, 143.46 (t, J = 10.0 Hz), 132.25, 131.49 (t, J = 2.4 Hz), 126.35, 126.16, 123.41, 123.30, 121.36, 120.74, 118.01, 111.47 – 110.46 (m, 3C), 51.24, 40.01 (dt, J = 42.1, 21.6 Hz, CD_3), 33.82 (t, J = 3.5 Hz).

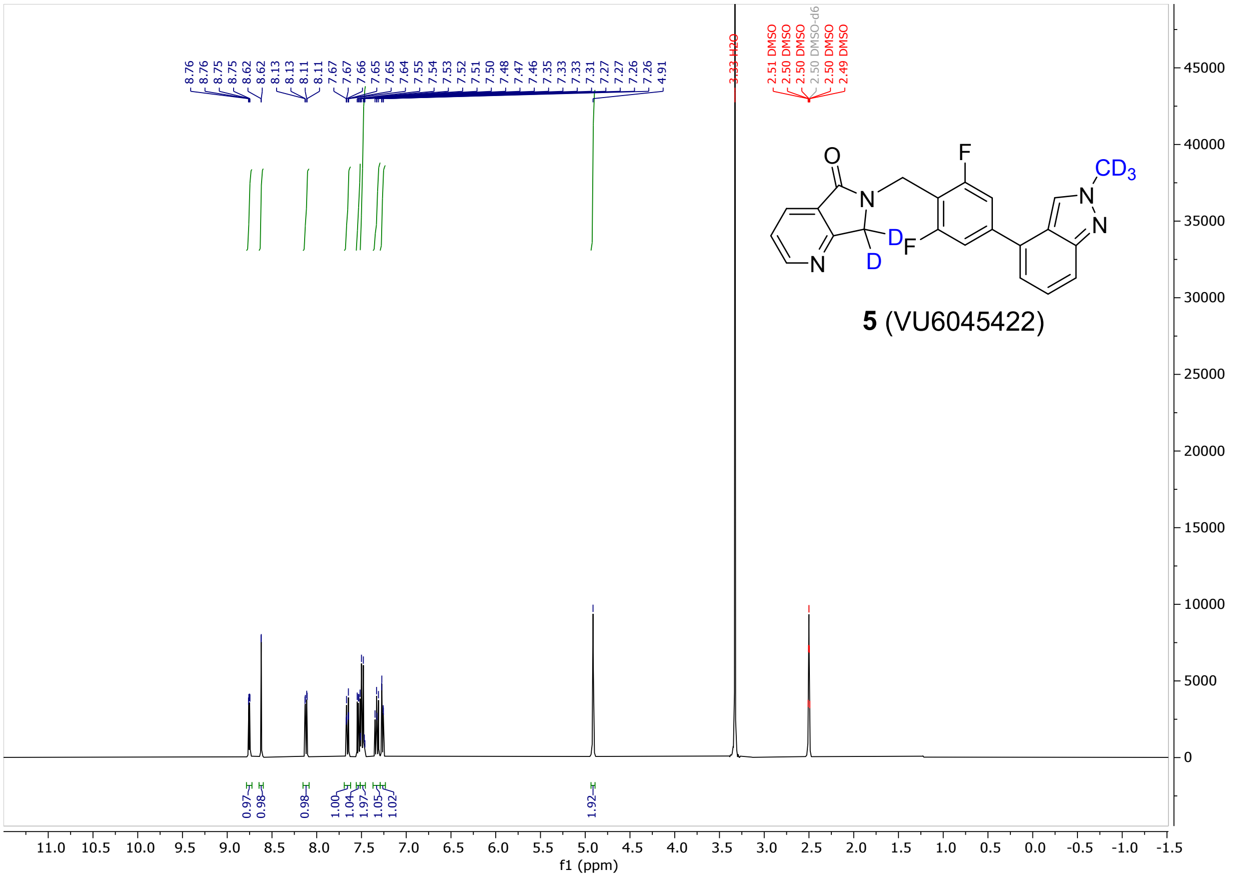

^{1}H NMR (400 MHz, DMSO-d_6) δ 8.75 (dd, J = 4.9, 1.6 Hz, 1H), 8.62 (d, J = 1.0 Hz, 1H), 8.12 (dd, J = 7.7, 1.6 Hz, 1H), 7.66 (d, J = 8.5 Hz, 1H), 7.57 – 7.44 (m, 3H), 7.33 (dd, J = 8.5, 7.0 Hz, 1H), 7.27 (dd, J = 7.0, 0.9 Hz, 1H), 4.91 (s, 2H).

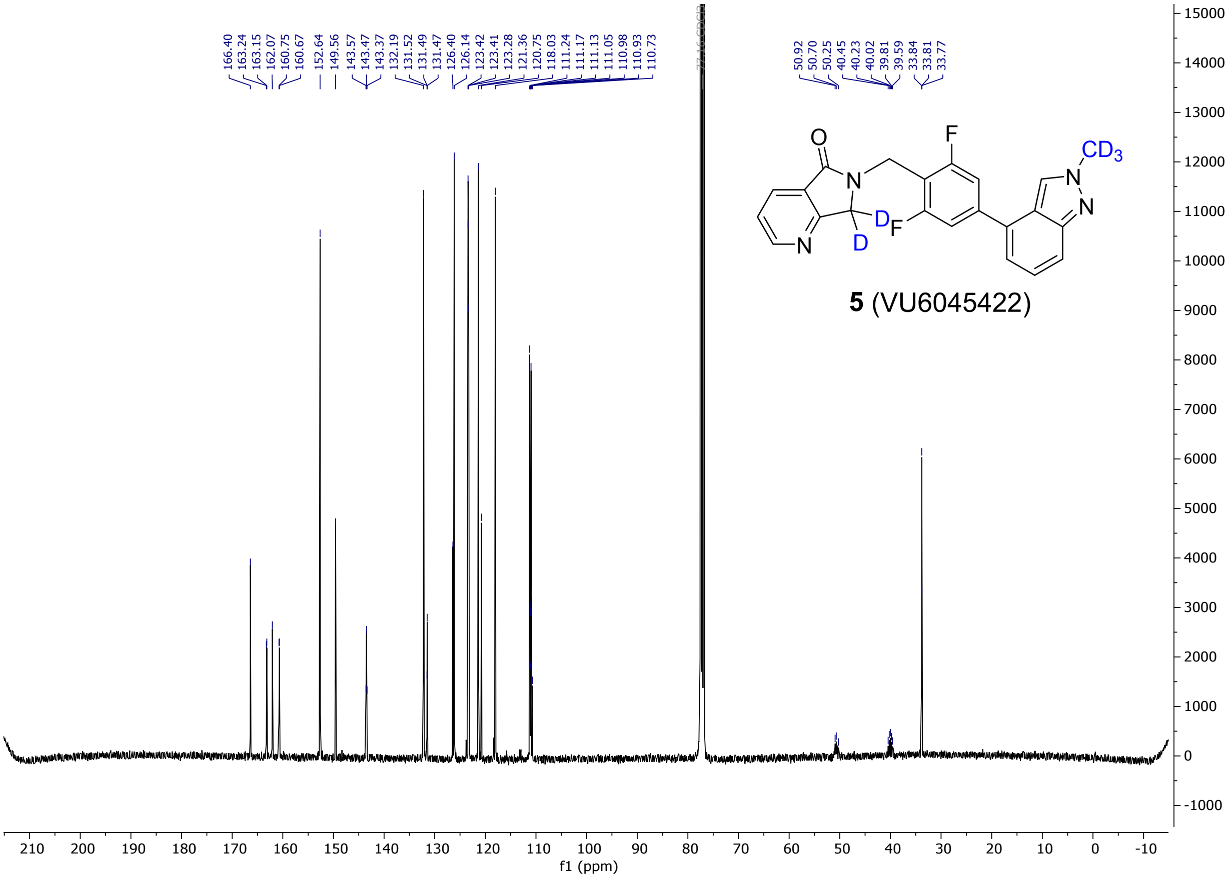

5 (VU6045422)

^{13}C NMR (101 MHz, $CDCl_3$) δ 166.40, 163.20 (d, J = 8.8 Hz), 162.07, 160.71 (d, J = 8.8 Hz), 152.64, 149.56, 143.47 (t, J = 10.1 Hz), 132.19, 131.49 (t, J = 2.4 Hz), 126.40, 126.14, 123.42, 123.28, 121.36, 120.75, 118.03, 111.49 – 110.57 (m, 3C), 51.67 – 49.52 (m, CD_2), 40.88 – 38.79 (m, CD_3), 33.81 (t, J = 3.5 Hz).

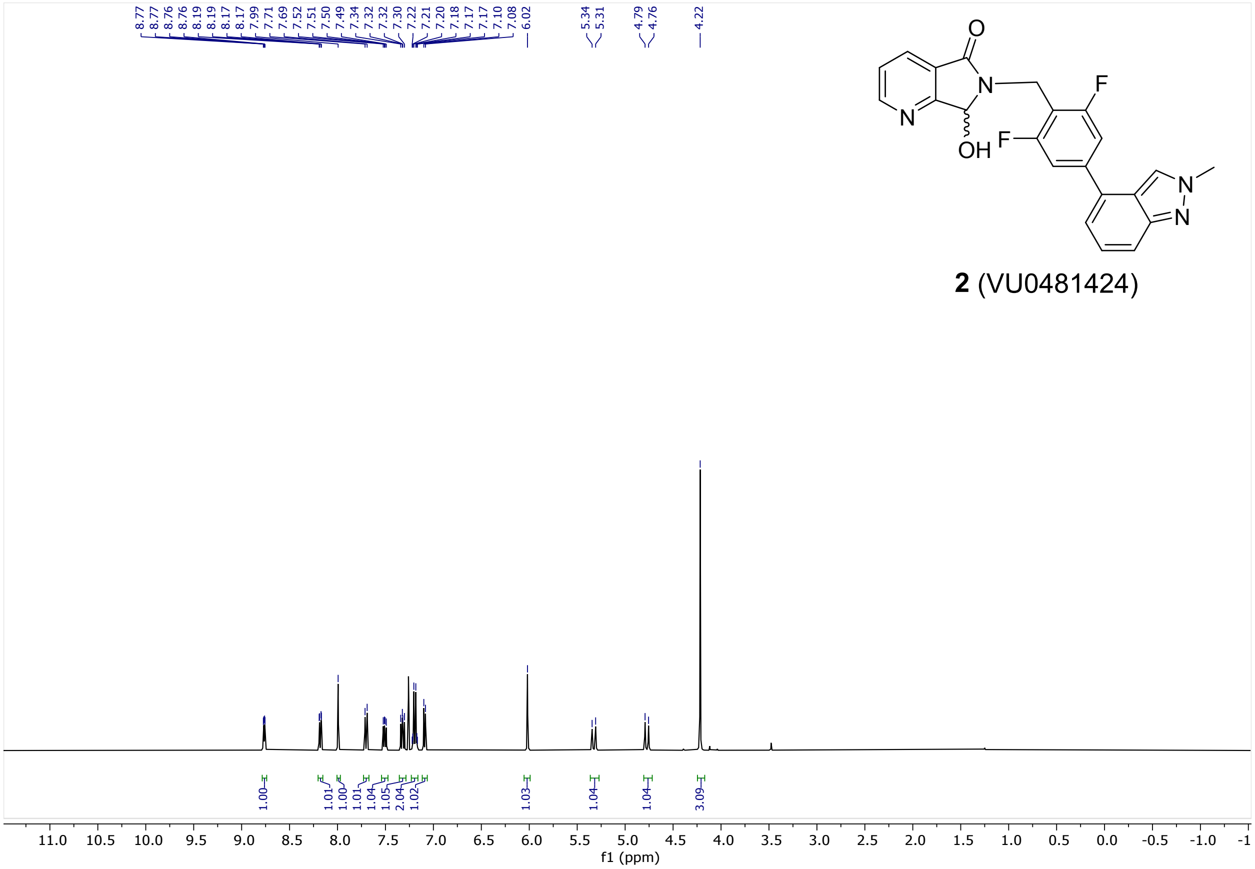

2 (VU0481424)

^{1}H NMR (400 MHz, $CDCl_3$) δ 8.76 (dd, *J* = 5.1, 1.6 Hz, 1H), 8.18 (dd, *J* = 7.7, 1.5 Hz, 1H), 7.99 (s, 1H), 7.70 (d, *J* = 8.7 Hz, 1H), 7.51 (dd, *J* = 7.7, 5.0 Hz, 1H), 7.32 (dd, *J* = 8.7, 6.9 Hz, 1H), 7.23 – 7.16 (m, 2H), 7.09 (d, *J* = 6.9 Hz, 1H), 6.02 (s, 1H), 5.33 (d, *J* = 14.7 Hz, 1H), 4.77 (d, *J* = 14.6 Hz, 1H), 4.22 (s, 3H), OH is not shown.

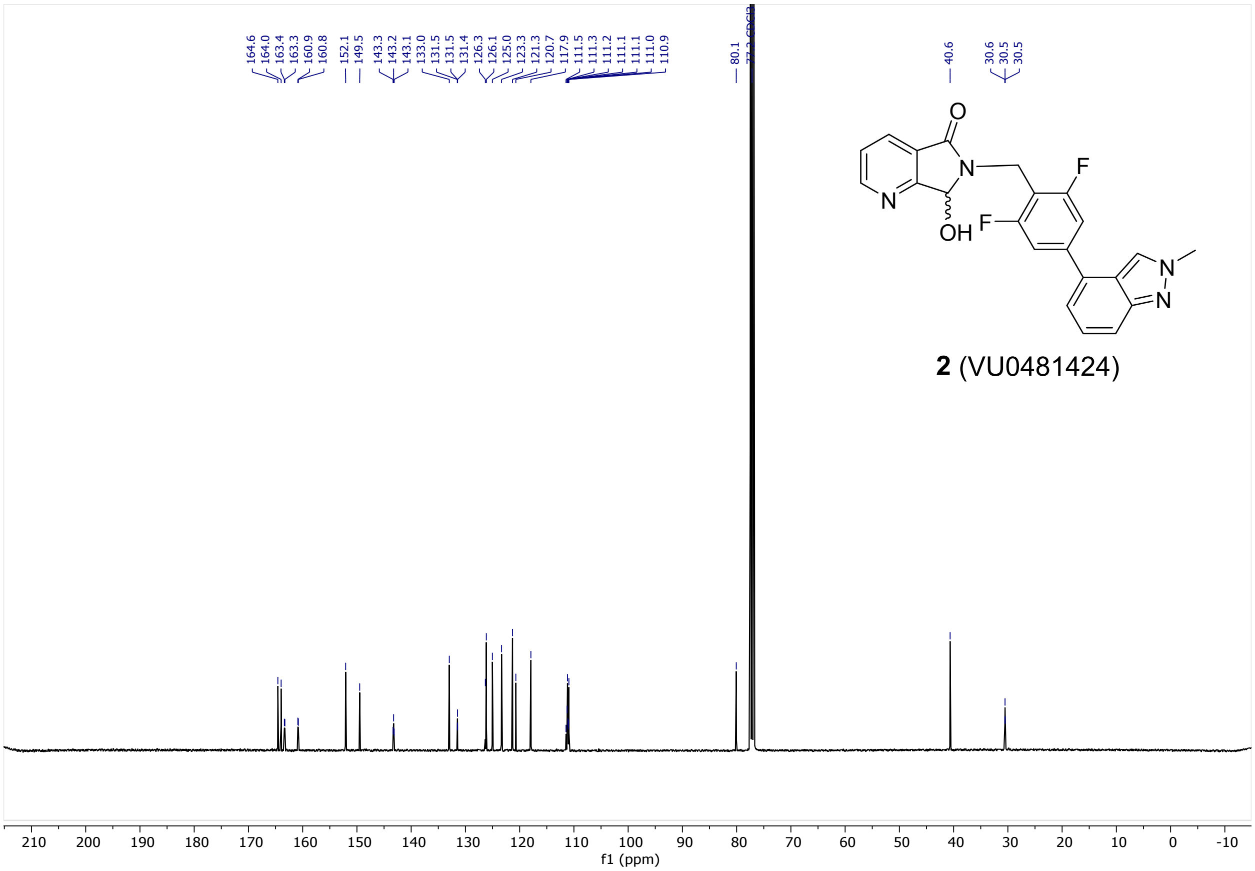

^{13}C NMR (101 MHz, $CDCl_3$) δ 164.6, 164.0, 163.3 (d, J = 8.8 Hz), 160.8 (d, J = 9.2 Hz), 152.1, 149.5, 143.2 (t, J = 10.1 Hz), 133.0, 131.5 (t, J = 2.3 Hz), 126.3, 126.1, 125.0, 123.3, 121.3, 120.7, 117.9, 111.6 – 110.7 (m, 3C), 80.1, 40.6, 30.5 (t, J = 3.7 Hz).

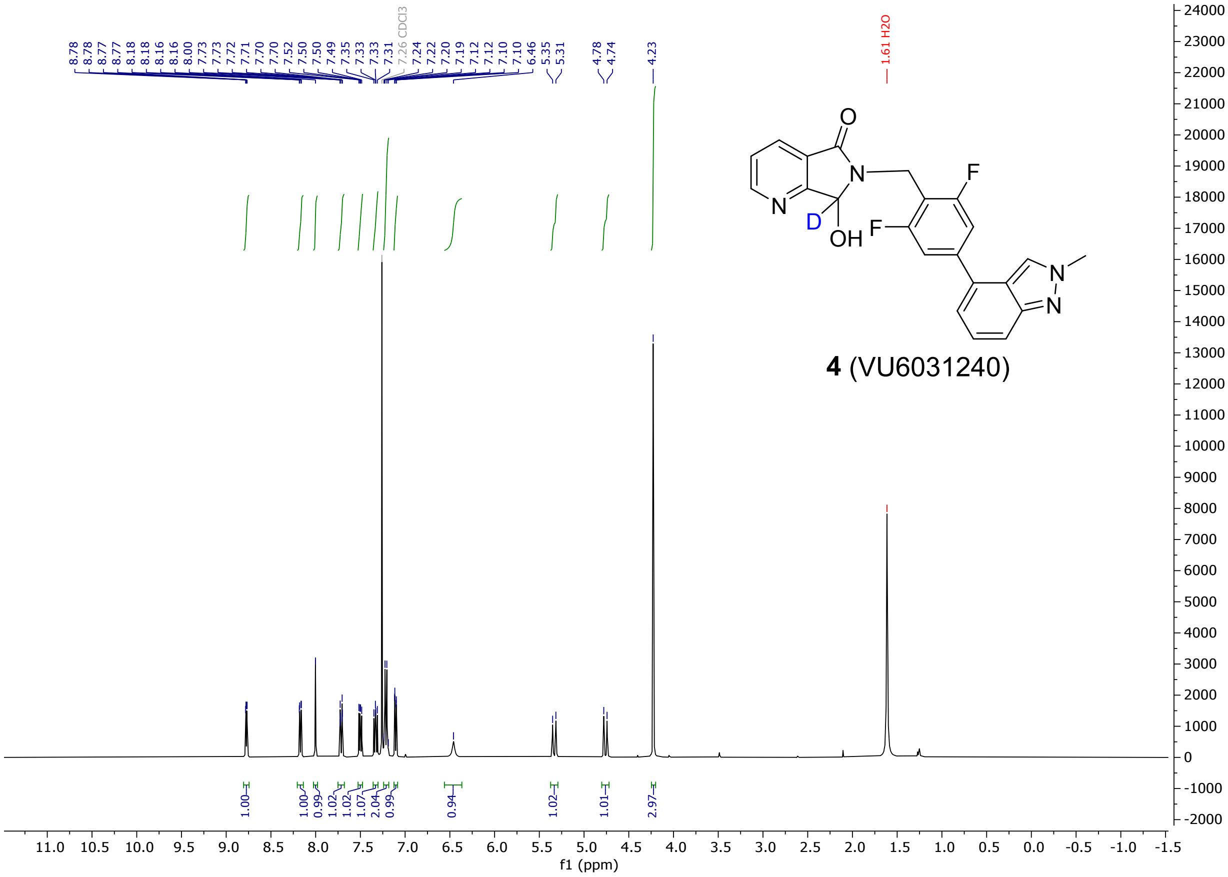

^{1}H NMR (400 MHz, $CDCl_3$) δ 8.77 (dd, J = 5.0, 1.6 Hz, 1H), 8.17 (dd, J = 7.7, 1.5 Hz, 1H), 8.00 (s, 1H), 7.71 (dt, J = 8.7, 0.9 Hz, 1H), 7.50 (dd, J = 7.7, 5.1 Hz, 1H), 7.33 (dd, J = 8.7, 6.9 Hz, 1H), 7.25 – 7.18 (m, 2H), 7.11 (dd, J = 6.9, 0.8 Hz, 1H), 6.46 (s, 1H), 5.33 (d, J = 14.5 Hz, 1H), 4.76 (d, J = 14.7 Hz, 1H), 4.23 (s, 3H).

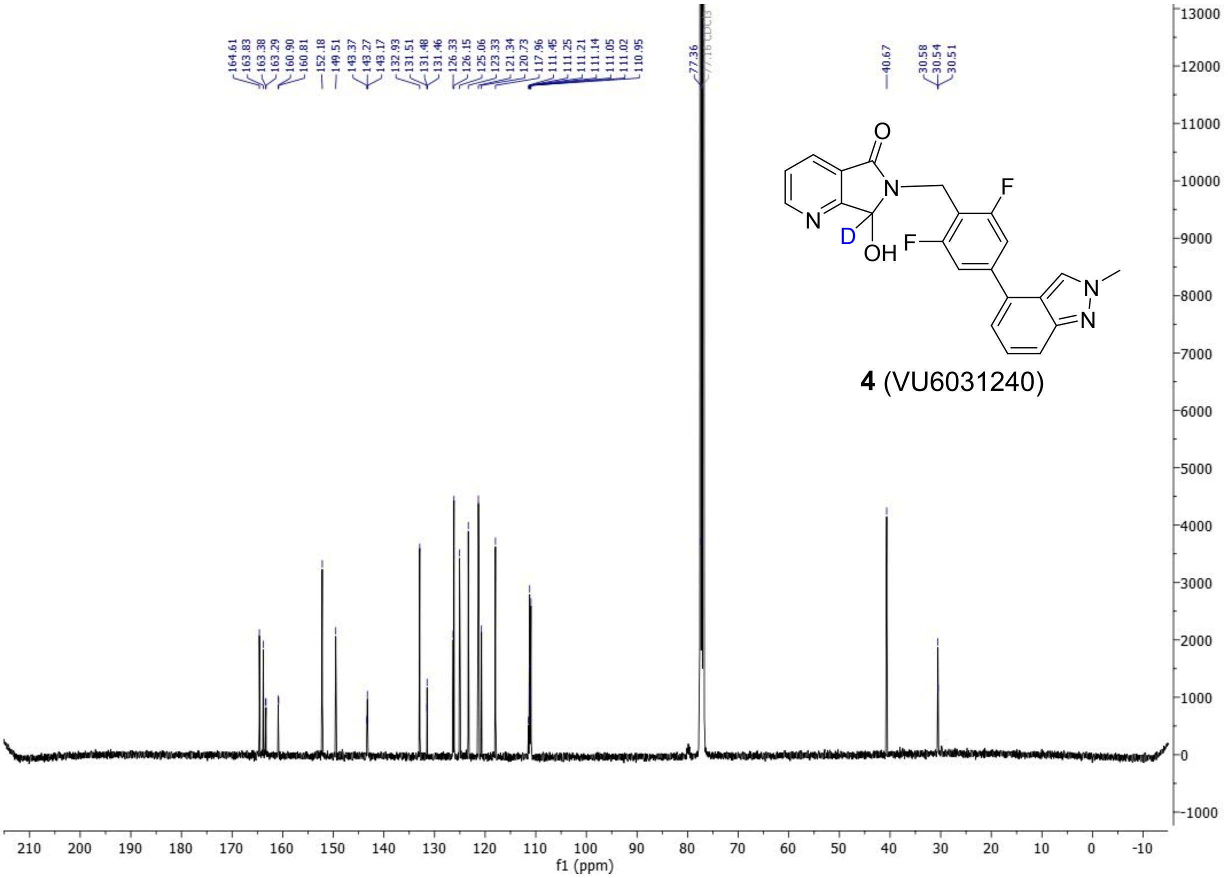

^{13}C NMR (101 MHz, $CDCl_3$) δ 164.61, 163.83, 163.34 (d, J = 8.9 Hz), 160.85 (d, J = 9.1 Hz), 152.18, 149.51, 143.27 (t, J = 10.0 Hz), 132.93, 131.49 (t, J = 2.5 Hz), 126.33, 126.15, 125.06, 123.33, 121.34, 120.73, 117.96, 111.55 – 110.77 (m, 3C), 77.36 (bs, CD_3), 40.67, 30.54 (t, J = 3.6 Hz).

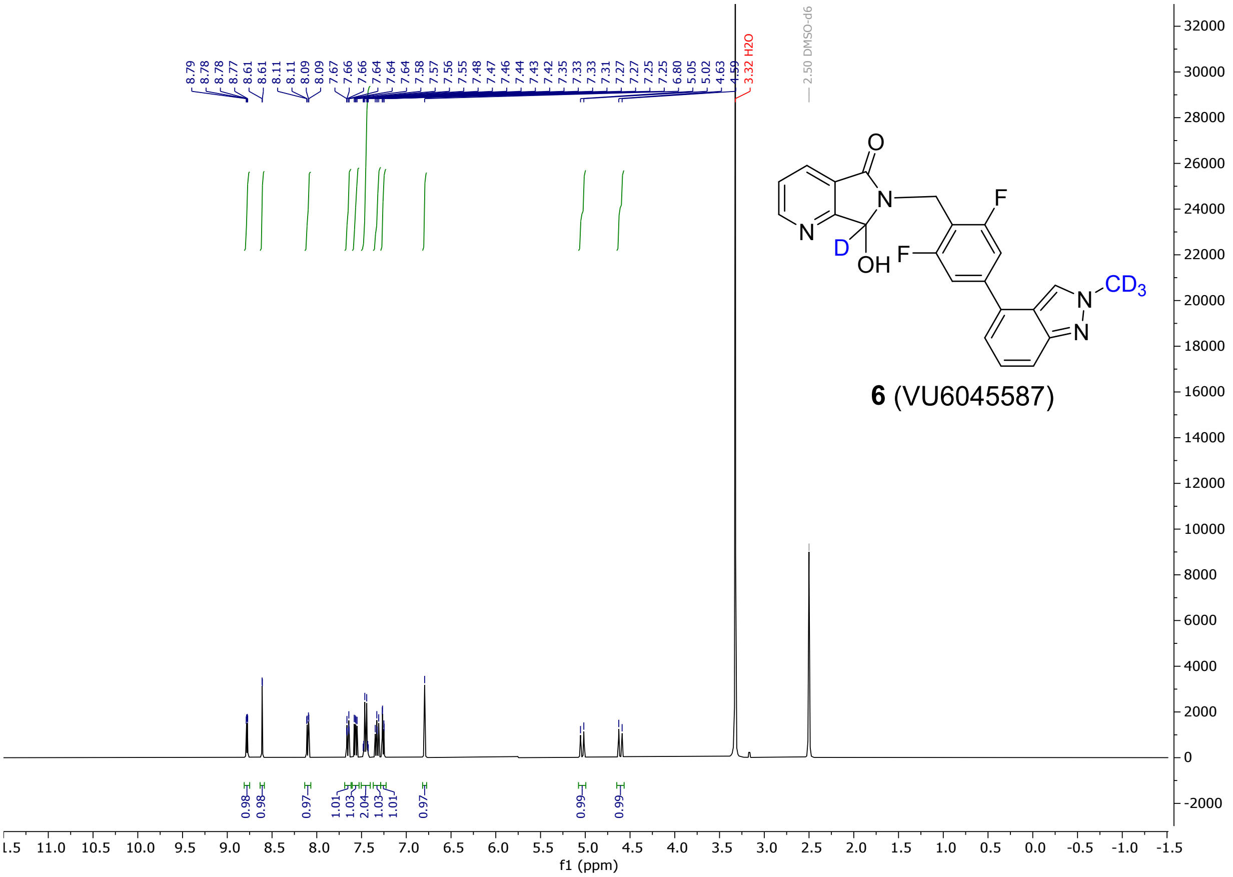

^1H NMR (400 MHz, DMSO-d_6) δ 8.78 (dd, J = 5.0, 1.6 Hz, 1H), 8.61 (d, J = 1.0 Hz, 1H), 8.10 (dd, J = 7.7, 1.6 Hz, 1H), 7.65 (dt, J = 8.6, 0.9 Hz, 1H), 7.57 (dd, J = 7.7, 5.0 Hz, 1H), 7.50 – 7.40 (m, 2H), 7.33 (dd, J = 8.5, 7.0 Hz, 1H), 7.26 (dd, J = 7.0, 0.9 Hz, 1H), 6.80 (s, 1H), 5.03 (d, J = 14.8 Hz, 1H), 4.61 (d, J = 14.9 Hz, 1H).

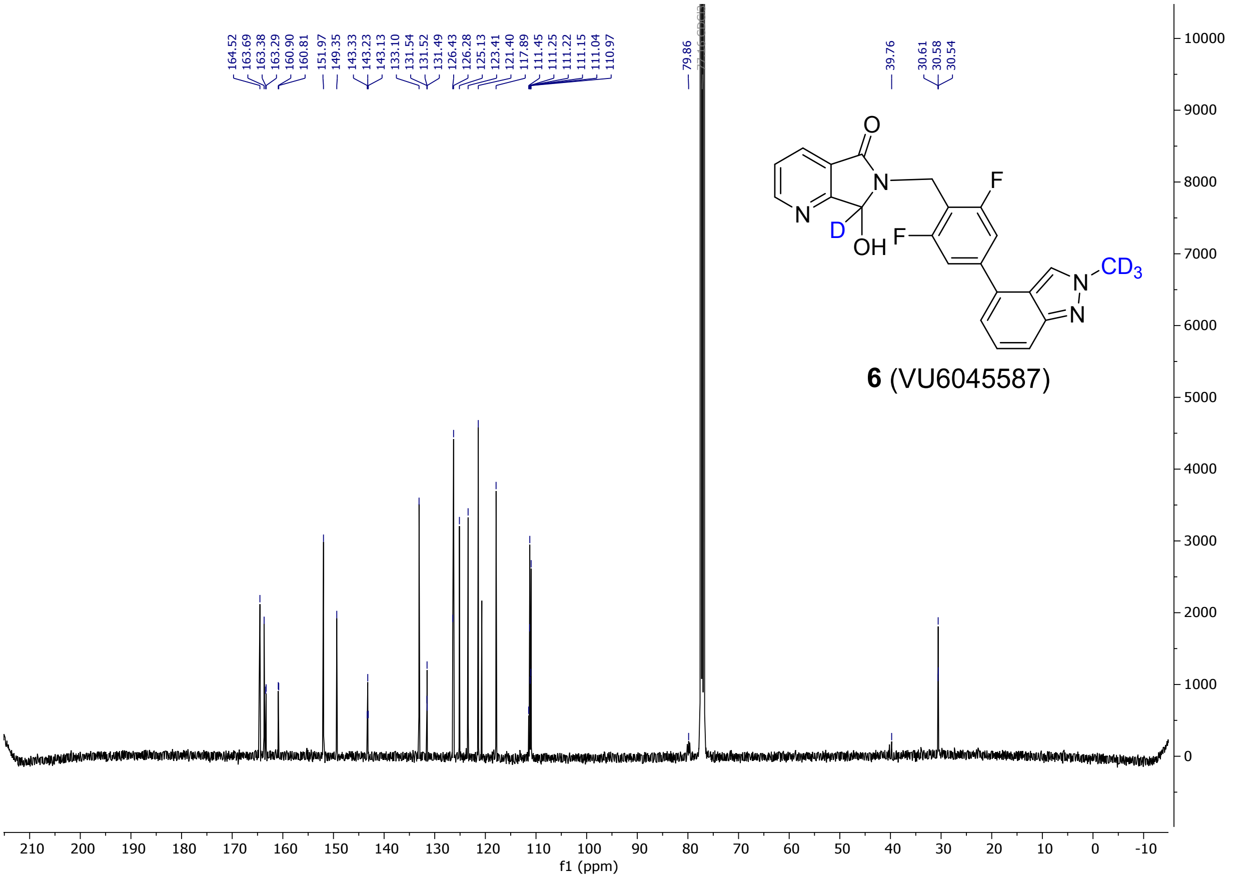

^{13}C NMR (101 MHz, $CDCl_3$) δ 164.52, 163.69, 163.34 (d, J = 8.9 Hz), 160.86 (d, J = 9.1 Hz), 151.97, 149.35, 143.23 (t, J = 10.1 Hz), 133.10, 131.52 (t, J = 2.4 Hz), 126.43, 126.28, 125.13, 123.41, 121.40, 117.89, 111.63 – 110.71 (m, 3C), 80.52 – 79.15 (m, CD_2), 40.84 – 39.17 (m, CD_3), 30.58 (t, J = 3.5 Hz).

Supplemental Figures

PO PK of VU6045422-02 in Rat

Project	M1 PAM
Study	PO PK
Animals	Rat SD, male (n=2)
Vehicle	(IV) 10% EtOH 40% PEG400 50% saline<br>(PO) 10% Tween80 in water
Dose	(IV) 0.2 mg/kg; (PO) 1 mg/kg
Matrix	EDTA plasma
Analyte	VU6045422-02
LLOQ	0.5 ng/mL

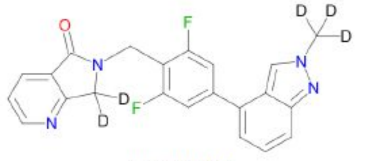

VU6045422-02

0.2 mg/kg IV VU6045422-02

Dose (mg/kg)	Time (hr)	Concentration (ng/mL) 1	2	Ave
	0.0333	1180	1262	1221
	0.117	970	1047	1008
	0.25	911	1177	1044
	0.5	677	715	696
1	1	497	623	560
	2	393	482	438
	4	216	288	252
	7	90.4	128	109
	24	3.60	6.66	5.13

1 mg/kg PO VU6045422-02

Dose (mg/kg)	Time (hr)	Concentration (ng/mL) 1	2	Ave
	0.25	401	443	422
	0.5	545	735	640
	1	441	689	565
1	2	448	492	470
	4	409	294	351
	7	306	179	243
	24	37.8	7.43	22.6

	PK Parameter		1	2	Ave
IV PK	$t_{1/2\ (term)}$	hr	3.47	3.78	3.63
IV PK	MRT	hr	3.73	4.11	3.92
Cassette	Cl obs	mL/min/kg	5.41	4.18	4.80
	Vss	L/kg	1.21	1.03	1.12
	AUC (IV)	hr*ng/mL	3078	3984	3531
PO PK	Cmax	ng/mL	545	735	640
	Tmax	hr	0.50	0.50	0.50
	AUC (PO)	hr*ng/mL	6025	4269	5147
	F	%	170.6	120.9	146

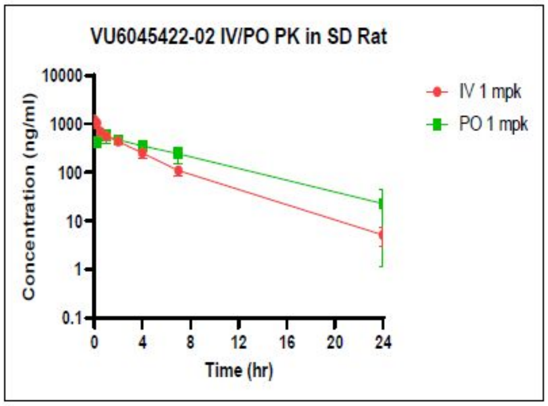

Figure S1. Rat IV/PO PK with VU6045422 (5).

Proposed Metabolic Pathways of VU6045422 in Human Liver Microsomes

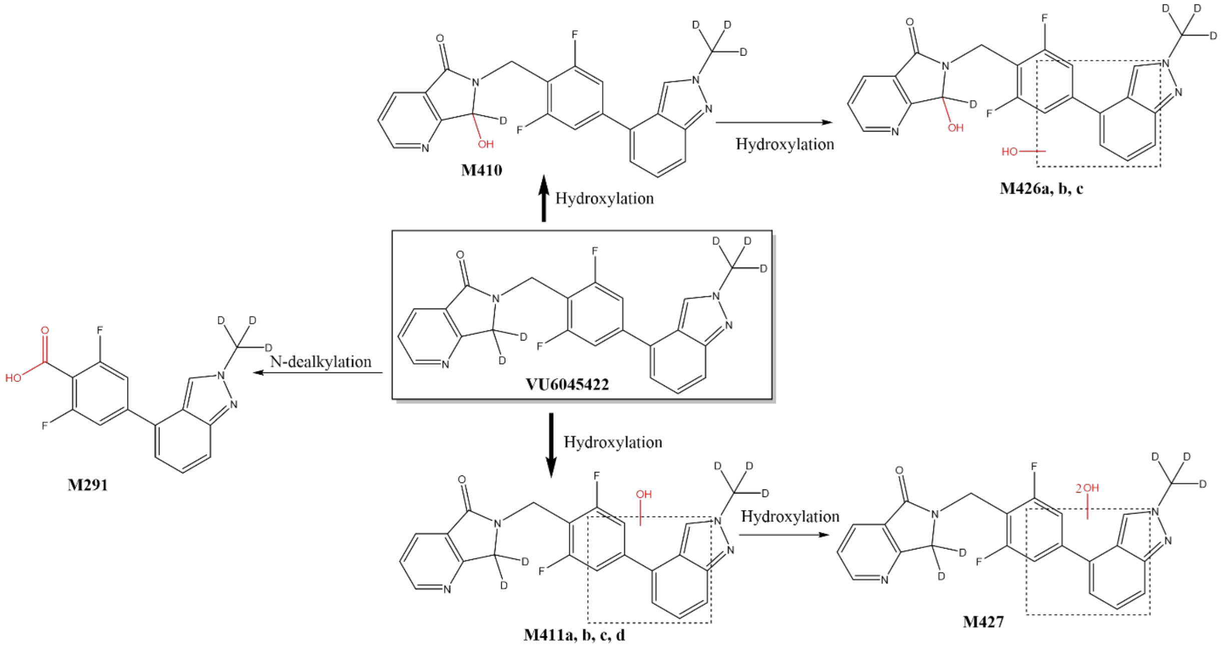

Figure S2. Proposed metabolic pathways of VU6045422 (5) in human liver microsomes.

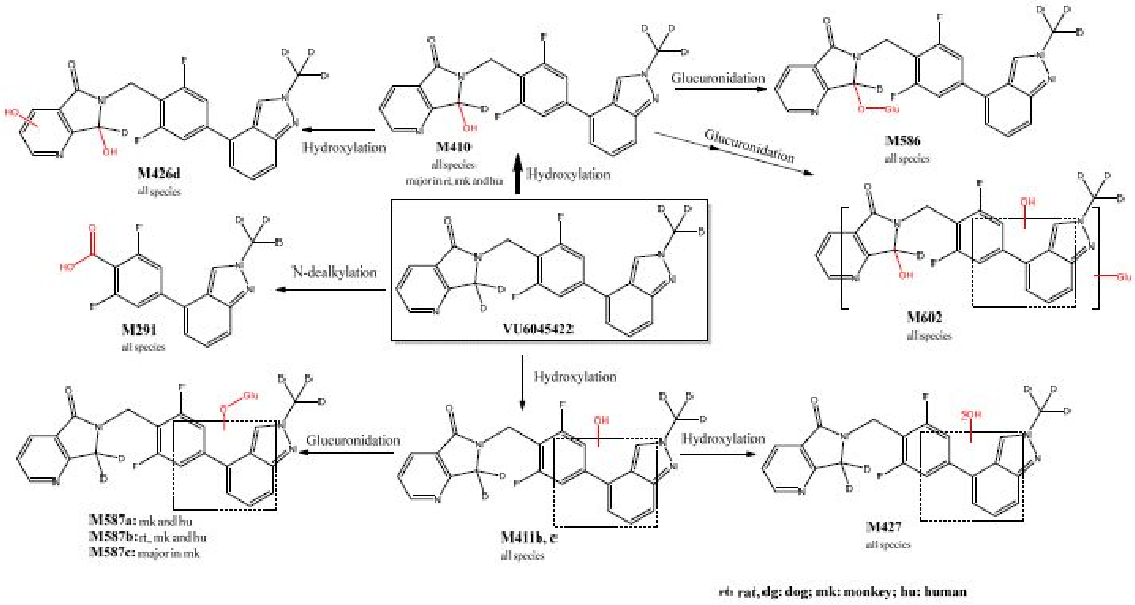

Figure S3. Proposed metabolic pathways of VU6045422 (5) in rat, dog, monkey and human hepatocytes.

Test Article ID	IC_{50} (μM)	Conc (μM)	Mean % hERG Inhibition	Standard Deviation	Standard Error	n	Individual Data Points (% Inhibition)
VU6045422-01	20.972	1	2.9	3.5	1.6	5	3.2
							4.0
							-2.1
							7.6
							1.9
		3	8.6	4.8	2.2	5	7.9
							14.6
							2.9
							12.2
							5.2
		10	27.3	7.6	3.4	5	28.5
							40.0
							23.0
							24.1
							21.0
		30	59.4	9.0	4.0	5	67.0
							69.3
							58.2
							54.7
							47.5
		100	92.9	3.8	1.7	5	96.8
							86.6
							94.4
							93.6
							93.4

Figure S4. Effects of VU6045422 (5) on hERG Ion Channel Current.

Compound I.D.	Client Compound I.D.	Test Concentration	%Control 1st	%Control 2nd	%Control 3rd	Mean %Control	Cytotoxicity (% of control)	Flags 1st	Flags 2nd	Flags 3rd
Bacterial cytotoxicity (TA98 - S9)										
100055946-1	VU6045422-01	6.0E-07 M	93.1	96.7	96.7	**95.5**	95			
100055946-1	VU6045422-01	1.2E-06 M	83.7	83.7	88.4	**85.3**	85			
100055946-1	VU6045422-01	2.5E-06 M	83.7	94.3	89.6	**89.2**	89			
100055946-1	VU6045422-01	5.0E-06 M	83.7	97.8	87.2	**89.6**	90			
100055946-1	VU6045422-01	1.0E-05 M	84.9	97.8	90.8	**91.2**	91			
100055946-1	VU6045422-01	2.5E-05 M	84.9	95.5	93.1	**91.2**	91			
100055946-1	VU6045422-01	5.0E-05 M	82.5	87.2	87.2	**85.7**	86			
100055946-1	VU6045422-01	1.0E-04 M	108.4	77.8	87.2	**91.2**	91			
Bacterial cytotoxicity (TA100 - S9)										
100055946-1	VU6045422-01	6.0E-07 M	90.5	95.8	87.9	**91.4**	91			
100055946-1	VU6045422-01	1.2E-06 M	89.2	87.9	85.3	**87.5**	87			
100055946-1	VU6045422-01	2.5E-06 M	85.3	81.3	81.3	**82.6**	83			
100055946-1	VU6045422-01	5.0E-06 M	87.9	81.3	93.2	**87.5**	87			
100055946-1	VU6045422-01	1.0E-05 M	84.0	82.6	82.6	**83.1**	83			
100055946-1	VU6045422-01	2.5E-05 M	91.9	89.2	87.9	**89.7**	90			
100055946-1	VU6045422-01	5.0E-05 M	89.2	86.6	85.3	**87.0**	87			
100055946-1	VU6045422-01	1.0E-04 M	89.2	84.0	81.3	**84.8**	85			
Bacterial cytotoxicity (TA1535 - S9)										
100055946-1	VU6045422-01	6.0E-07 M	129.5	32.6	123.4	**126.5**	126		{}	
100055946-1	VU6045422-01	1.2E-06 M	109.3	105.3	127.5	**114.0**	114			
100055946-1	VU6045422-01	2.5E-06 M	111.3	117.4	99.2	**109.3**	109			
100055946-1	VU6045422-01	5.0E-06 M	111.3	113.3	129.5	**118.0**	118			
100055946-1	VU6045422-01	1.0E-05 M	117.4	119.4	115.4	**117.4**	117			
100055946-1	VU6045422-01	2.5E-05 M	131.5	135.5	101.2	**122.8**	123			
100055946-1	VU6045422-01	5.0E-05 M	143.6	137.6	133.5	**138.2**	138			
100055946-1	VU6045422-01	1.0E-04 M	127.5	163.8	171.9	**154.4**	154			
Bacterial cytotoxicity (TA1537 - S9)										
100055946-1	VU6045422-01	6.0E-07 M	102.1	107.7	90.9	**100.2**	100			
100055946-1	VU6045422-01	1.2E-06 M	82.6	84.0	104.9	**90.5**	90			
100055946-1	VU6045422-01	2.5E-06 M	88.1	78.4	93.7	**86.7**	87			
100055946-1	VU6045422-01	5.0E-06 M	84.0	88.1	82.6	**84.9**	85			
100055946-1	VU6045422-01	1.0E-05 M	67.2	86.7	72.8	**75.6**	76			
100055946-1	VU6045422-01	2.5E-05 M	99.3	92.3	88.1	**93.3**	93			
100055946-1	VU6045422-01	5.0E-05 M	96.5	96.5	93.7	**95.6**	96			
100055946-1	VU6045422-01	1.0E-04 M	68.6	99.3	90.9	**86.3**	86			

Notes:

1. Cytotoxicity is presented as % of control growth.
2. A cytotoxicity value of less than 60 % is flagged, and the compound is considered as toxic at the respective concentration.

Figure S5. Genetic toxicity test (AMES) with VU6045422 (5).

Isotope Analysis

WARREN CENTER FOR NEUROSCIENCE DRUG DISCOVERY at Vanderbilt University

**Data Filename**	VU6045422-05_0002.d	**Sample Name**	VU6045422-05
**Sample Type**	Sample	**Position**	P1-D3
**Instrument Name**	Q-TOF	**User Name**	Christopher Presley
**Acq Method**	Auto MS_1x50_5-95_90sec.m	**Acquired Time**	7/20/2021 11:21:51 AM (UTC-05:00)
**IRM Calibration Status**	Success	**DA Method**	FBF_Deuterium-Exchange.m
**Comment**	0.3 µL Injection, JLE-18-183-Final		

**Sample Group**		**Info.**	
**Molecular Formula**	C22H11D5F2N4O	**Stream Name**	LC 1
**Acquisition Time (Local)**	7/20/2021 11:21:51 AM (UTC-05:00)	**Acquisition SW Version**	6200 series TOF/6500 series Q-TOF 10.1 (48.0)
**QTOF Driver Version**	10.01.00	**QTOF Firmware Version**	10.811
**Tune Mass Range Max.**	3200		

Compound	Height	Height%	Area	Area%
396.1679	1099910.23	98.69	3026122.28	98.58
395.1617	13871.18	1.24	42066.1	1.37
394.1554	681.33	0.06	1488.2	0.05

Values Adjusted for Overlapping Isotopic Abundances

Compound	Height	Height%	Area	Area%
396.1679	1096629.09	98.70	3016154.66	98.59
395.1617	13709.02	1.23	41711.91	1.36
394.1554	681.33	0.06	1488.2	0.05

Chromatograms

Fragmentor Voltage 150 **Collision Energy** 0 **Ionization Mode** ESI

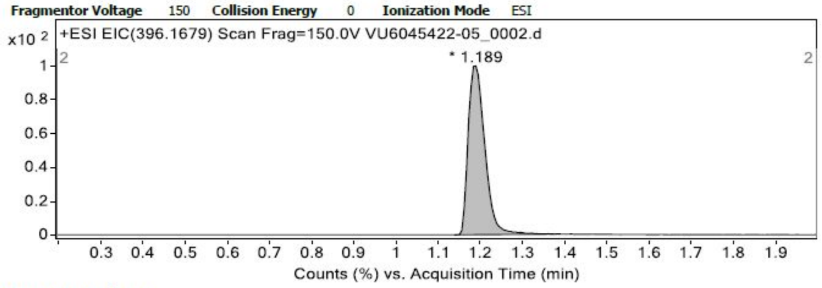

Integration Peak List

Peak	Start	RT	End	Height	Area	Area %
1	1.14	1.189	1.377	1099910.23	3026122.28	100

Figure S6. Isotope analysis of VU6045422 (5).

HRMS/Accurate Mass Report

**Data File**	VU6045422-05_0004.d	**Sample Name**	VU6045422-05
**Sample Type**	Sample	**Position**	P1-D3
**Instrument Name**	Q-TOF	**User Name**	Christopher Presley
**Acq Method**	Auto MSMS_1x50_5-95_90sec.m	**Acquired Time**	7/20/2021 3:09:52 PM (UTC-05:00)
**IRM Calibration Status**	Success	**DA Method**	Default Report AMM_210224.m
**Comment**	0.3 µL Injection, JLE-18-183-Final		

**Sample Group**		**Info.**	
**Molecular Formula**	C22H11D5F2N4O	**Stream Name**	LC 1
**Acquisition Time (Local)**	7/20/2021 3:09:52 PM (UTC-05:00)	**Acquisition SW Version**	6200 series TOF/6500 series Q-TOF 10.1 (48.0)
**QTOF Driver Version**	10.01.00	**QTOF Firmware Version**	10.811
**DDE Mode**	2	**Tune Mass Range Max.**	3200

Compound Table

Compound Label	RT	Mass m/z - addt	Abund	Name	Formula	Mass TGT	Diff (ppm)	Hits (DB)
Cpd 1: VU6045422-05; C22 H11 D5 F2 N4 O; 1.193	1.193	395.1614	9656	VU6045422-05	C22 H11 D5 F2 N4 O	395.1606	1.93	1

Compound Label	Name	m/z Observed	RT	Algorithm
Cpd 1: VU6045422-05; C22 H11 D5 F2 N4 O; 1.193	VU6045422-05	396.1686	1.193	Find by Formula

Calc m/z	Ion
396.1679	(M+H)+

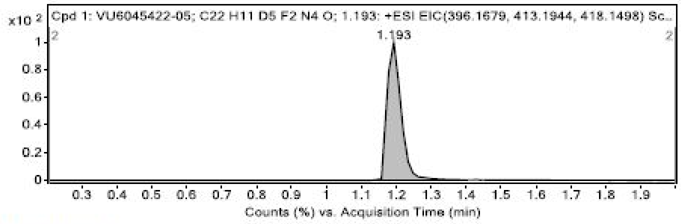

MS Zoomed Spectrum

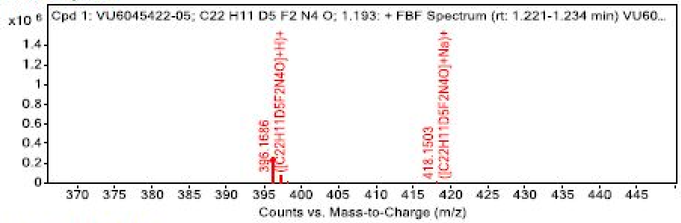

MS Spectrum Peak List

m/z	z	Abund	Formula	Ion
396.1686	1	260418.92	C22H11D5F2N4O	(M+H)+
397.172	1	63361.3	C22H11D5F2N4O	(M+H)+
398.1741	1	7397.25	C22H11D5F2N4O	(M+H)+
418.1503	1	9655.93	C22H11D5F2N4O	(M+Na)+
419.1524	1	2506.78	C22H11D5F2N4O	(M+Na)+
420.1535	1	372.1	C22H11D5F2N4O	(M+Na)+

Figure S7. HRMS/Accurate Mass Measurement of VU6045422 (5).
